# Chitosan mitigates pan drug resistance in citrobacter freundii exhibiting AmpC and ESBL from Egyptian livestock

**DOI:** 10.1038/s41598-025-28607-0

**Published:** 2025-12-05

**Authors:** Ibtisam Faeq Hasona, Gamal Younis, Amal Awad

**Affiliations:** Department of Bacteriology, Mycology, and Immunology, Faculty of Veterinary Medicine, Mansoura University, Mansoura, 35516 Egypt

**Keywords:** Chickens, Buffalo, *Citrobacter freundii*, Chitosans, Antimicrobial resistance profiling, Ciprofloxacin enhancement, Biotechnology, Microbiology, Molecular biology

## Abstract

Extended-spectrum beta-lactamase (ESBL) and ampicillin resistance gene type C (ampC) in foodborne *Citrobacter freundii (C. freundii)* pose a severe public health risk, especially when they combine with other critical resistance genes to produce a reservoir of resistance. Therefore, the current study aimed to estimate the prevalence, phenotypic, and genotypic profiling of antimicrobial resistance in *C. freundii* isolates obtained from diseased broiler chickens and native Egyptian buffaloes in Kafr El-Sheikh and Dakahlia governorates, Egypt. Also, the investigation of the antibacterial activity of chitosan (CS) 1%, 2%, and chitosan nanoparticles (CSNPs), including the estimation of minimum inhibitory concentrations (MIC) and minimum bactericidal concentrations (MBC) and their combinations with ciprofloxacin (CIP), were evaluated. *C. freundii* was detected in 57.58% (19/33) of broiler chickens and 52.63% (20/38) of buffalo. Surprisingly, 26.32% (5/19) and 20% (4/20) of chicken and buffalo samples, respectively were multidrug-resistant (MDR), 42.11% (8/19) and 15% (3/20) were extensively drug-resistant (XDR), and 31.58% (6/19) and 65% (13/20) were pan drug-like-resistant (PDR). High frequencies of resistance genes were observed, including *int*1, *dfrA*1, *sul*2, *aad*A1, *qnr*A, and *bla*_TEM_ (≥ 80%), while other genes such as *erm*B, *bla*_CMY−2_, *bla*_OXA−10_, *tet*(M), *mcr-*1, *bla*_CTX−M,_ and *cat*A1 were detected at variable rates. Alarmingly, all ESBL/AmpC-producing isolates co-harbored multiple critical resistance genes. CSNPs showed markedly lower MIC and MBC values compared with 1% and 2% CS, indicating significantly stronger bactericidal activity. The concerning prevalence of PDR like-*C. freundii* in food animals is brought to light by our results, which also provide credence to the use of 2% CS–CIP and CSNPs–CIP combinations as potential substitute treatments for resistant infections.

**Supplementary Information:**

The online version contains supplementary material available at 10.1038/s41598-025-28607-0.

## Introduction

*Citrobacter* spp. have been related to nosocomial infections of the urinary tract, liver, biliary tract, peritoneum, intra-abdominal sepsis, bone, respiratory tract, endocardium, wounds, soft tissue, meninges, brain abscesses, pneumonia, and bloodstream infections, as well as neonatal infections including meningitis, neonatal sepsis, joint infections, or common bacteremia^1^. According to Moussa et al.^2^., *Citrobacter* spp. have also been linked to skin diseases, including necrotizing fasciitis, folliculitis, cellulitis, hives, and ulcers. *Citrobacter* spp. infections have been linked with a high mortality rate of 33–48% generally, especially 30% of fatalities occurring among kids^3^. These two main pathogens, *C. koseri* and *C. freundii*, are clearly responsible for the majority of *Citrobacter* infections, with over 80% of those infected suffering underlying medical issues that involve diabetes, cardiovascular disease, renal disease, leukemia, neurologic disease, or urinary tract abnormalities^4^. Although *Citrobacter freundii (C. freundii)* has been found in food, water, soil, and the digestive tracts of both people and animals, it may still cause a number of illnesses^5^. According to Liu et al.^6^., *C. freundii* also caused diarrhea or food poisoning in people. *C. freundii* is implicated in a variety of diseases in both buffalo and broiler chickens. In young or immunocompromised broilers, it can induce severe septicemia and hepatitis, presenting with lethargy, decreased feed intake, and high mortality rates^7^. Although clinical manifestations in buffaloes are less well-characterized, the bacterium is recognized as opportunistic and has been associated with septicemia, encephalitis, and hemorrhagic lesions in the lungs, liver, spleen, and intestines of other animal species^8^. Reports from Egypt indicate variable isolation rates of *C. freundii* in livestock: Ibrahim et al.^9^ detected 9.6% in buffalo milk samples from Sharkia Province; Ombarak and Elbagory^10^ reported 5.71% in Menoufia Province; Nossair et al.^11^ found 8% in buffalo meat from Behera Province; and Abd El-Tawab^12^ reported 13.46% (14/104) in recently deceased broiler chickens from Gharbia Governorate. Despite these prior reports, there is a notable lack of comprehensive studies that simultaneously examine the prevalence, phenotypic characteristics, and antimicrobial resistance patterns of *C. freundii* in livestock. The present investigation fills this critical gap by providing an integrated assessment of *C. freundii* in both broiler and buffalo farms across Egypt. By combining prevalence data with detailed phenotypic and resistance profiling, this study offers essential insights into the epidemiology of this pathogen and lays the groundwork for informed, evidence-based strategies to monitor, control, and prevent *C. freundii* infections in livestock populations.

Extensive antimicrobial usage in medical, veterinary, and agricultural treatment has considerably contributed to the finding and worldwide distribution of resistance genotypes in the *Enterobacteriaceae* family in the last few decades^13^. Specifically, expanded-spectrum antibiotics are haphazardly integrated into animal feed for both preventative and curative purposes, and subtherapeutic use of antimicrobial drugs in livestock can result in the spread of possibly resistant microbes in the surroundings, presenting an important safety concern towards humans^14^. According to estimates, bacterial antimicrobial resistance (AMR) caused 4.95 million fatalities worldwide in 2019; South Asian and sub-Saharan African nations now account for the majority of these fatalities^15^. *C. freundii*’s genetic variety is generated using processes including homologous recombination, deletions, point mutations, and horizontal gene transfer; all of these lead to phenotypic instability and antigenic variations^16^. This genetic flexibility enables *C. freundii* to quickly adjust to diverse environments and obstacles, among them antibiotic resistance and the potential to attack individuals as well as livestock^16^. *Citrobacter* species include plenty of antibiotic-resistant factors encoded in their chromosomes or plasmids^17^. This state provides a capability to convert non-pathogenic bacteria into resistant reservoirs in native bacterial ecosystems^18^.

*Citrobacter* strains are powerful carriers of plasmid-mediated quinolone resistance determinants (PMQR), broad-spectrum β-lactamase, extended-spectrum β-lactamase (ESBL), and chromosomally induced ampC β-lactamase^16^. Remarkably prevalent genotypes that encode ESBLs are *bla*_CTX−M_, *bla*_TEM_, *bla*_SHV_, and *bla*_OXA_^19^. One notable concern is the possibility that the ampC enzyme serves as a quiet, hidden repository for ESBLs, hindering their identification or medication as they cohabit within one isolate^20^. The presence of these genes in enteric bacteria makes them more resistant to several beta-lactam medications^21^, causing greater fatalities, morbidity, greater expenditures on healthcare, and fewer treatment alternatives. The significant fatality rate of *C. freundii* is specifically caused by inefficient antibiotic usage, considering this microbe encodes for ampC β-lactamase genes (*bla*_CMY_-like), which are linked to elevated resistance to several antibiotics, including extended-spectrum cephalosporins^22^. Additionally, it is due to intrinsic resistance against several commonly employed antibiotics, especially ampicillin-sulbactam, amoxicillin-clavulanic acid, first- and second-generation cephalosporins, and cephamycins^23^. The present emergence of multidrug-resistant *C. freundii* strains is causing alarm. Antibiotic resistance in this bacterium has been predominantly associated with ampC β-lactamases and ESBLs. On the other hand, carbapenem-resistant *Citrobacter* species (CRC) have become a significant problem recently^24,25^. Ambler class A β-lactamases (KPC), class B metallo-β-lactamases (NDM, IMP, and VIM), and class D β-lactamases (OXA-48) all contribute to carbapenem resistance^26^. Carbapenemases, which can hydrolyze nearly all β-lactam antibiotics, are mostly encoded on mobile plasmids^25^.

Class 1 integrons have been identified as important genetic elements contributing to the dissemination of antimicrobial resistance, which can lead to substantial bacterial outbreaks among animals worldwide^27^. According to Girlich et al.^28^., integrons, including the integrase gene *int*1, are genomic substrates allowing the synthesis of gene cassettes that express lactamases, antibiotic resistance genes, aminoglycoside resistance determinants, or PMQR genes. Owing to concerns about colistin misuse in humans and animals, which may drive resistance via zoonotic gene transfer, and its essential role in treating resistant Gram-negative infections, the WHO and other authorities classify colistin as a critically important antibiotic with restricted use^29^. Since *mcr-*1 was recently discovered in *C. freundii*^30^, pan-drug resistance might develop in *Citrobacter* species, especially when the *mcr-*1 gene merges with other resistance genes, for example, aminoglycosides^31^ and ESBL^32^, this could result in unsuccessful therapies and pose a public health threat.

According to Said and Abdelmegeed^33^, managing infectious illnesses is crucial for human health, particularly in light of the ongoing rise of multidrug- (MDR), extensively- (XDR), and even pan drug-resistant (PDR) infections. As such, there is an urgent demand for novel antimicrobial biological substances that are targeted, safe for veterinary usage, and ecologically sound as alternatives to antibiotics^34^. Chitosan is a white, rigid, inelastic, and nitrogen-containing polymer that is a naturally generated cationic nontoxic biopolymer (a linear polysaccharide consisting of 1–4 attached 2-amino-deoxy β-D-glucan) that is produced by partially deacetylating chitin^35^. Because chitosan has a wealth of organic components, is biocompatible, non-toxic, biodegradable, mucoadhesive, and has no adverse environmental impacts from bio-decomposition, it has been researched for advancement across many kinds of applications^36^. Xing et al.^37^. claim that chitosan nanoparticles (CSNPs) can exhibit important antibacterial qualities via various methods. These include the connection among negatively charged phospholipids in the plasma membrane and positively charged CSNPs, the capacity of CSNPs to chelate metals, their access to cell walls, and DNA suppression, which impedes the creation of mRNA. Chitosan exhibits strong antibacterial activity against a wide spectrum of food-spoiled and pathogenic microbes at concentrations ranging from 1% to 2%^38^.

Ciprofloxacin (CIP) is a well-known second-generation, broad-spectrum fluoroquinolone antibiotic with potent bactericidal activity against a wide range of clinically relevant bacteria^39^. After oral administration, CIP exhibits a bioavailability of approximately 70–80%^40^ and is widely distributed in body tissues, bones, and fluids, with an apparent volume of distribution of 1.7–5 L/kg^41^. Approximately 33% of CIP is eliminated non-renally as unchanged drug in feces and metabolites, while the majority is excreted renally^42^. Despite its broad-spectrum activity, the effectiveness of CIP can be limited by poor cellular penetration and other pharmacological constraints. Recent studies have demonstrated that the combination of CIP with CSNPs can enhance its antibacterial efficacy, exhibiting enhancement effects against various bacterial strains^43,44^. This approach not only helps overcome cellular uptake limitations but also has the potential to reduce side effects associated with conventional CIP therapy^45^. There is very little knowledge available on the zoonotic threat posed by *C. freundii* isolates from broiler chickens and buffaloes in Egypt, including neither antibiotic sensitivity nor genetic characterization of resistance genes. As a result, the major goals of the current research were to determine the frequency of *C. freundii* derived from broiler chickens and buffaloes in Kafr El-Sheikh and Dakahlia governorates in Egypt. Also, to evaluate their phenotypic and genotypic resistance to several classes of antimicrobial drugs, which are the primary sources of high pathogenicity in *C. freundii* isolates. The rising global issue of antibiotic resistance makes it more challenging to eradicate infections caused by *C. freundii*. Therefore, it highlights the pressing demand for new treatment techniques to tackle *C. freundii* resistance and to protect public health throughout the world. Expanding our present awareness of the impacts of antibacterial activities using growth inhibition, minimum inhibitory concentration (MIC), and minimum bactericidal concentration assessment (MBC) of CS 1%, CS 2%, and CSNPs on resistant *C. freundii* is therefore clinically significant. Additionally, an approach to enhancing benefits of chitosans with the presently critical commercial antibiotic CIP is being developed with the aim to find alternative therapies for resistant diseases.

## Results

### Prevalence of *C. freundii* among *Citrobacter* spp

**Fig. 1:**
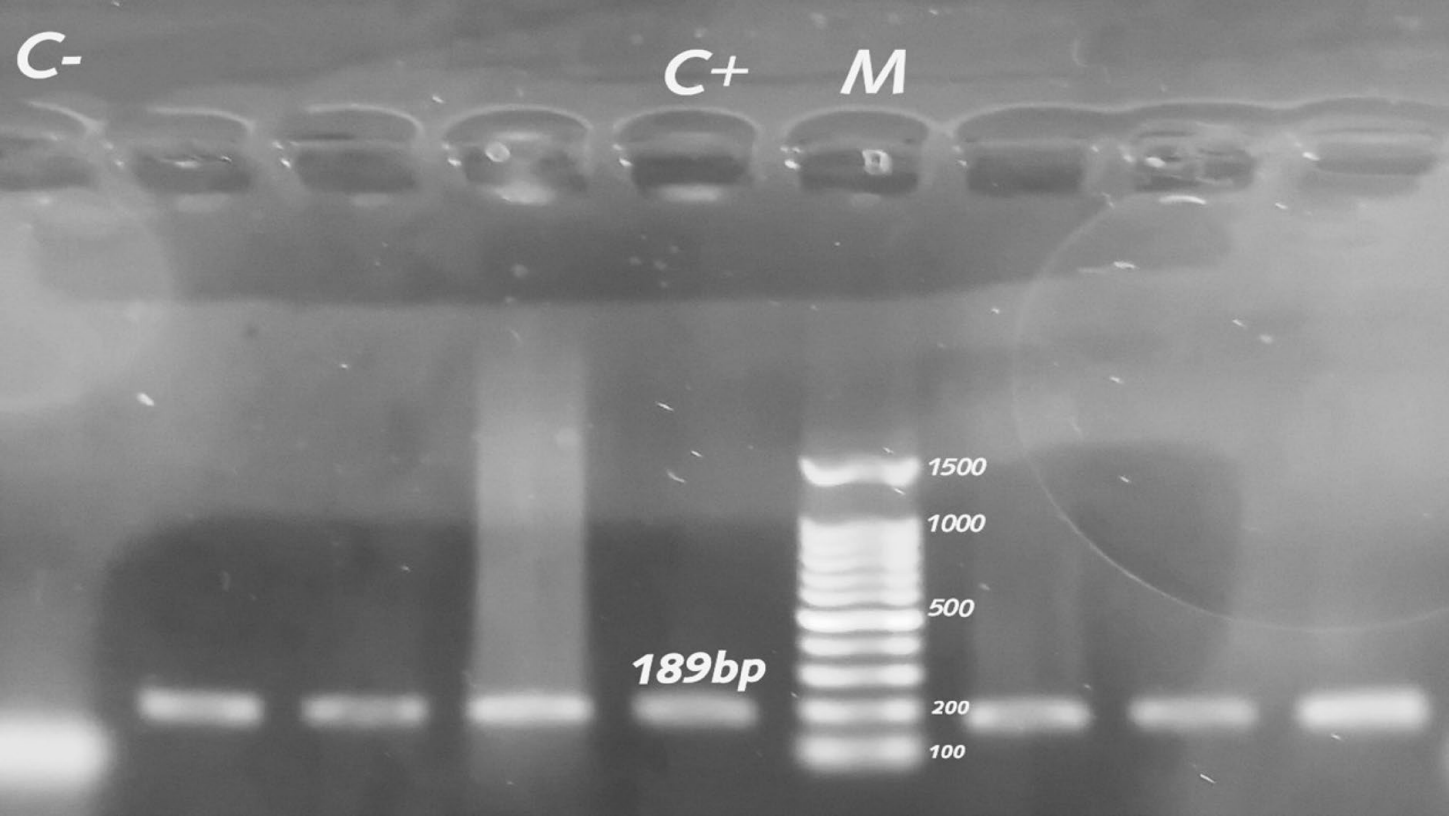
Agarose profile for the detection *C. freundii* specific 23 S rRNA is shown at 189 bp. Lane M: 100 bp ladder as a molecular size DNA marker. Lane C+: Control positive. C-: Control negative.

Affected chickens exhibited clinical signs including diarrhea, reduced feed intake, poor growth performance, ruffled feathers, and general weakness. Post-mortem examination revealed congested and dilated intestines with watery to mucoid contents, hepatomegaly with scattered petechial hemorrhages, mild splenomegaly, and congested kidneys. Emaciation and atrophied breast muscles were also evident in diseased birds. The isolates exhibited positive biochemical reactions characterized by hydrogen sulfide production, catalase and urease activity, motility, gas production, and typical triple sugar iron (TSI) reactions. In addition, the isolates were positive for Methyl Red test and citrate utilization, while showing negative reactions for oxidase, Indole, and Voges–Proskauer, which is consistent with the biochemical profile of *C. freundii*. *Citrobacter* spp. isolates from diseased broiler chickens and native Egyptian buffalo accounted for 10.48% (33/315) and 16.89% (38/225) of the 540 samples. Using standard PCR, all *Citrobacter* spp. isolates were analyzed for the *C. freundii* 23 S rRNA-specific gene (Fig. 1). Molecular screening revealed that the frequency of *C. freundii* isolates from diseased broiler chickens was greater (57.58%, 19/33) than that of Egyptian native buffalo isolates (52.63%, 20/38). Liu et al.^6^ detected 42 *Citrobacter* spp. in samples from foods and food handlers, including 26.19% (11/42) *C. freundii*, which is lower than our results. The percentage of broiler chickens that tested positive for *C. freundii* out of 33 isolates was as follows: meat 100% (3/3), liver 60% (3/5), gall bladder 66.67% (2/3), kidney 33.33% (2/6), gizzard 66.67% (4/6), intestine 66.67% (2/3), and cloacal swabs 60% (3/5). In native Egyptian buffalo samples, *C. freundii* isolates were found in the following: omasum 33.33% (1/3), abomasum 71.43% (5/7), reticulum 50% (2/4), rumen 50% (2/4), liver 20% (1/5), gall bladder 50% (1/2), meat 33.33% (2/6), jejunum 100% (3/3), and fecal matter 75% (3/4). Table 1 displays the distribution of *Citrobacter* spp. and *C. freundii* isolates across different sample sources.

**Table 1:** Distribution of *Citrobacter* spp. And *C. freundii* isolates among various samples.

Sample type	No. of Citrobacter spp. isolates (%)	No. of C. freundii isolates (%)
Broiler chickens		
	Total no. 33/315 (10.48)	Total no. 19/33 (57.58)
Meat	3/35 (8.57)	3/3 (100)
Liver	5/35 (14.29)	3/5 (60)
Lung	1/35 (2.86)	0/1 (0)
Gall bladder	3/35 (8.57)	2/3 (66.67)
Kidney	6/35 (17.14)	2/6 (33.33)
Gizzard	6/35 (17.14)	4/6 (66.67)
Intestine	3/35 (8.57)	2/3 (66.67)
Spleen	1/35 (2.86)	0/7 (0)
Cloacal swabs	5/35 (14.29)	3/5 (60)
Buffalo		
	Total no. 38/225 (16.89)	Total no. 20/38 (52.63)
Meat	6/25 (24)	2/6 (33.33)
Liver	5/25 (20)	1/5 (20)
Gall bladder	2/25 (8)	1/2 (50)
Reticulum	4/25 (16)	2/4 (50)
Rumen	4/25 (16)	2/4 (50)
Omasum	3/25 (12)	1/3 (33.33)
Abomasum	7/25 (28)	5/7 (71.43)
Jejunum	3/25 (12)	3/3 (100)
Fecal matter	4/25 (16)	3/4 (75)

### Profiling of phenotypic antimicrobial resistance of *C. freundii* isolates

The current investigation revealed that all broiler chicken-derived *C. freundii* isolates exhibited complete resistance to critically important antimicrobials, including gentamicin, nalidixic acid, ampicillin/sulbactam, amoxicillin, carbenicillin, and cefpodoxime, as well as to highly important antimicrobials like clindamycin and sulfamethoxazole/trimethoprim. Additionally, 94.74% of these isolates were resistant to critically important drugs including clarithromycin, aztreonam, and cefixime, alongside highly important agents like cefadroxil and minocycline. Resistance rates of 89.47%, 84.21%, 84.21%, 78.95%, 73.68%, and 68.42% were recorded for ciprofloxacin, azithromycin, piperacillin/tazobactam, levofloxacin, linezolid, and meropenem, respectively.

**Fig. 2:**
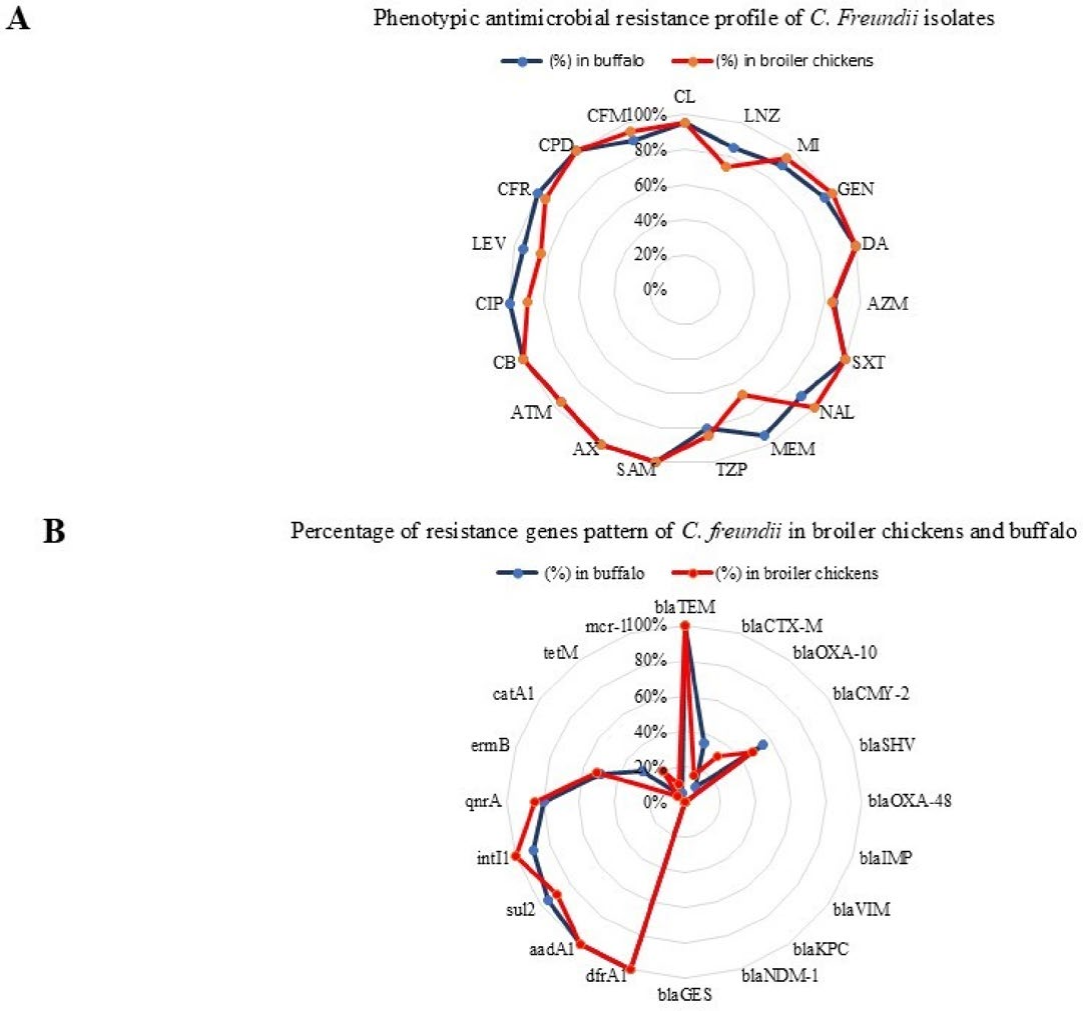
Overall antibiotic resistance pattern (**A**), and antibiotic resistance genes (**B**) of *C. freundii* isolates from broiler chickens, and buffalo.

**Fig. 3:**
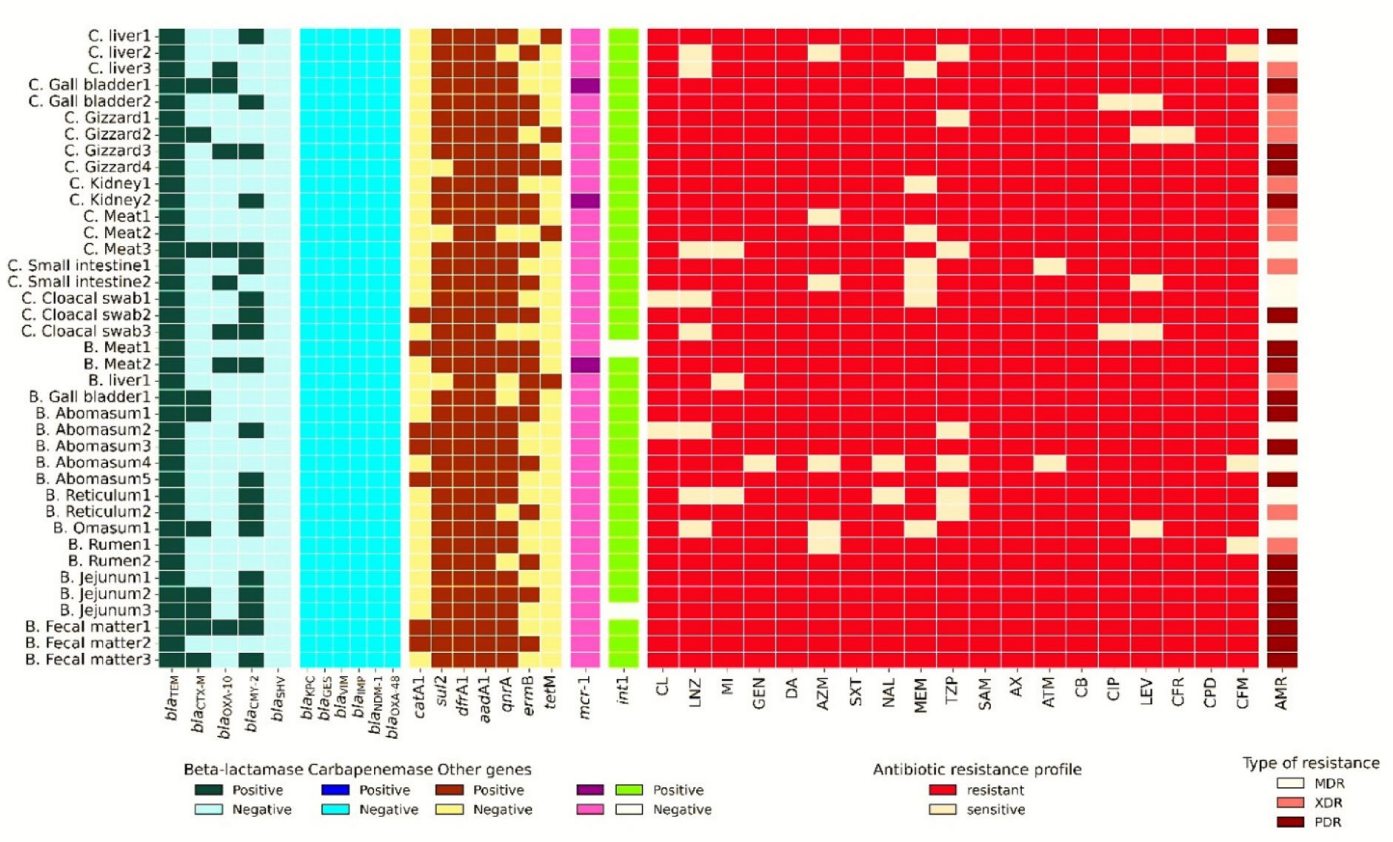
A heatmap representing association matrix between genotype and resistance phenotype as beta-lactamases genes (*bla*_TEM_, *bla*_CTX−M_, *bla*_OXA−10_, ampC type *(bla*_CMY−2_), and *bla*_SHV_), carbapenemase resistant genes (*bla*_KPC,_ *bla*_NDM−1,_ *bla*_OXA−48,_ *bla*_IMP,_ *bla*_GES,_ and *bla*_VIM_), other genes (*cat*A1, *sul*2, *dfr*A1, *aad*A1, *qnr*A, *erm*B, and *tet*(M)), colistin resistance gene (*mcr-*1), integron integrase gene (*int*1), resistance profile of each isolate to different antibiotics (clarithromycin (CL), linezolid (LNZ), minocycline (MI), gentamicin (GEN), clindamycin (DA), azithromycin (AZM), sulfamethoxazole/trimethoprim (SXT), nalidixic acid (NAL), Meropenem (MEM), piperacillin/tazobactam (TZP), ampicillin/sulbactam (SAM), amoxicillin (AX), aztreonam (ATM), carbenicillin (CB), ciprofloxacin (CIP), levofloxacin (LEV), cefadroxil (CFR), Cefixime, (CFM), cefpodoxime (CPD)), and type of resistance.

Similarly, buffalo-derived *C. freundii* isolates exhibited complete resistance (100%) to critically important antimicrobials such as ampicillin/sulbactam, amoxicillin, carbenicillin, ciprofloxacin, and cefpodoxime, as well as to highly important antimicrobials including clindamycin, sulfamethoxazole/trimethoprim, and cefadroxil. Extremely high resistance rates of 95% were observed for clarithromycin, gentamicin, meropenem, aztreonam, and levofloxacin. Furthermore, resistance rate of 90% were resistant to highly important antimicrobials such as minocycline, as well as critically important drugs including cefixime and nalidixic acid. Resistance to linezolid, azithromycin, and piperacillin/tazobactam was 85%, 85%, and 80%, respectively. Figures 2A and 3 display the overall antimicrobial resistance profiles. Interestingly, 100% (19/19) of the *C. freundii* isolates from broiler chickens exhibited beta-lactamase characteristics based on their antibiotic resistance profiles. Table 2 summarizes the multiple antibiotic resistance (MAR) indices of *C. freundii* isolates obtained from broiler chickens and buffaloes. The MAR indices ranged from 0.692 to 1.0 among broiler isolates and from 0.538 to 1.0 among buffalo isolates. Notably, 13 of 20 buffalo isolates (65%) and 6 of 19 broiler isolates (31.58%) exhibited resistance to all tested antimicrobials. Although these isolates were initially considered pan-drug resistant (PDR), this terminology was revised to PDR-like because the antimicrobial panel excluded last-resort agents such as tigecycline and contained redundant drugs from the same classes. The maximum MAR index of 1.0 was exclusively associated with PDR-like isolates, occurring more frequently among buffalo-derived isolates than broiler-derived isolates.

**Table 2:** Distribution of MAR indices among MDR, XDR, and PDR-like *C. freundii* isolates from broiler chickens and buffaloes.

Source	Resistance Type	No. of Resistant Isolates	% of Isolates	Antibiotic Classes Resistant	MAR Index Range
Broiler Chicken	MDR	5	26.32%	9–10	0.692–0.769
Broiler Chicken	XDR	8	42.11%	11–12	0.846–0.923
Broiler Chicken	PDR-like	6	31.58%	13	1
Buffalo	MDR	4	20%	7–10	0.538–0.769
Buffalo	XDR	3	15%	11–12	0.846–0.923
Buffalo	PDR-like	13	65%	13	1

### Detection of ESBL, AmpC, and carbapenem encoding genes

**Fig. 4:**
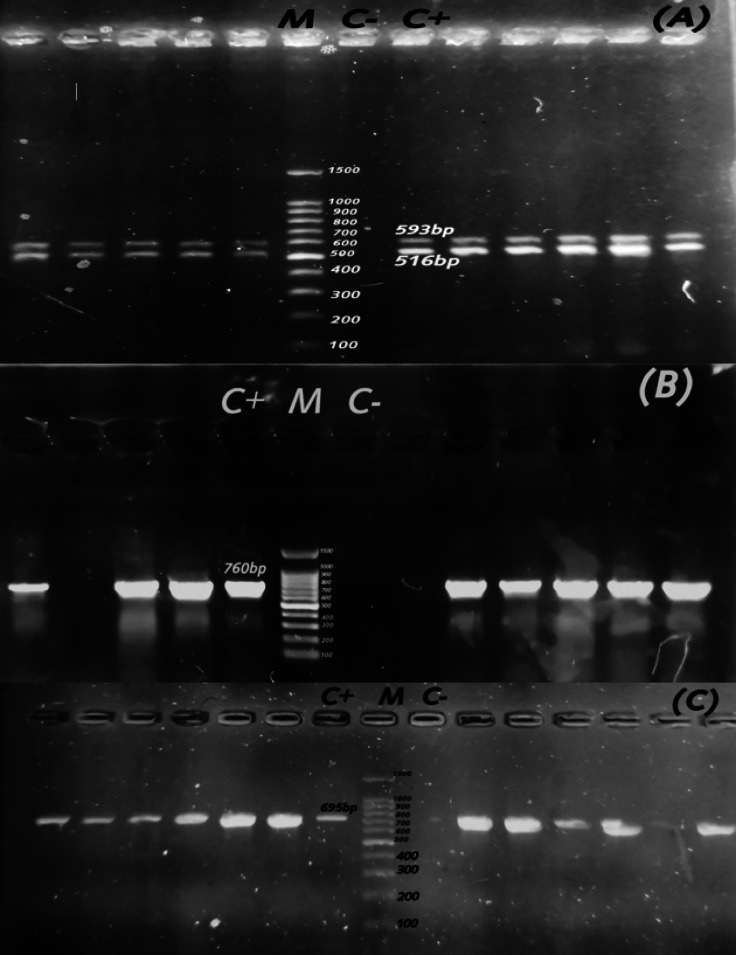
Agarose profile for the detection of resistance gene (**A**) duplex PCR of *bla*_CTX−M_ (593 bp), *bla*_TEM_ (516 bp); (**B**) *bla*_OXA−10_ (760 bp); and (**C**) *bla*_CMY−2_ (695 bp). Lane M: 100 bp ladder as a molecular size DNA marker. Lane C+: Control positive. C-: Control negative.

**Fig. 5:**
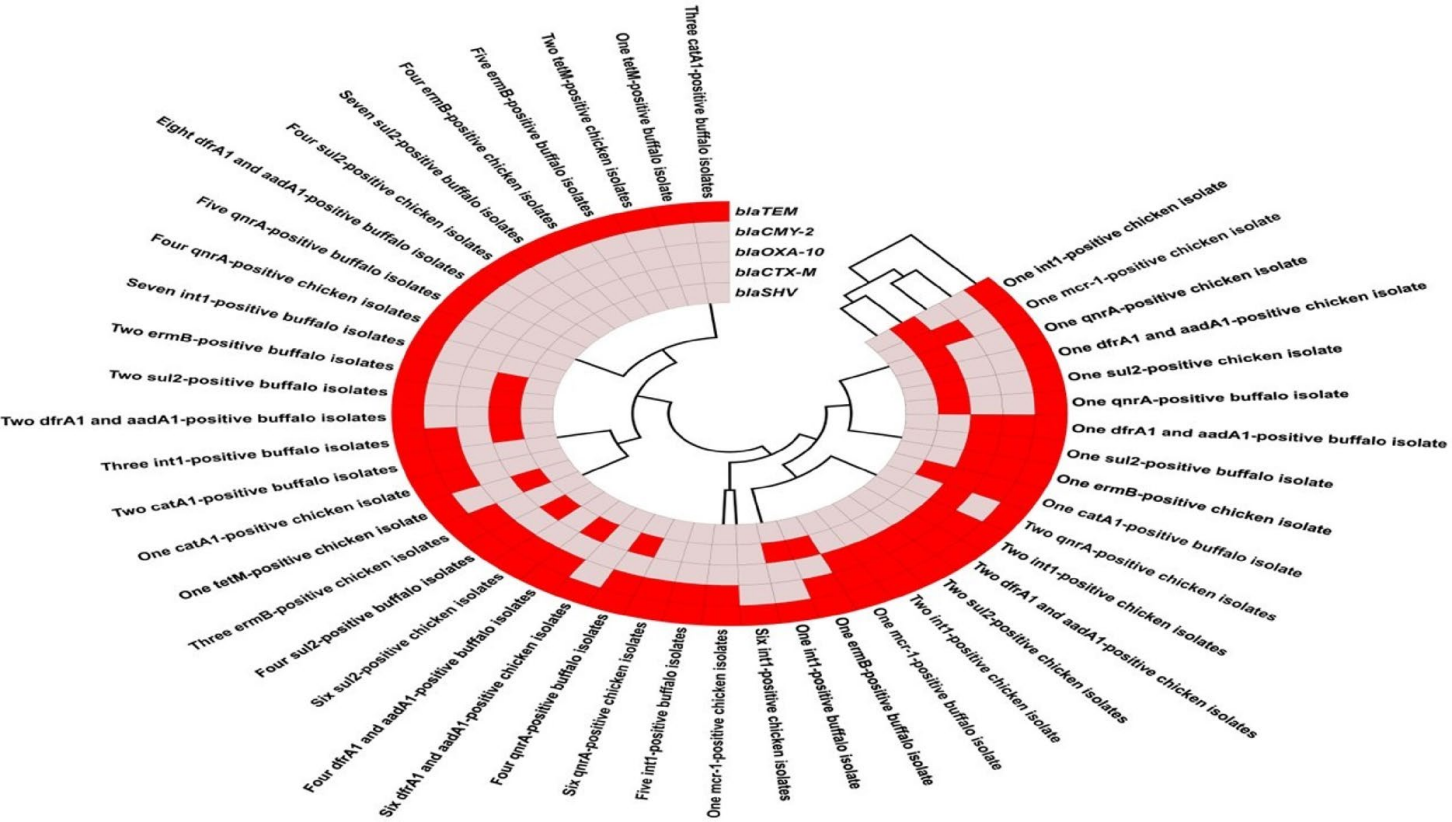
A circular heatmap representing the distribution of the association between ESBL/ampC and the other resistance genes. Red color represents a presence, and pale pink color represents an absence.

The percentage of resistance genes was observed in broiler chickens-originated *C. freundii* isolates as 100% *bla*_TEM_, 31.58% *bla*_OXA−10_, 15.79% *bla*_CTX−M_, 0% *bla*_SHV_, and 47.37% ampC (*bla*_CMY−2)_. All of the above resistance genes were observed in buffalo-originated *C. freundii* isolates as 100%, 10%, 35%, 0%, and 55%, respectively. The detailed distribution of the phenotypic and genotypic resistance profile from all the *C. freundii* isolates in the study is shown at Figs. 2 and 3. Also, Fig. 4 demonstrates an agarose profile for the identification of ESBL encoding genes and ampC encoding resistance genes. Upon comparing the prevalence of beta-lactamase genes among beta-lactamase-producing *C. freundii* isolates, alarmingly, the current study found that all samples from both broiler chickens (100%) and buffalo (100%) were also classified as beta-lactamase producers based on their characteristics and genotyping. This agrees with the high prevalence detected for *bla*_TEM_ (100%) in both broiler chickens and buffalo. These genes coexisted in multiple isolates. A circular heatmap depicts the full range of the connection between ESBL/ampC and the other resistance genes (Fig. 5). Although all *C. freundii* isolates in the present study were confirmed to harbor ESBL- and ampC-encoding genes (including *bla*_TEM_, *bla*_OXA_, *bla*_CTX−M_, *bla*_SHV_, and *bla*_CMY−2_), none of them tested positive for the common carbapenemase determinants (*bla*_KPC,_ *bla*_NDM−1,_ *bla*_OXA−48,_ *bla*_IMP,_ *bla*_GES,_ and *bla*_VIM_).

### Detection of association between ESBL/ampC and integron integrase class 1 genes

**Fig. 6:**
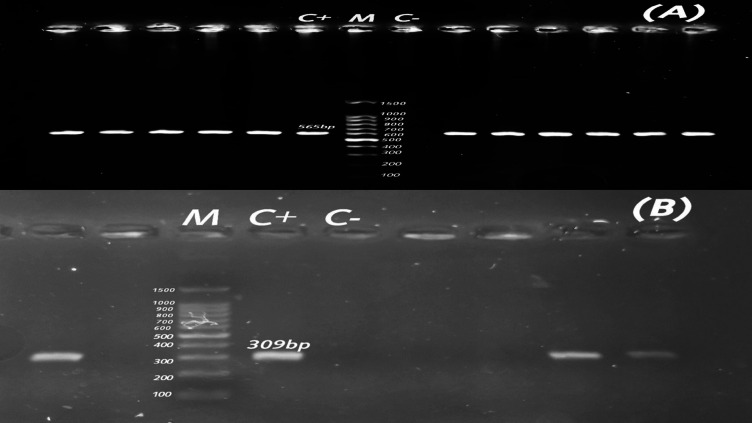
Agarose profile for the detection of resistance gene (**A**) *int*1 (565 bp), and (**B**) *mcr-*1 (309 bp). Lane M: 100 bp ladder as a molecular size DNA marker. Lane C+: Control positive. C-: Control negative.

Our investigation revealed the presence of *int*1 in 100% of broiler chicken isolates and 90% of buffalo isolates, which was an exciting discovery. An agarose profile for the identification of the *int*1 resistance gene was shown in Fig. 6A. The frequency of *int*1 positivity in broiler chicken-originated *C. freundii* isolates was significant among 6 *bla*_TEM_- and *bla*_CMY−2_-producing isolates and 6 *bla*_TEM−_producing isolates, 2 *bla*_TEM,_ *bla*_OXA−10−,_ and *bla*_CMY−2−_ producing isolates, 2 *bla*_TEM−_ and *bla*_OXA−10_-producing isolates, 1 *bla*_TEM−,_ *bla*_CTX−M−,_ *bla*_OXA−10−,_ and *bla*_CMY−2−_ producing isolate, 1 *bla*_TEM−,_ *bla*_CTX−M−,_ and *bla*_OXA−10−_ producing isolate; also, one isolate possessed *int*1 and produced both *bla*_TEM_ and *bla*_CTX−M_. While the prevalence of *int*1 in buffalo-originated *C. freundii* isolates was significantly observed among 7 *bla*_TEM_-producing isolates, 5 *bla*_TEM−_ and *bla*_CMY−2−_producing isolates, 3 *bla*_TEM−_, *bla*_CTX−M−,_ and *bla*_CMY−2_-producing isolates, 1 *bla*_TEM−,_ *bla*_CTX−M−,_ *bla*_OXA−10−,_ and *bla*_CMY−2−_ producing isolate, 1 *bla*_TEM−,_ *bla*_OXA−10−,_ and *bla*_CMY−2−_ producing isolates; additionally, one isolate possessed both *bla*_TEM_ and *bla*_CTX−M_.

### Detection of association between ESBL/ampC and colistin resistance gene

Colistin resistance gene (*mcr-*1) was detected in two ESBL-producing broiler chicken-originated *C. freundii* isolates (10.53%), and one ESBL-producing buffalo-originated *C. freundii* isolate (5%). These isolates were identified as *bla*_TEM,_ *bla*_CTX−M,_ and *bla*_CMY−2_ producers. Among the 19 ESBL-producing broiler chicken-originated *C. freundii* isolates, one *mcr-*1-positive isolate was identified as *bla*_TEM,_ *bla*_CTX−M,_ and *bla*_OXA−10_ producer, and one *mcr-*1-positive isolate possessed *bla*_TEM_ and *bla*_CMY−2_. An agarose profile for the identification of the colistin resistance gene was shown in Fig. 6B.

### Association between ESBL/ampC and PMQR genes

**Fig. 7:**
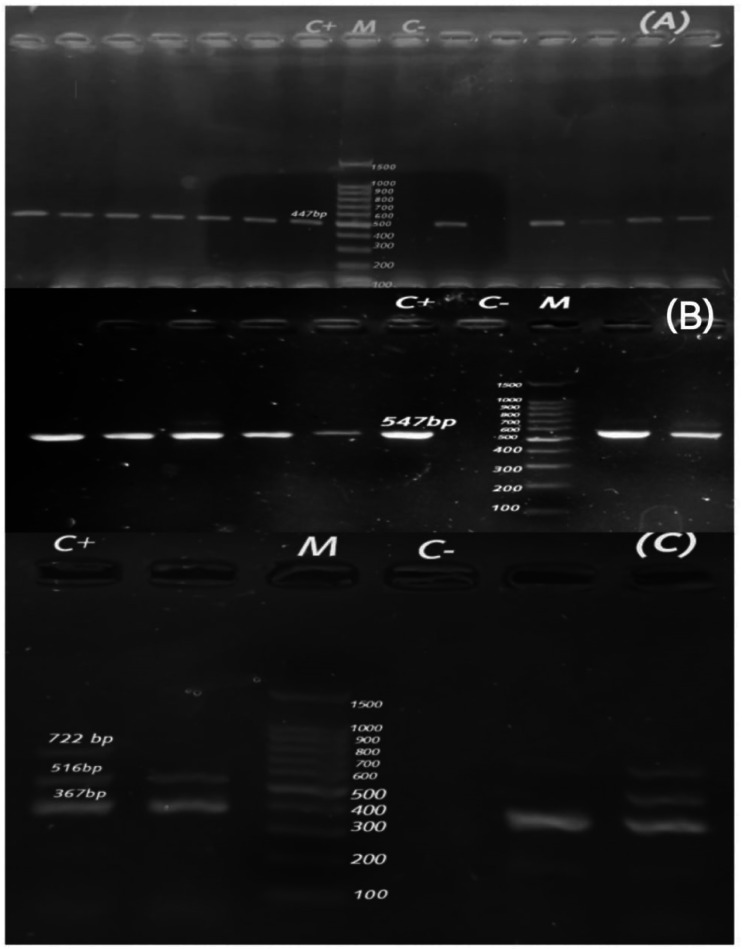
Agarose profile for the detection of resistance gene (**A**) *aad*A1 (447 bp); (**B**) *cat*A1 (547 bp); (**C**) multiplex PCR *dfr*A1 (367 bp), *qnr*A (516 bp), and *sul*2 (722 bp). Lane M: 100 bp ladder as a molecular size DNA marker. Lane C+: Control positive. C-: Control negative.

Overall, nineteen (100%) isolates of broiler chickens-derived *C. freundii* were phenotypically proven to generate PMQR, of these isolates 15 (78.95%) were resistant to levofloxacin, 17 (89.47%) were resistant to ciprofloxacin, and 19 (100%) were resistant to nalidixic acid. Meanwhile, 20 (100%) *C. freundii* originated from buffalo were phenotypically confirmed to be PMQR producers and 18 (90%) of them were resistant to nalidixic acid, 19 (95%) were resistant to levofloxacin, and 20 (100%) were resistant to ciprofloxacin. Whereas, PMQR determinant (*qnr*A) was detected in 16 broiler chicken-originated *C. freundii* isolates (84.21%) and 16 buffalo-originated isolates (80%). An important observation in our study was the discrepancy between phenotypic ciprofloxacin resistance (100% in buffalo isolates) and the detection of the plasmid-mediated *qnr*A gene (80%). An agarose profile for the identification of the *qnr*A resistance gene was shown in Fig. 7C. The frequency of *qnr*A in broiler chicken-originated *C. freundii* isolates was significant among 6 *bla*_TEM−_ and *bla*_CMY−2−_producing isolates, 4 *bla*_TEM−_ producing isolates, 2 *bla*_TEM−_ and *bla*_OXA−10−_producing isolates, 1 *bla*_TEM−,_ *bla*_CTX−M−,_ *bla*_OXA−10−,_ and *bla*_CMY−2−_producing isolate, 1 *bla*_TEM−,_ *bla*_CTX−M−,_ and *bla*_OXA−10−_producing isolate, 1 *bla*_TEM,_ *bla*_OXA−10,_ and *bla*_CMY−2−_producing isolate; also, one isolate possessed *qnr*A and produced both *bla*_TEM_ and *bla*_CTX−M_. While the prevalence of *qnr*A positivity in buffalo-originated *C. freundii* isolates was significantly observed among 5 *bla*_TEM−_producing isolates, 4 *bla*_TEM−,_ *bla*_CTX−M−,_ and *bla*_CMY−2−_producing isolates, and 4 *bla*_TEM−_ and *bla*_CMY−2−_producing isolates, 1 *bla*_TEM−,_ *bla*_CTX−M−,_ *bla*_OXA−10−,_ and *bla*_CMY−2−_producing isolate, 1 *bla*_TEM−,_ *bla*_OXA−10−,_ and *bla*_CMY−2−_producing isolate; additionally, one isolate produced *qnr*A and possessed both *bla*_TEM_ and *bla*_CTX−M_.

### Detection of association between ESBL/ampC and *dfr*A1, *aad*A1, *sul*2, *cat*A1, *tet*(M), and *erm*B encoding resistance genes

**Fig. 8:**
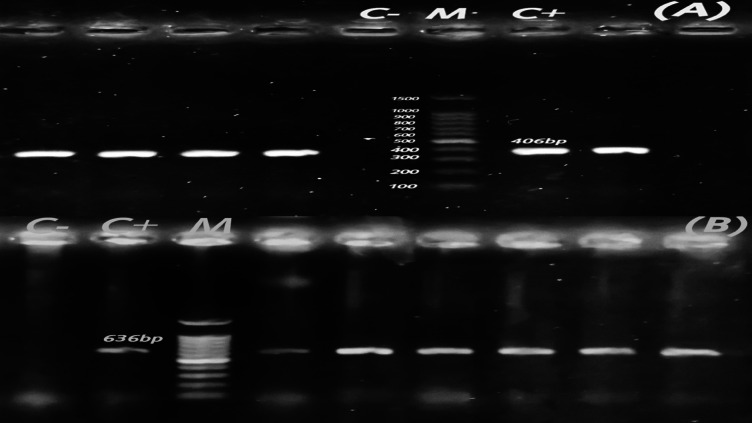
Agarose profile for the detection of resistance gene (**A**) *tet*(M) (406 bp), and (**B**) *erm*B (636 bp). Lane M: 100 bp ladder as a molecular size DNA marker. Lane C+: Control positive. C-: Control negative.

Figures 7 and 8 demonstrate an agarose profile for the identification of *dfr*A1, *aad*A1, *sul*2, *cat*A1, *tet*(M), and *erm*B encoding resistance genes. Both *dfr*A1 and *aad*A1 were prevalent (100%) in the same species. The *dfr*A1 and *aad*A1 genes were detected in broiler chicken-derived *C. freundii* isolates as follows: six isolates co-producing *bla*_TEM_ and *bla*_CMY−2_, six *bla*_TEM−_only producing isolates, two isolates co-producing *bla*_TEM_ and *bla*_OXA−10_, two co-producing *bla*_TEM_ and *bla*_CTX−M_, one isolate producing *bla*_TEM,_ *bla*_CTX−M,_ *bla*_OXA−10,_ and *bla*_CMY−2_, one producing *bla*_TEM,_ *bla*_CTX−M,_ and *bla*_OXA−10_, and one producing *bla*_TEM_ and *bla*_CTX−M_. Among buffalo-originated *C. freundii* isolates, the prevalence of *dfr*A1 and *aad*A1 was significant in 8 *bla*_TEM−_producing isolates, 4 *bla*_TEM−,_ *bla*_CTX−M−,_ and *bla*_CMY−2−_producing isolates, 4 *bla*_TEM−_ and *bla*_CMY−2−_producing isolates, 2 *bla*_TEM−_ and *bla*_CTX−M−_producing isolates, 1 *bla*_TEM−,_ *bla*_CTX−M−,_ *bla*_OXA−10−,_ and *bla*_CMY−2−_producing isolate, and 1 *bla*_TEM−,_ *bla*_OXA−10−,_ and *bla*_CMY−2−_producing isolate.

The incidence of *sul*2 was 89.47% in *C. freundii* isolates from chickens and 95% in isolates from buffalos. Additionally, the prevalence of *sul*2 in isolates originating from broiler chickens was noteworthy among the six *bla*_TEM−_ and *bla*_CMY−2−_producing isolates, four *bla*_TEM−_producing isolates, two *bla*_TEM−_ and *bla*_OXA−10−_producing isolates, two *bla*_TEM−,_ *bla*_OXA−10−,_ and *bla*_CMY−2−_producing isolates, one *bla*_TEM−,_ *bla*_CTX−M−,_ *bla*_OXA−10−,_ and *bla*_CMY−2−_producing isolate, one *bla*_TEM−,_ *bla*_CTX−M−,_ and *bla*_OXA−10−_producing isolate, and one *bla*_TEM−_ and *bla*_CTX−M−_producing isolate. In contrast, *sul*2 was significantly detected seven *C. freundii* isolates *bla*_TEM_ producing derived from buffalo (*n* = 7), *bla*_TEM_ and *bla*_CMY−2_ producers (*n* = 4), *bla*_TEM,_ *bla*_CTX−M,_ and *bla*_CMY−2_ producers (*n* = 4), *bla*_TEM_ and *bla*_CTX−M_ producers (*n* = 2), *bla*_TEM,_ *bla*_CTX−M,_ *bla*_OXA−10,_ and *bla*_CMY−2_ producer (*n* = 1), and *bla*_TEM,_ *bla*_OXA−10,_ and *bla*_CMY−2_ producer (*n* = 1).

In chicken-derived *C. freundii* isolates, the prevalence of *erm*B was 52.63%, whereas in buffalo isolates, it was 50%. Furthermore, the occurrence of *erm*B in broiler chicken-originated *C. freundii* isolates was observed in four *bla*_TEM−_producing isolates, three *bla*_TEM−_ and *bla*_CMY−2−_producing isolates, one *bla*_TEM−,_ *bla*_CTX−M−,_ *bla*_OXA−10−,_ and *bla*_CMY−2−_producing isolate, one *bla*_TEM−,_ *bla*_OXA−10−,_ and *bla*_CMY−2−_producing isolate, and one *bla*_TEM−_ and *bla*_OXA−10−_producing isolate. Although the incidence of *erm*B in buffalo-derived *C. freundii* isolates varied considerably, it was noted across 5 *bla*_TEM−_producing isolates, 2 *bla*_TEM−_ and *bla*_CTX−M−_producing isolates, 1 *bla*_TEM−,_ *bla*_OXA−10−,_ and *bla*_CMY−2−_producing isolate, 1 *bla*_TEM−,_ *bla*_CTX−M−,_ and *bla*_CMY−2−_producing isolate, and 1 *bla*_TEM−_ and *bla*_CMY−2−_producing isolate.

The prevalence of *tet*(M) was found in isolates of *C. freundii* from chickens and buffalo with percentage of 21.05% and 5%, respectively. In *C. freundii* isolates from broiler chickens, *tet*(M) was prevalent in two isolates that generated *bla*_TEM_ and one isolate that produced both *bla*_TEM_ and *bla*_CMY−2_. However, a substantial rate of *tet*(M)-positivity was observed in one *bla*_TEM−_producing strain in buffalo-originated *C. freundii* isolates.

The frequency of *cat*A1 was 5.26% in chicken-originated *C. freundii* isolates and 30% in buffalo isolates. One of the *C. freundii* isolates from broiler chickens had a high incidence of *cat*A1, which generated *bla*_TEM_ and *bla*_CMY−2_. However, *C. freundii* isolates originated buffalo, three isolates produced *bla*_TEM_, two isolates produced *bla*_TEM_ and *bla*_CMY−2_, and one isolate produced *bla*_TEM_, *bla*_CTX−M_, *bla*_OXA−10_, and *bla*_CMY−2_.

### Genotype-phenotype correlation and concordance analysis

**Fig. 9:**
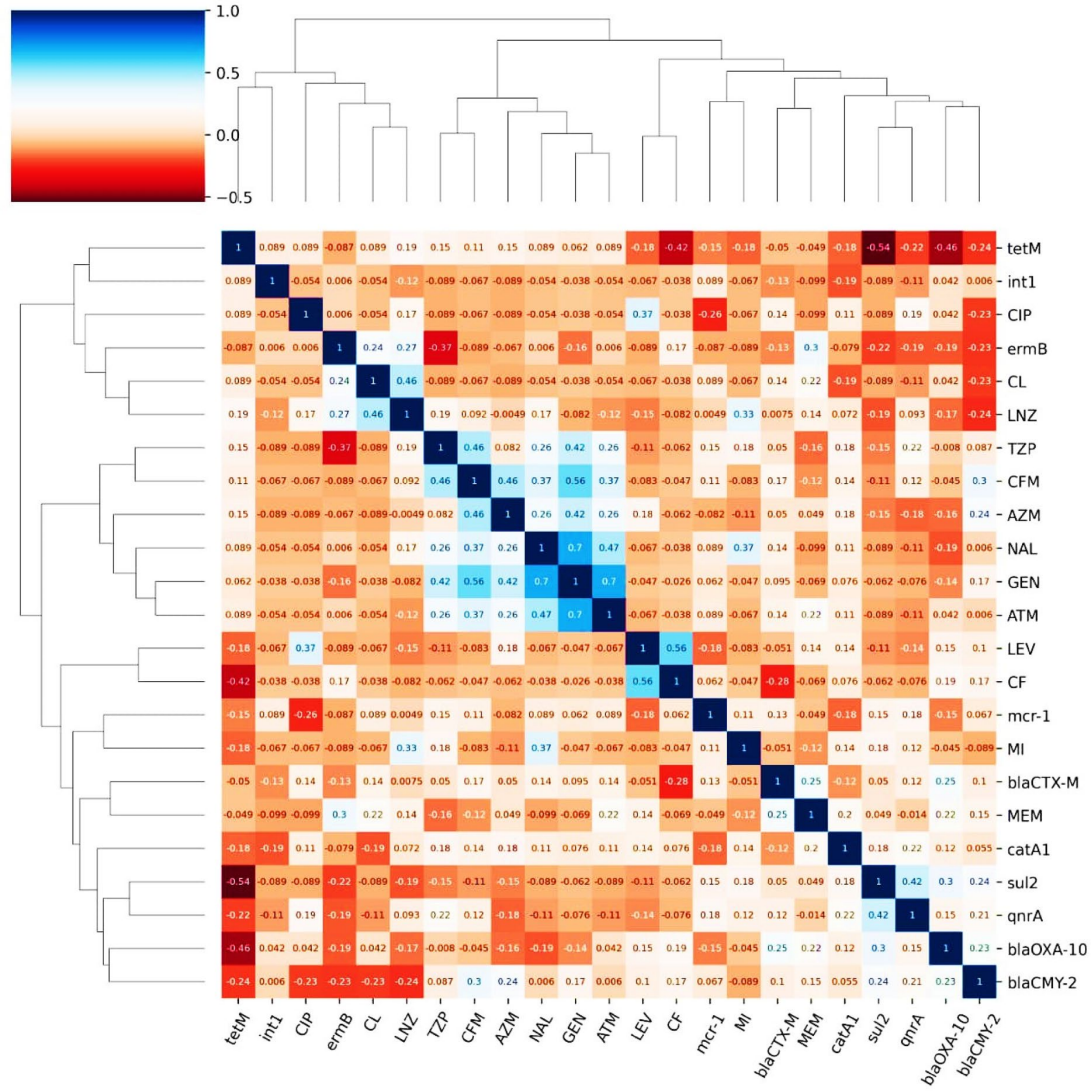
Hierarchical clustering and correlation analysis of antimicrobial resistance determinants. This comprehensive heatmap presents a correlation matrix with hierarchical clustering of antimicrobial resistance genes and corresponding phenotypic resistance profiles. Both axes display the same set of determinants, organized through unsupervised hierarchical clustering based on Pearson correlation coefficients. The resulting dendrograms reveal phylogenetic relationships and functional associations among resistance elements. Genes and antimicrobial agents that exhibited complete absence or complete presence across all isolates were excluded from the analysis, as they would yield undefined or meaningless correlation coefficients and not contribute to the clustering pattern interpretation. Distinct clustering patterns emerged, with solid-line enclosures highlighting significantly correlated gene groups that suggest potential genetic linkage or co-regulation mechanisms. The prominent cluster in the upper left quadrant groups β-lactam resistance elements with strong intercorrelations, while the *tet*(M)-*sul*2 cluster (*r* = 0.54) indicates possible plasmid-mediated co-transfer. Weakly correlated elements appear in separate clusters, reflecting independent genetic origins. Color intensity represents correlation strength ranging from + 1 (deep red) to −1 (deep blue), with white indicating no correlation. The symmetric matrix structure enables simultaneous visualization of both gene-gene and gene-antibiotic relationships within a unified analytical framework, providing insights into the complex network of antimicrobial resistance determinants.

Our comprehensive analysis of antimicrobial resistance patterns employed two complementary approaches. Initial correlation analysis of all resistance determinants (Fig. 9) revealed significant associations, including strong correlation between *tet*(M) and *sul*2 genes (*r* = 0.54) suggesting potential genetic co-transfer. The *bla*_TEM_ gene showed consistent high correlations with β-lactam antibiotics, while *qnr*A demonstrated variable associations (*r* = 0.19–0.42) with fluoroquinolone resistance. Notably, *tet*(M) exhibited weak correlations with tetracycline phenotypes (*r* = 0.087–0.24), and integron *int*1 showed limited association with most resistance markers.

Subsequent genotype-phenotype concordance analysis (Table 3) quantified these relationships across species. The *bla*_TEM_ gene achieved perfect concordance (100%) with β-lactam resistance, while *sul*2 and *dfr*A1 showed high concordance (> 89%) with trimethoprim-sulfamethoxazole resistance. In stark contrast, *tet*(M) demonstrated remarkably low predictive value (5–22.2.2%) despite high tetracycline resistance rates (90–100%). The *erm*B gene displayed moderate concordance (52.6–62.5%) with macrolide resistance, and *qnr*A showed species-dependent concordance (80–100%) with fluoroquinolones.

**Table 3:** Genotype-phenotype concordance of antimicrobial resistance genes in Buffalo and broiler chicken isolates.

Antimicrobial resistance gene	Antibiotic disc	resistant buffalo isolates No. 20	resistant broiler isolates No. 19	Gene-resistant buffalo isolates	Gene-resistant broiler chickens isolate	buffalo concordance %	broiler chickens’ concordance %
*bla*_TEM_	CFR, CPD, SAM, AX, CB, CFM, TZP, ATM,	20(100%) 20(100%) 20(100%) 20(100%) 20(100%) 18(90%) 16(80%) 19(95%) **(80–100%)**	18(94.74%) 19(100%) 19(100%) 19(100%) 19(100%) 18(94.74%) 16(84.21%) 18(94.74%) **(84.21–100%)**	(20/20) 100%	(19/19) 100%	100	100
*bla*_CTX−M_				(7/20) 35%	(3/19) 15.79%	35	15.79
*bla*_OXA−10_				(2/20) 10%	(6/19) 31.58%	10	31.58
*bla*_CMY−2_				(11/20) 55%	(9/19) 47.37%	55	47.37
*bla*_SHV_				0	0	0	0
*bla*_KPC_, *bla*_GES_, *bla*_VIM_, *bla*_IMP_, *bla*_NDM−1_, *bla*_OXA−48_	MEM	19(95%)	13(68.42%)	0	0	0	0
*sul*2	STX	20(100%)	19(100%)	(19/20) 95	(17/19) 89.47	95	89.47
*dfr*A1	STX	20(100%)	19(100%)	(20/20) 100	(19/19) 100	100	100
*qnr*A	NAL, CIP, LEV	18(90%) 20(100%) 19(95%) **(90–100%)**	19(100%) 17(89.47%) 16(84.21%) **(84.21–100%)**	(16/20)80	(16/19)84.21%	80–88.89.89	84.21–100
*erm*B	AZM, CL	17(85%) 19(95%) **(85–95%)**	16(84.21%) 18(94.74%) **(84.21–94.74%)**	(10/20) 50	(10/19) 52.63	58.82–52.63	55.56–62.5
*tet*(M)	MI, DA	18(90%) 20(100%)	18(94.74%) 19(100%)	(1/20) 5	(4/19) 21.05	5–5.6.6	21.05–22.2

Critical findings from both analyses consistently identified major discordances: universal gentamicin resistance without corresponding *aad*A1 explanatory power (which confers streptomycin resistance), high-level carbapenem resistance in the complete absence of detected carbapenemase genes, and the poor performance of *tet*(M) as a tetracycline resistance marker. These discordant patterns strongly suggest the involvement of uncharacterized resistance mechanisms, including potential *aac* genes for aminoglycoside resistance and alternative tetracycline resistance determinants beyond *tet*(M).

### Characterization of CSNPs

**Fig. 10:**
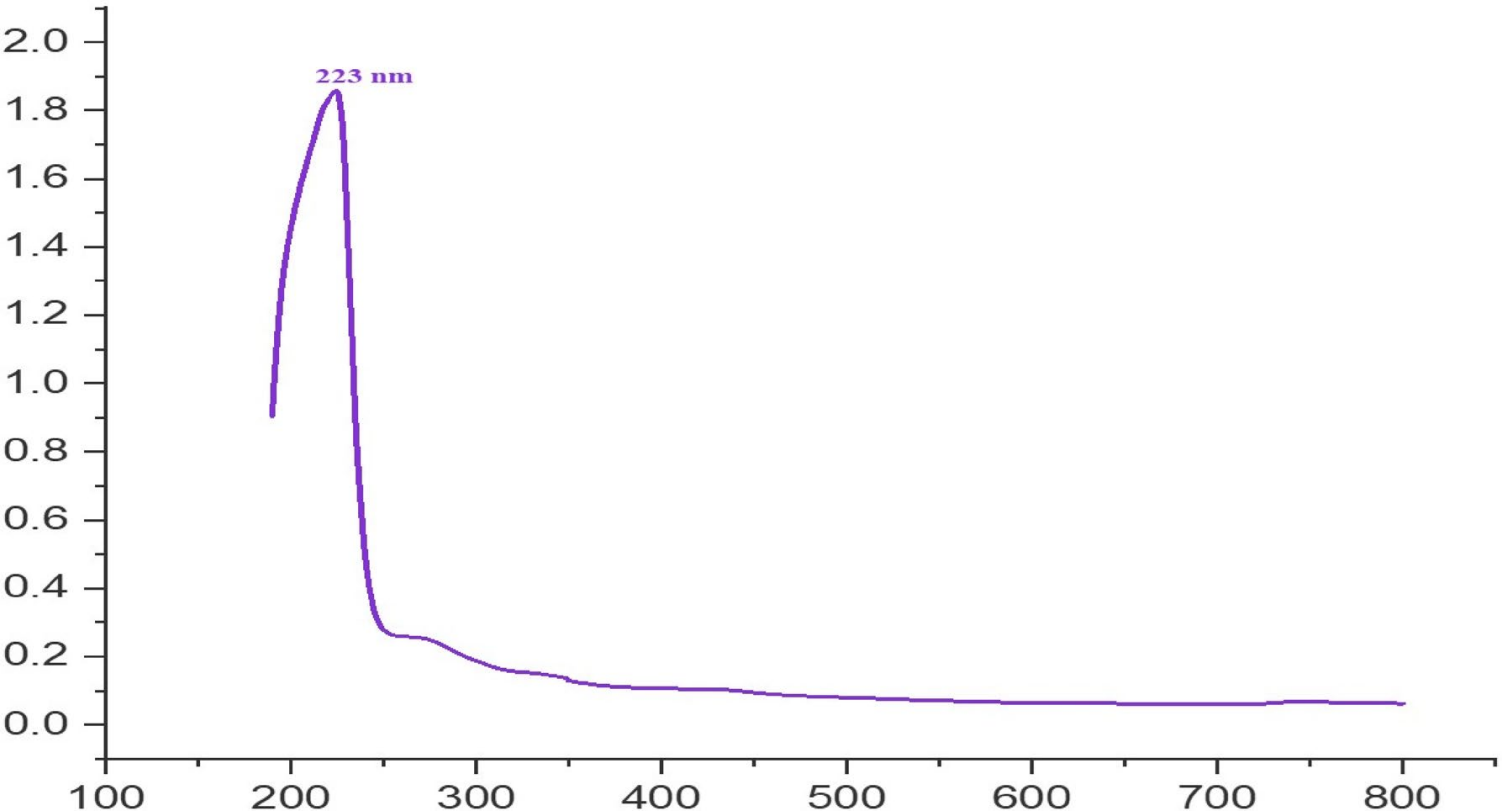
UV-Vis analysis of the synthesized CSNPs.

**Fig. 11:**
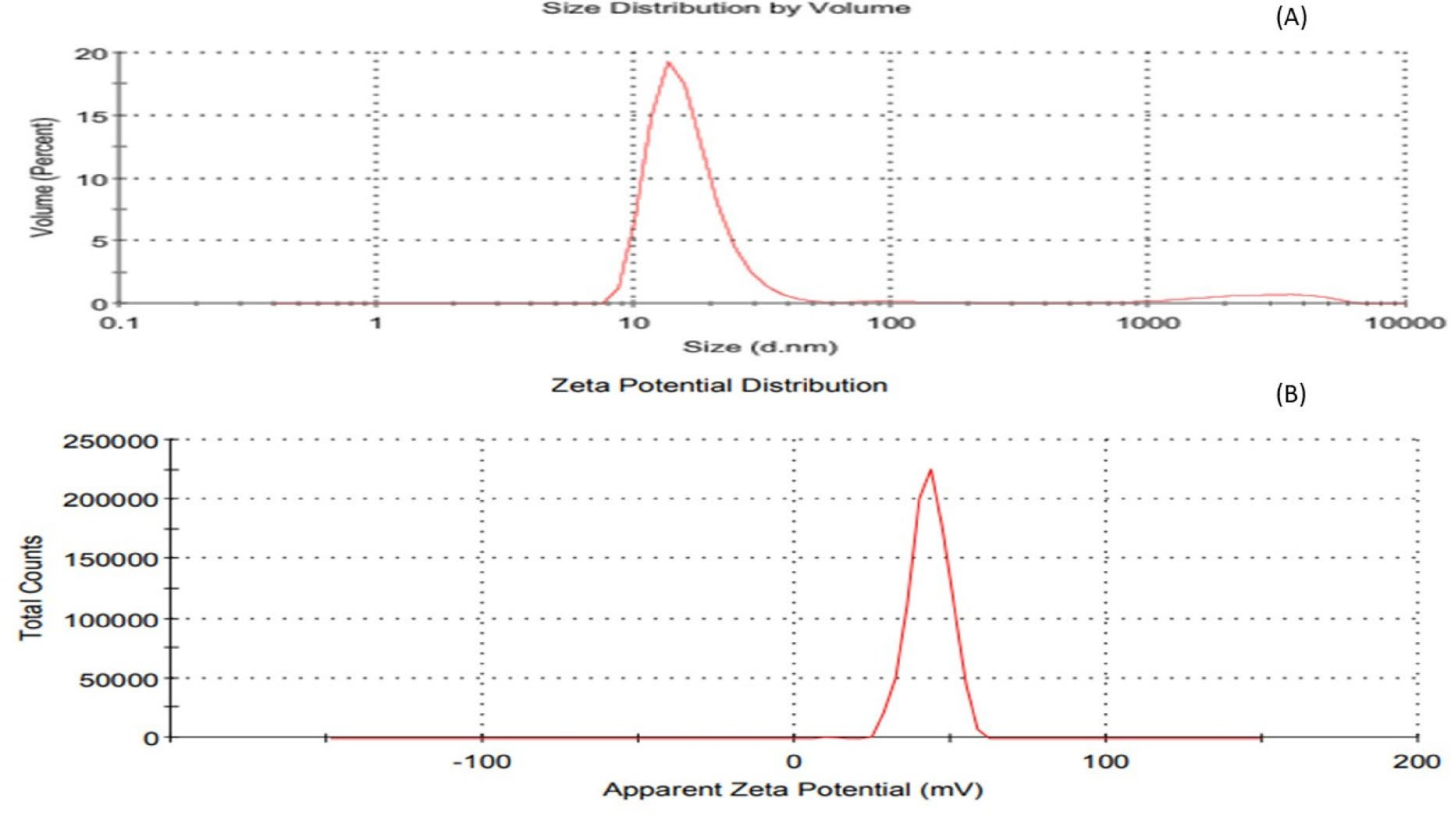
Size distribution (**A**), and Zeta potential of the synthesized CSNPs (**B**).

In the current study, a wide absorption band peak at 223 nm was noted (Fig. 10), showing the existence of freshly created CSNPs in the process besides nanoparticles transitioning from a grounded to an excited state. This reveals the existence of nanoparticle surface plasmon resonance (SPR), with a single SPR band indicating that the nanoparticles are spherical. Utilizing PSA evaluations, the average hydrodynamic diameter of CSNPs was calculated at the maximum intensity of 194.8 nm (Fig. 11A). This size of CSNPs shows that nanoparticles were produced. This size is significantly bigger than that expected by electron microscopy due to the remarkable swelling capacity of chitosan nanoparticles.

A very stable dispersion is shown by the synthesized CSNPs’ mean zeta potential of 43 mV with a zeta deviation of 6.44 mV at 25 °C and 1.14 mS/cm conductivity. The low zeta deviation reflects the minimum change in particle surface charge among the studied samples (Fig. 11B).

**Fig. 12:**
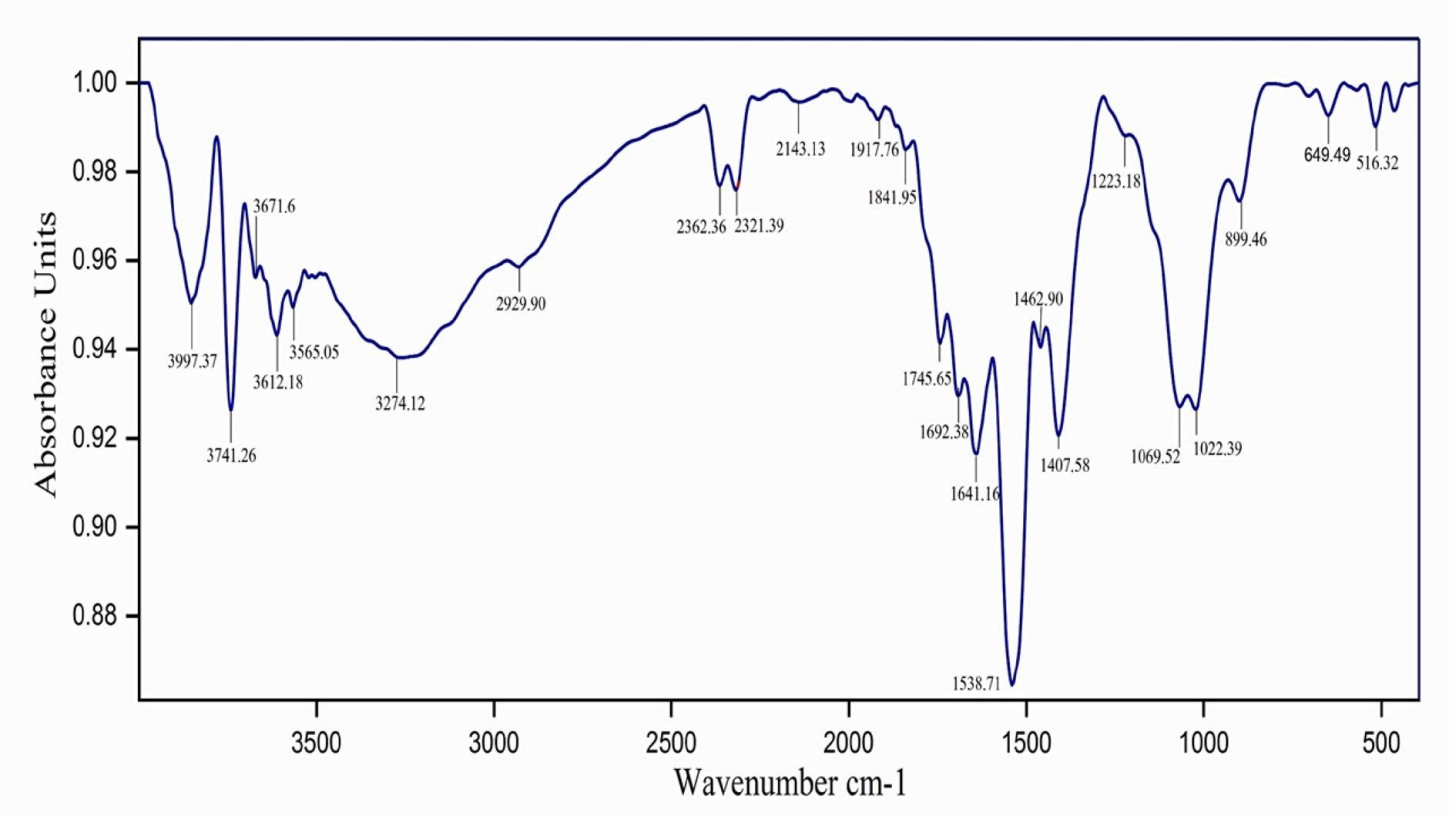
The FTIR spectrum of CSNPs.

The FTIR spectrum of chitosan-TPP nanoparticles shows several unique peaks, each corresponding to a specific functional group, indicating the nanoparticles’ successful production by ionic gelation. The FTIR spectra of chitosan is depicted in Fig. 12. The peaks at 3997.37–3612.18 cm⁻¹ indicated strong hydrogen bonding and the presence of hydroxyl and amine groups in chitosan. The signal at 2929.90 cm⁻¹ was created by C-H stretching from aliphatic -CH₂ groups, which were typical of the chitosan backbone. Minor peaks at 2362.36 and 2143.13 cm⁻¹ might be due to absorbed ambient CO₂ or leftover reagents. Peaks at 1841.95 and 1745.65 cm⁻¹ corresponded to carbonyl (C = O) stretching vibrations, which were often associated with amide I bands and indicated TPP-chitosan interactions. Peaks at 1692.38, 1641.16, and 1538.71 cm⁻¹ were attributed to amide I and amide II bands, respectively, showing C = O stretching and N-H bending vibrations, which enhance the creation of polyelectrolyte complexes. Peaks at 1462.90 and 1407.58 cm⁻¹ showed the existence of chitosan by representing -CH₂ bending and O-H deformation vibrations. A prominent and abrupt peak at 1223.18 cm⁻¹ indicates P = O stretching, which was linked to the TPP crosslinker. The band at 1069.52 cm⁻¹ was attributed to P-O-C stretching, which supported the integration of TPP into the chitosan matrix. Peaks at 1022.39 and 899.46 cm⁻¹ corresponded to C-O-C and C-O-H vibrations seen in polysaccharides, including chitosan. The bands at 649.49 and 516.32 cm⁻¹ showed P-O bending vibrations, indicating the presence of phosphate and successful ionic interaction with TPP. Finally, the FTIR spectra showed functional group interactions between chitosan and TPP, demonstrating the successful synthesis of chitosan-TPP nanoparticles by ionic gelation.

**Fig. 13:**
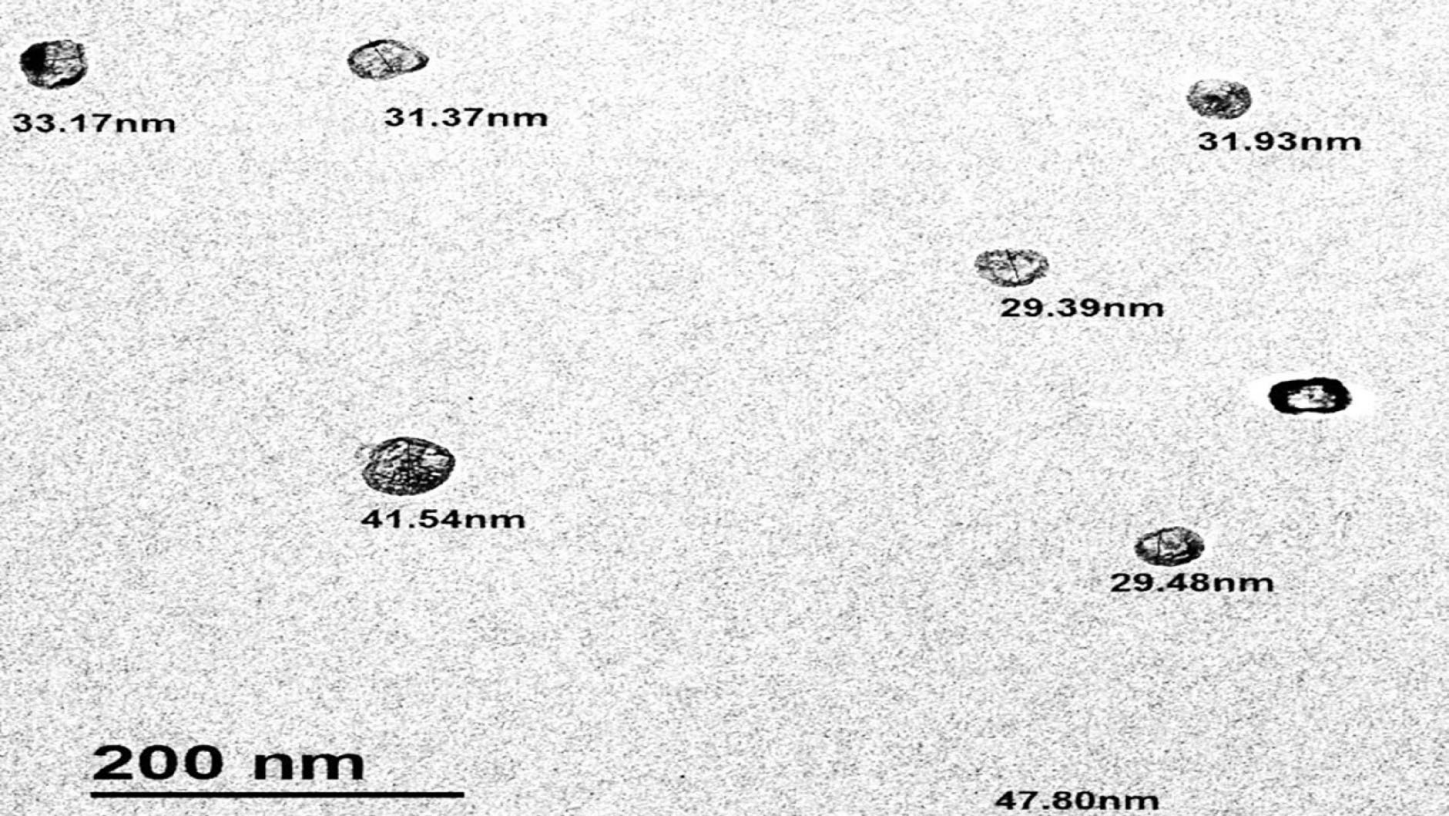
TEM image of CSNPs.

The surface appearance and size distribution of CSNPs were examined using TEM. With a mean diameter of 34.95 ± 6.49 nm, the TEM picture displayed a small, homogeneous appearance, smooth surface, and basically spherical shape. A manual method of measuring particle diameter (length) was used on the particles listed in Fig. 13 after the TEM image’s scale was adjusted to the measured scale-bar value.

### Antibacterial efficacy of CS and CSNPs against *C. freundii*

The sensitivity of 19 tested isolates from broiler chickens and 20 tested isolates from buffalo was determined in vitro using the agar well diffusion test. The inhibition zone diameter (*P* < 0.05) for CIP was 1.69 ± 0.47 and 1.57 ± 0.36 mm (*P* < 0.001), 1.91 ± 0.19 and 1.69 ± 0.35 mm (*P* < 0.001) for CS 1%, (*P* = 0.006) 2.20 ± 0.24 and (*P* = 0.03) 1.96 ± 0.35 mm for CS 2%, and 2.33 ± 0.24 and 2.09 ± 0.36 mm (*P* < 0.001) for CSNPs (Table 4) (Supplementary file 1). The sensitivity of isolates obtained from buffalo and broiler chickens to the enhanced inhibition zone of antibacterial agents with CIP was also demonstrated in Table 4, where the inhibition zone diameter was 2.35 ± 0.23 and 2.06 ± 0.27 mm for CIP with CS 1%, 2.63 ± 0.28 and 2.31 ± 0.32 mm for CIP with CS 2%, and 2.75 ± 0.34 and 2.44 ± 0.32 mm for CIP with CSNPs (*P* < 0.001). In broiler chickens and buffalo, the combined antibacterial activity of CS 1%, CS 2%, and CSNPs was 1.66 and 1.75; 2.09 and 2.2; and 2.33 and 2.43 times more than that of CIP (Table 5) (Supplementary file 1).

**Table 4:** Antibacterial activity of CS, CSNPs, and CIP, alone and in combination, against resistant *C. freundii* isolates from broiler chickens and Buffalo p-values from independent T test; *p* < 0.05.

**Antimicrobial agents**	**broiler N (19)**	**Buffalo N (20)**	**P value T test**
CS 10 mg/ml	1.91±0.19	1.69±0.354	0.024*
CS 20 mg/ml	2.2±0.243	1.96±0.353	0.019*
CSNPs 0.25 mg/ml	2.33±0.243	2.09±0.364	0.02*
CIP 5μg/ml	1.69±0.47	1.57±0.365	0.4
CS 10 mg/ml + CIP 5μg/ml	2.35±0.232	2.06±0.27	0.001*
CS 20 mg/ml + CIP 5μg/ml	2.63±0.283	2.31± 0.325	0.003*
CSNPs 0.25 mg/ml + CIP 5μg/ml	2.75±0.345	2.44± 0.325	0.007*

Where, * refer to statistically significant difference at p<0.05.

**Table 5:** Enhanced antibacterial activity of CIP by CS formulations compared by individual agents. Fold change (Area) = Inhibition zone area of treatmen/Inhibition zone area of CIP (control). p-values are from post hoc bonferroni comparisons between each treatment group and the CIP control.

**Agents**	**Buffalo**				**Broiler chickens**			
	**Mean Zone Diameter (mm) ±SD **	**Zone Area= (πr²) (mm²)**	**Fold-change (Area)**	**P value **	**Mean Zone Diameter (mm) ±SD **	**Zone Area= (πr²) (mm²)**	**Fold-change (Area)**	**P value **
**CIP (control)**	1.57±0.36	2.05	1		1.69±0.47	2.40	1	
**CS 1%**	1.69±0.35	2.34	1.14	1	1.91±0.19	2.88	1.20	1
**CS 2%**	1.96±0.35	3.11	1.52	0.02*	2.2±0.24	3.84	1.60	0.006*
**CSNPs**	2.09±0.36	3.53	1.72	<0.001*	2.33±0.24	4.31	1.80	<0.001*
**CS 1% + CIP**	2.06±0.27	3.40	1.66	<0.001*	2.35±0.23	4.38	1.83	<0.001*
**CS 2% + CIP**	2.31±0.32	4.29	2.09	<0.001*	2.63±0.28	5.50	2.29	<0.001*
**CSNPs + CIP **	2.44±0.32	4.77	2.33	<0.001*	2.75±0.34	6.04	2.52	<0.001*

Where, * refer to statistically significant difference at p<0.05.

The assessment of potential enhancement between CIP and chitosan-based agents was based on a quantitative analysis of the zone of inhibition diameters and the derived fold-change in the inhibition area. The results, summarized in Table 5, demonstrate that CIP alone at 5 µg/mL exhibited the weakest antibacterial activity against the tested isolates, indicating its limited efficacy as a standalone agent at this concentration. In contrast, a clear, dose-dependent increase in the zone diameter was observed for the chitosan agents alone (CS 1% < CS 2% < CSNPs), establishing a foundational dose-response relationship. The critical finding emerged from the combination assays. All combinations of CIP with CS 1%, CS 2%, or CSNPs produced a substantial and statistically significant (*p* < 0.001) enhancement in the antibacterial effect (Table 5). This was most evident in the calculated fold-change of the inhibition area. The combination of CSNPs with CIP resulted in a 2.52-fold and 2.33-fold increase in the inhibition area for broiler and buffalo isolates, respectively, compared to CIP alone. Similarly, combinations with CS 2% + CIP and CS 1% + CIP also yielded fold-increases exceeding 2.0 and 1.66, respectively. Given that the individual components—a weakly inhibitory CIP concentration and sub-optimal concentrations of chitosan agents—produced only modest effects, this more than doubling of the antibacterial effect in combination provides compelling evidence of an enhancement interaction, surpassing a merely additive outcome. As shown in Table 6, the post hoc analysis using Bonferroni correction revealed that the combination of CSNPs with CIP demonstrated significantly superior antibacterial efficacy against all bacterial isolates compared to other treatment, indicating an enhanced antibacterial effect.

**Table 6:** Post hoc pairwise comparisons of antibacterial efficacy. Post hoc analysis was performed following repeated measures ANOVA using bonferroni correction. *p* < 0.05 indicates a statistically significant difference.

Post hoc comparison of inhibition zone measurements against bacterial isolates on both sample type		**broiler isolates**		**buffalo isolates**	
		**Mean Difference**	**P value Bonferroni**	**Mean Difference**	**P value Bonferroni**
CSNPs 0.25 mg ml + CIP 5μg/ml	CS 20 mg/ml + CIP 5μg/ml	0.12	0.06	0.13	0.07
CSNPs 0.25 mg ml + CIP 5μg/ml	CS 10 mg/ml + CIP 5μg/ml	0.40	< 0.001*	0.38	< 0.001*
CSNPs 0.25 mg ml + CIP 5μg/ml	CIP 5μg/ml	1.06	< 0.001*	0.87	< 0.001*
CSNPs 0.25 mg ml + CIP 5μg/ml	CSNPs 0.25 mg ml	0.42	< 0.001*	0.36	< 0.001*
CSNPs 0.25 mg ml + CIP 5μg/ml	CS 20 mg/ml	0.55	< 0.001*	0.49	< 0.001*
CSNPs 0.25 mg ml + CIP 5μg/ml	CS 10 mg/ml	0.85	< 0.001*	0.76	< 0.001*
CS 20 mg/ml + CIP 5μg/ml	CS 10 mg/ml + CIP 5μg/ml	0.28	< 0.001*	0.25	< 0.001*
CS 20 mg/ml + CIP 5μg/ml	CIP 5μg/ml	0.94	< 0.001*	0.74	< 0.001*
CS 20 mg/ml + CIP 5μg/ml	CSNPs 0.25 mg ml	0.30	< 0.001*	0.23	0.08
CS 20 mg/ml + CIP 5μg/ml	CS 20 mg/ml	0.43	< 0.001*	0.36	< 0.001*
CS 20 mg/ml + CIP 5μg/ml	CS 10 mg/ml	0.73	< 0.001*	0.63	< 0.001*
CS 20 mg/ml	CIP 5μg/ml	0.51	0.006*	0.39	0.02*
CS 10 mg/ml + CIP 5μg/ml	CIP 5μg/ml	0.66	< .001*	0.49	< 0.001*
CS 10 mg/ml + CIP 5μg/ml	CSNPs 0.25 mg/ml	0.02	1.00	-0.03	1.00
CS 10 mg/ml + CIP 5μg/ml	CS 20 mg/ml	0.15	0.053*	0.11	1.00
CS 10 mg/ml + CIP 5μg/ml	CS 10 mg/ml	0.45	< 0.001	0.38	< 0.001*
CS 10 mg/ml	CIP 5μg/ml	0.22	1.00	0.12	1.00
CSNPs 0.25 mg ml	CS 20 mg/ml	0.13	0.007*	0.13	0.03*
CSNPs 0.25 mg ml	CS 10 mg/ml	0.43	< 0.001*	0.40	< 0.001*
CSNPs 0.25 mg/ ml	CIP 5μg/ml	0.64	< 0.001*	0.52	0.001*
CS 20 mg/ml	CS 10 mg/ml	0.29	< 0.001*	0.27	< 0.001*
CS 20 mg/ml	CIP 5μg/ml	0.51	0.006*	0.39	0.02*

Where, * refer to statistically significant difference at p<0.05

### Determination of MIC and MBC of CS 1%, CS 2%, and CSNPs against resistant *C. freundii*

The median MIC values of CS 1%, CS 2%, and CSNPs against *C. freundii* isolates from broilers were 4.17, 3.33, and 0.0833 mg/mL, respectively. In buffalo isolates, the corresponding median MIC values were 3.33, 2.92, and 0.125 mg/mL, respectively (details shown in Table 7) (supplementary file 2). To assess the effects of CSNPs, CS 2%, and CS 1% on the MIC of broiler bacterial isolates, a Friedman test was performed. The test indicated a statistically significant difference, χ²(2) = 29.2, *p* < 0.001. Post-hoc pairwise comparisons using the Durbin–Conover test showed that CSNPs differed significantly from both CS 2% and CS 1% (*p* < 0.001), whereas no significant difference was observed between CS 2% and CS 1% (*p* = 0.110).

**Table 7:** MIC and MBC values of CS 1%, CS 2%, and CSNPs against resistant *C. freundii* isolates collected from broiler chickens and buffalo

**MIC ** **results**	**broiler N (19)**		**buffalo N (20)**		**P value**	**MBC/MIC Ratio**		
	**Median**	**IQR**	**Median**	**IQR**		**Broiler**	**Buffalo**	
CSNPs MIC	0.0833	0.0495	0.125	0.0755	0.06	2	1.25	Bactericidal effect
CSNPs MBC	0.167	0.0573	0.156	0.120	0.46			
CS 2% MIC	3.33	2.34	2.92	1.69	0.58	1.5	1.37	
CS 2% MBC	5.00	2.04	4	0.83	0.001*			
CS 1% MIC	4.17	5.73	3.33	0.859	0.30	1.4	1.25	
CS 1% MBC	5.83	2.92	4.17	1.67	0.164			

Where, * refer to statistically significant difference at p<0.05.

For buffalo bacterial isolates, a Friedman test also showed a statistically significant difference, χ² (2) = 31.6, *p* < 0.001. Post-hoc comparisons showed that CSNPs differed significantly from both CS 2% and CS 1% (*p* < 0.001), whereas no significant difference was observed between CS 2% and CS 1% (*p* = 0.111). A Mann–Whitney U test was conducted to compare the MIC values between broiler and buffalo isolates for each treatment. The test showed no statistically significant differences between the two animal groups for the three drugs (all *p* > 0.05) (Table 8).

**Table 8:** Statistical comparison (Durbin-Conover) of antimicrobial efficacy (MIC and MBC) between CSNPs, CS 1%, and CS 2% in broiler chickens and Buffalo isolates.

**Pairwise comparisons**		**P **value
MIC in broiler chickens		
CSNPs MIC	CS 2% MIC	< .001*
CSNPs MIC	CS 1% MIC	< .001*
CS 2% MIC	CS 1% MIC	0.11
MIC in buffalo		
CSNPs MIC	CS 2% MIC	< .001*
CSNPs MIC	CS 1% Mic	< .001*
CS 2% MIC	CS 1% Mic	0.011*
MBC in broiler chickens		
CSNPs MBC	CS 2% MBC	< .001*
CSNPs MBC	CS 1% MBC	< .001*
CS 2% MBC	CS 1% MBC	0.51
MBC in buffalo		
CSNPs MIC	CS 2% MIC	< .001*
CSNPs MIC	CS 1% MIC	< .001*
CS 2% MIC	CS 1% MIC	0.11

P value of pairwise comparisons (Durbin-Conover)

The median MBC values of CS 1%, CS 2%, and CSNPs against broiler isolates were 5.00, 5.83, and 0.167 mg/mL, respectively. In buffalo isolates, the corresponding median MBC values were 4.17, 4.00, and 0.156 mg/mL, respectively (details shown in Table 7) (Supplementary file 2). To assess the effects of CSNPs, CS 2%, and CS 1% on the MBC of broiler bacterial isolates, a Friedman test was performed. The test indicated a statistically significant difference, χ²(2) = 29.0, *p* < 0.001. Post-hoc pairwise comparisons using the Durbin–Conover test showed that CSNPs differed significantly from both CS 2% and CS 1% (*p* < 0.001), whereas no significant difference was observed between CS 2% and CS 1% (*p* = 0.518).

For buffalo bacterial isolates, a Friedman test also showed a statistically significant difference, χ² (2) = 31.2, *p* < 0.001. Post-hoc comparisons showed that CSNPs differed significantly from both CS 2% and CS 1% (*p* < 0.001), whereas no significant difference was observed between CS 2% and CS 1% (*p* = 0.110). A Mann–Whitney U test was conducted to compare the MBC values between broiler and buffalo isolates for each treatment. The results showed that broiler isolates had significantly higher MBC values for CS 2% compared with buffalo isolates (*p* = 0.001). However, no significant differences were observed between the two animal groups for CSNPs and CS 1% (both *p* > 0.05) (Table 8). Across all treatments, the MBC/MIC index was ≤ 2 for CS 1%, CS 2%, and CSNPs in both broiler and buffalo isolates (details in Table 7). This indicates that all three preparations exhibited bactericidal rather than bacteriostatic effects against *C. freundii*. The consistently lowest MIC and MBC values observed for CSNPs further reinforce its superior and stable bactericidal activity compared with conventional chitosan formulations.

**Fig. 14:**
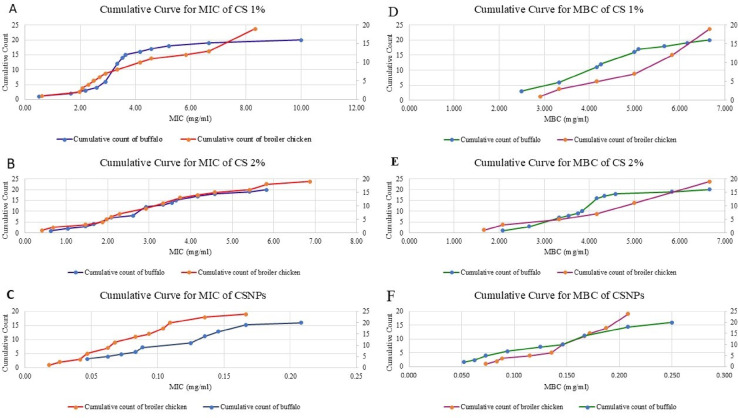
Cumulative curves of MIC and MBC.

The comparative analysis of antimicrobial efficacy revealed the consistent and remarkable superiority of CSNPs over CS. As presented in Table 9; Fig. 14, CSNPs exhibited drastically lower MIC₉₀ values of 0.17 mg/ml and 0.14 mg/ml against buffalo and broiler chicken isolates, respectively. This potent inhibitory activity was paralleled by bactericidal effects, with MBC₉₀ values of 0.208 mg/ml for isolates from both sources. In sharp contrast, CS at both 1% and 2% concentrations demonstrated substantially higher MIC₉₀ of buffalo (5.21–4.58 mg/ml), MIC₉₀ of broiler chicken (8.33–5.83 mg/ml) and MBC₉₀ of buffalo (5.667–4.583 mg/ml), MBC₉₀ of broiler chicken (6.667–6.667 mg/ml) values. The close convergence of MIC and MBC values for CSNPs indicates a primarily bactericidal mode of action. Furthermore, isolates from buffalo showed consistently higher susceptibility across all agents compared to broiler chicken isolates.

### Scanning electron microscopy of *C. freundii*

**Fig. 15:**
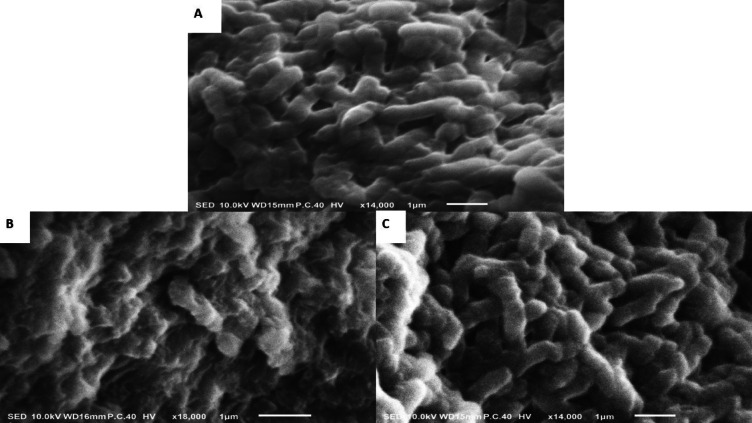
SEM micrographs of *C. freundii* cytomorphology before (control) (**A**) and after treatment with 0.156 mg/ml of CS (**B**) and with 0.0078 mg/ml CSNPs (**C**) after 18 h incubation.

With respect to the bactericidal action of the CS and CSNPs, the shape of the tested bacterial cells was discriminated from a control group after supplying the bacterial cells with 0.156 mg/mL of CS and 0.0078 mg/mL of CSNPs for 18 h of incubation using SEM. Untreated control cells (negative control) exhibited typical rod-shaped cytomorphology with smooth, well-defined surfaces (Fig. 15A) (Supplementary file 3**A)**. In contrast, cells treated with CSNPs at 0.0078 mg/mL displayed pronounced morphological alterations, including surface dents, perforations, and collapsed structures. Also, most cells appeared wrinkled, shrunken, or severely degraded (Fig. 15B) (Supplementary file 3**B**). Treatment with CS at 0.156 mg/mL caused partial cell damage: some cells exhibited distortion, enlargement, and irregular surfaces, while others retained smooth morphology and intact structures (Fig. 15C) (Supplementary file 3**C**). These morphological observations were reproducible across multiple examined fields of view (≥ 2–4 per sample), with several micrographs obtained per condition. Additional supporting micrographs for each condition have been provided as Supplementary Material (Supplementary files 3**A**, 3**B**, 3**C**). The presented figures were selected as representative of the predominant features observed. Although the findings are primarily qualitative, the frequency of deformed cells was consistently higher in CSNP-treated populations compared with CS-treated ones. Overall, these results suggest that CS exhibits a concentration-dependent antibacterial effect, while CSNPs, owing to their smaller size, larger surface area, and stronger affinity for bacterial membranes, exert stronger destructive effects even at lower concentrations. This supports the superior antibacterial efficiency of CSNPs compared with CS.

## Discussion

### Prevalence of *C. freundii* among *Citrobacter* spp

*Citrobacter* spp. were isolated from diseased broiler chickens and native Egyptian buffalo at rates of 10.48% (33/315) and 16.89% (38/225) of the total 540 samples, respectively. Molecular screening indicated that *C. freundii* comprised a higher proportion of isolates from broiler chickens (57.58%, 19/33) compared to buffalo (52.63%, 20/38). These prevalence rates are higher than those reported by Liu et al.^6^, who detected *C. freundii* in 26.19% (11/42) in samples from foods and food handlers, and Witaningrum et al.^46^, who identified *C. freundii* in 13.75% of broiler chicken farms. The observed variation in prevalence across studies may reflect differences in sample sources, types, and environmental factors, including hygiene practices, farm management, overcrowding, and ventilation. Such conditions likely create environments conducive to bacterial colonization and proliferation, highlighting the influence of farm-level practices on the epidemiology of *C. freundii* in food animals.

### Profiling of phenotypic antimicrobial resistance of *C. freundii* isolates

*C. freundii* produces ESBLs and harbors chromosomal ampC β-lactamases, limiting the available antimicrobial options and raising public health and clinical concerns^47^. Previously regarded as non-classical pathogens, *C. freundii* are increasingly recognized as clinical multidrug-resistant pathogens due to their remarkable capacity to acquire diverse resistance mechanisms^48^. The current investigation revealed that all broiler chicken-derived *C. freundii* isolates were completely resistant to critically important antimicrobials comprising gentamicin, nalidixic acid, ampicillin/sulbactam, amoxicillin, carbenicillin, and cefpodoxime, alongside highly important antimicrobials involving clindamycin and sulfamethoxazole/trimethoprim. Furthermore, 94.74% of the chicken isolates were resistant to critically important antimicrobials that involve clarithromycin, aztreonam, and cefixime, alongside highly important antimicrobials including cefadroxil and minocycline. Moreover, 89.47%, 84.21%, 84.21%, 78.95%, 73.68%, and 68.42% of the isolates were resistant to critically important antimicrobials, including ciprofloxacin, azithromycin, piperacillin/tazobactam, levofloxacin, linezolid, and meropenem, respectively. Overall, complete resistance was observed in buffalo-originated *C. freundii* isolates against critically important antimicrobials like ampicillin/sulbactam, amoxicillin, carbenicillin, ciprofloxacin, and cefpodoxime and highly important antimicrobials like clindamycin, sulfamethoxazole/trimethoprim, and cefadroxil (100% each). Critically important antimicrobials, including clarithromycin, gentamicin, meropenem, aztreonam, and levofloxacin, were also shown to have an extremely high resistance rate of 95%. Furthermore, resistance rate of 90% was found to highly important antimicrobials, including minocycline, and also critically important antimicrobials like cefixime and nalidixic acid. However, resistance to the three critically important antimicrobials linezolid, azithromycin, and piperacillin/tazobactam, was identified to be 85%, 85%, and 80%, respectively. These results disagree with Ejaz et al.^14^, who reported that *C. freundii* expressed resistance to aztreonam with a rate of 71.4%, cefpodoxime (100%), gentamicin (71.4%), ciprofloxacin (28.5%), meropenem (0%), and piperacillin/tazobactam (0%). While Liu et al.^6^ reported that *C. freundii*isolates from food showed resistance to aztreonam, meropenem, ciprofloxacin, levofloxacin, gentamicin, streptomycin, tetracycline, trimethoprim/sulfamethoxazole, and azithromycin with rates of 0%, 0%, 18.18%, 27.27%. Witaningrum et al.^46^. studied *C. freundii*taken from healthy broiler chickens and found it was resistant to ampicillin (77.27%), tetracycline (59.09%), trimethoprim/sulfamethoxazole (50%), and streptomycin (22.73%). Also, Zhang et al.^49^. reported 33.3%, 45.8%, 54.2%, 12.5%, and 58.3% resistance against aztreonam, meropenem, piperacillin-tazobactam, gentamicin, and levofloxacin, respectively. In comparison, Liu et al.^50^. exhibited resistance rates of *C. freundii* to aztreonam (19.2%), meropenem (3.8%), nalidixic acid (30.8%), ciprofloxacin (11.5%), levofloxacin (11.5%), gentamicin (15.4%), streptomycin (26.9%), tetracycline (19.2%), trimethoprim/sulfamethoxazole (30.8%), and azithromycin (65.4%). These extremely high resistance rates, including complete resistance to more than ten antimicrobial classes, are notably higher than those reported in most previous studies, possibly reflecting intensive and often uncontrolled antimicrobial use in food-producing animals in the studied region, strong selective pressures favoring MDR strains, clonal expansion of highly resistant lineages, and horizontal gene transfer via plasmids, transposons, or integrons. These factors together may account for the extreme resistance profile reported here and underline the urgent need for molecular epidemiological investigations to differentiate between clonal spread and gene transfer–driven resistance.

Interestingly, 100% (19/19) of the *C. freundii* isolates from broiler chickens exhibited beta-lactamase characteristics based on their antibiotic resistance profiles. Broiler chicken isolates exhibited MAR values ranging from 0.692 to 1, while buffalo isolates ranged from 0.538 to 1. Within our study sample, buffalo isolates exhibited higher PDR-like profiles and maximum MAR indices (1.0) more frequently than broiler isolates, highlighting the overuse of antibiotics and the elevated resistance level in Egyptian buffalo farms. Importantly, the maximum MAR index (1.0) was exclusively associated with these PDR-like isolates, occurring more frequently in buffalo than in broiler chickens. Taken together, these results highlight buffalo-derived isolates as exhibiting a comparatively higher level of resistance, although this interpretation should be made cautiously due to the restricted sample size. These findings underscore the high prevalence of extreme resistance in food-animal *C. freundii*populations and emphasize the urgent need for broader surveillance using comprehensive antimicrobial panels to mitigate the dissemination of multidrug-resistant strains. Liu et al.^50^. reported a MDR prevalence of 65.4%. Liu et al.^16^. found that 30.6% of isolates were resistant to at least three of the ten antibiotic classes investigated. Witaningrum et al.^46^. found that 81.82% of *C. freundii* had an MDR profile in broiler chicken farms. This high incidence of MDR and PDR-like resistance in foodborne pathogens is reason for worry, particularly given the likelihood of cross-contamination across the food chain, which poses a danger to human and animal health. A MAR score higher than 0.2 indicates misuse and abuse of antimicrobial agents in veterinary farms^51^. It’s interesting to note that every single isolate of *C. freundii* has a MAR value greater than 0.2. The antibiograms generated by the current investigation are different from those of earlier investigations, showing how the antibiotic pattern varies based on the isolate, time, XDR/PDR generation within *C. freundii* isolates, the presence of beta-lactamase-producer, and the abilities of analyzing laboratories.

### Detection of ESBL, ampC, and carbapenem encoding genes

The emergence of ESBLs in chromosomal ampC-producing *Enterobacteriaceae* is a growing concern with significant clinical and epidemiological implications, observed worldwide^52^. Namikawa et al.^53^ reported that the case mortality rate for *Enterobacterales* infections producing ESBLs ranges from 12% to 41%. Interestingly, the global incidence of ESBL in *Citrobacter* spp. is 0.5–36%^54^. The primary cause of nosocomial illness outbreaks and treatment failures are now germs that produce ampC β-lactamase^55^. In broiler chicken-derived *C. freundii* isolates, the prevalence of resistance genes was 100% *bla*_TEM_, 31.58% *bla*_OXA−10_, 15.79% *bla*_CTX−M_, 0% *bla*_SHV_, and 47.37% ampC (*bla*_CMY−2_), whereas in buffalo-derived isolates, these were 100%, 10%, 35%, 0%, and 55%, respectively. Unlike the findings of this investigation, Hassan & Shobrak^56^ discovered just one incidence of *bla*_TEM_ and did not test *bla*_OXA_, *bla*_CTX−M_, *bla*_SHV_, or ampC (*bla*_CMY−2_) in *C. freundii*isolates from wild pet animals in Saudi Arabia. Ejaz et al.^14^. detected *bla*_SHV_, *bla*_TEM_, and *bla*_CTX−M_ in 14.3% (1/7), 28.6% (2/7), and 57.1% (4/7) of *C. freundii* isolates originated from domestic and farm animals. Upon comparing the prevalence of beta-lactamase genes among beta-lactamase-producing *C. freundii* isolates, alarmingly, the current study found that all samples from both broiler chickens and buffalo (100%) were also classified as beta-lactamase producers based on their characteristics and genotyping. This agrees with the high prevalence detected for *bla*_TEM_ (100%) in both broiler chickens and buffalo. These genes coexisted in multiple isolates. The *bla*_TEM_ gene is responsible for more than 80% of resistance in enteric pathogens^57^. Contrary to the frequency of phenotypically ESBL-producing *C. freundii* isolates in the present study, Kanamori et al.^58^ detected ESBL-producing *C. freundii*with percentage of 4.6%. Also, Ejaz et al.^14^. investigated phenotypically ESBL-producing *C. freundii* in 3% and 2.6% of farm animals and domestic animals, respectively.

The global emergence of carbapenem resistance in *C. freundii* underscores the need for prudent use of carbapenems as part of antibiotic stewardship and infection control programs^59^. Although all *C. freundii* isolates in the present study were confirmed to harbor ESBL- and ampC-encoding genes (including *bla*_TEM_, *bla*_OXA_, *bla*_CTX−M_, *bla*_SHV_, and *bla*_CMY−2_), none of them tested positive for the common carbapenemase determinants (*bla*_KPC,_ *bla*_NDM−1,_ *bla*_OXA−48,_ *bla*_IMP,_ *bla*_GES,_ and *bla*_VIM_). Notably, despite the absence of common carbapenemase genes, some isolates exhibited phenotypic carbapenem resistance, suggesting involvement of non-enzymatic mechanisms. Previous reports have demonstrated that the loss or alteration of outer membrane porins can markedly reduce the penetration of carbapenems into the bacterial cell, while the up-regulation of efflux pumps can further expel the drug, thereby enhancing resistance. When combined with the hydrolytic activity of ESBL and ampC enzymes, these mechanisms can confer clinically significant carbapenem resistance even in the absence of carbapenemase genes. Resistance to carbapenems in *Citrobacter* spp. may also result from alterations in outer membrane porins^24^ and increased cephalosporinase production^60^, highlighting the multifactorial nature of resistance and the need to consider both enzymatic and non-enzymatic mechanisms when interpreting antimicrobial susceptibility results.

### Detection of association between ESBL/ampC and integron integrase class 1 genes

MDR mobile genetic elements, such as plasmids and integrons, can collect and transfer resistance gene cassettes, integrating ESBL genes and other antibiotic resistance determinants^61^. Notably, resistance to streptomycin and β-lactam antibiotics has been strongly associated with the presence of the *int*1 gene^62^. In the present study, *int*1 was detected in 100% of broiler chicken isolates and 90% of buffalo isolates, which represents a significant finding. These results are consistent with a previous report documenting *int*1 in 100% (7/7) of ESBL-producing *C. freundii* isolates from animals^14^. Detecting class 1 integrons in the majority of bacterial samples indicates that these mobile genetic elements are producing a lot of resistance^14^. The widespread presence of class 1 integrons among these isolates underscores their role in disseminating antimicrobial resistance and highlights the potential for reducing the effectiveness of available antibiotics, contributing to a post-antibiotic era. The high detection rate observed may reflect a recent trend of rapidly increasing integron-positive rates among *C. freundii* isolates in Egypt.

### Detection of association between ESBL/ampC and colistin resistance gene

The detection of the colistin resistance gene *mcr-*1 in ESBL-producing *C. freundii* isolates 10.53% of broiler chicken-derived and 5% of buffalo-derived isolates highlights the potential emergence of colistin resistance in livestock. These *mcr-*1-positive isolates were also identified as producers of *bla*_TEM,_ *bla*_CTX−M,_ *bla*_CMY−2,_ or *bla*_OXA−10_ β-lactamases, reflecting a MDR phenotype. Elevated MDR associated with *mcr-*1 acquisition may contribute to pleiotropic resistance across multiple antibiotic classes^63^. Although colistin use in veterinary practice is now restricted in Egypt to limit resistance, monitoring for *mcr-*1 remains essential to assess the potential for horizontal transfer of colistin resistance between livestock and humans, providing key epidemiological insights into the dissemination of critical resistance genes. The prevalence observed in this study is lower than that reported by Sarker et al.^64^, who detected *mcr*-positive isolates in 25% of *Citrobacter* spp., emphasizing geographic and management-related differences in colistin resistance emergence. Globally, the prohibition of colistin as a growth promoter or preventative treatment in animal feeds has proven effective in reducing resistant strains^65^, underscoring the need to determine the drivers, mechanisms of spread, and emergence of colistin-resistant infections to guide both veterinary and public health interventions.

### Association between ESBL/ampC and PMQR genes

The presence of concurrent PMQR markers in ESBL-producing *Enterobacteriaceae* suggests a transnational threat^66^. In the present study, all broiler chicken-derived *C. freundii* isolates (n = 19, 100%) and all buffalo-derived isolates (n = 20, 100%) were phenotypically confirmed as PMQR producers, with high levels of resistance to levofloxacin, ciprofloxacin, and nalidixic acid. Genotypically, the plasmid-mediated *qnr*A gene was detected in 84.21% of chicken isolates and 80% of buffalo isolates. An important observation in our study was the discrepancy between phenotypic ciprofloxacin resistance (100% in buffalo isolates) and the detection of the plasmid-mediated *qnr*A gene (80%). The observed discrepancy between phenotypic ciprofloxacin resistance (100% in buffalo isolates) and *qnr*A carriage (80%) suggests additional mechanisms, including chromosomal point mutations in quinolone resistance–determining regions (QRDRs) not captured in this study, the presence of other PMQR determinants (e.g., *qnr*B, *qnr*S, *aac(6’)-Ib-cr*), or efflux pump overexpression. These findings illustrate the complexity of fluoroquinolone resistance, where the presence of a resistance gene does not always equate to phenotypic expression. Resistance is influenced by environmental factors and is less sensitively detected by the phenotypic method^67^. Some isolates carrying resistance genes appeared phenotypically sensitive, potentially due to gene silencing, low expression, or mutations impairing function, whereas some phenotypically resistant isolates lacked the corresponding genes, indicating alternative mechanisms such as efflux, reduced membrane permeability, or target site mutations. Comparatively, the prevalence of PMQR genes in our study is higher than reported by Jacoby et al.^68^ and Kanamori et al.^58^, who did not detect *qnr*A in human clinical isolates, and Liu et al.^16^, who observed *qnr* genes in *C. freundii* at a much lower frequency (14.6%). These findings emphasize the significance of combining molecular and phenotypic analyses to fully understand resistance mechanisms and highlight potential environmental and veterinary reservoirs that may facilitate the dissemination of fluoroquinolone resistance.

### Detection of association between ESBL/ampC and *dfr*A1, *aad*A1, *sul*2, *cat*A1, *tet*(M), and *erm*B encoding resistance genes

The high prevalence of antibiotic resistance in *C. freundii* isolates emphasizes their potential role as a reservoir for antimicrobial resistance determinants in both clinical and environmental contexts^69^. Both *dfr*A1 and *aad*A1 were detected in 100% of the isolates, indicating widespread dissemination. Remarkably, all isolates exhibited complete (100%) phenotypic resistance to gentamicin, yet only the *aad*A1 gene was identified. Since *aad*A1 primarily confers resistance to streptomycin and spectinomycin, its presence alone cannot explain the uniform gentamicin resistance, suggesting that additional aminoglycoside resistance determinants such as *aac(3)-II*,* aac(6’)-Ib*, or *aph* variants or efflux mechanisms are likely involved. This phenotypic–genotypic discrepancy is consistent with previous observations in *Enterobacteriaceae*, highlighting the complex nature of aminoglycoside resistance and underscoring the need for broader molecular investigations, including expanded PCR screening or whole-genome sequencing.

Analysis of other resistance genes revealed high prevalence of *sul*2, detected in 89.47% of chicken-derived and 95% of buffalo-derived isolates, reflecting extensive sulfonamide resistance. The *erm*B gene was present in 52.63% of chicken isolates and 50% of buffalo isolates, indicating moderate dissemination of macrolide resistance. *tet*(M) gene was detected in 21.05% of chicken isolates and 5% of buffalo isolates, while *cat*A1 showed 5.26% prevalence in chicken isolates and 30% in buffalo isolates.

The drivers behind these striking resistance profiles and discrepancies merit critical examination. The exceptionally high resistance rates observed in this study, including 100% resistance to critically important antimicrobials such as ciprofloxacin and gentamicin in buffalo isolates. While simple comparisons can be misleading due to differing methodologies, the stark contrast prompts a critical discussion on the potential drivers unique to our geographic context. A key factor is likely the intensive and often unregulated use of antimicrobials in the animal production systems of the region, creating a powerful selective pressure that favors the survival and spread of resistant strains. Furthermore, the diverse array of resistance gene profiles detected (e.g., the variable carriage of *qnr*A, *aad*A1, *tet*(M), and even the rare *mcr-*1) suggests that the dissemination of resistance is not primarily driven by a single clonal outbreak. Instead, the evidence points to the prominent role of horizontal gene transfer via mobile genetic elements such as plasmids and integrons. These elements can act as efficient vehicles for shuffling resistance genes between different bacterial species, leading to the accumulation of extreme resistance profiles in a relatively short time. A primary limitation of this study is that its genetic screening was targeted and did not include molecular typing methods (e.g., PFGE, whole-genome sequencing) required to definitively distinguish between clonal spread and horizontal transfer. Therefore, future molecular epidemiological investigations are essential to confirm these mechanisms and trace the precise transmission pathways of resistance in this ecosystem. These findings suggest species-specific differences in resistance gene carriage, potentially reflecting variations in antimicrobial use and selective pressures between poultry and buffalo environments. Collectively, these results underscore the complexity of antimicrobial resistance in *C. freundii* and the limitations of single-gene detection in explaining phenotypic resistance. The high prevalence of multiple resistance determinants, alongside phenotypic–genotypic discrepancies, highlights the necessity for comprehensive molecular characterization to fully elucidate the mechanisms underlying resistance in this clinically and epidemiologically relevant pathogen.

This analysis further reveals the complex relationship between phenotypic resistance and the presence of corresponding resistance genes. While a strong association was observed in many cases—for instance, the high prevalence of the *aad*A1 gene correlated with streptomycin resistance—notable discrepancies were also evident. Some isolates exhibited phenotypic resistance without harboring the targeted genes, suggesting the potential involvement of alternative mechanisms, such as efflux pumps, undetected resistance genes, chromosomal mutations, or enzyme-mediated inactivation. Conversely, the detection of resistance genes in a few phenotypically sensitive isolates may indicate silent or unexpressed genetic determinants under the tested conditions. These findings underscore the limitations of relying solely on PCR-based gene detection and highlight the multifactorial nature of antimicrobial resistance in *C. freundii*, necessitating integrative approaches that combine genotypic screening with phenotypic assays for accurate resistance profiling.

### Characterization of CSNPs

In the current study, a wide UV-visible absorption peak at 223 nm was observed, indicating the successful formation of freshly generated CSNPs and reflecting nanoparticle transitions from the ground to an excited state, as described by Little et al.^70^. The presence of a single SPR band further suggests that the nanoparticles are predominantly spherical. This observation aligns with the UV-visible spectra reported by Vaezifar et al.^71^, who noted a peak at 226 nm. Particle size analysis using PSA revealed an average hydrodynamic diameter of 194.8 nm, which is larger than TEM-based measurements due to the notable swelling behavior of chitosan nanoparticles in suspension. Similarly, Essa et al.^72^ reported that CSNPs measured by dynamic light scattering (DLS) were 477 nm, compared to 200–280 nm by TEM, reflecting the distinction between hydrodynamic radius assessment (DLS) and projected area diameter measurement (TEM). When a dispersed particle in DLS moves through a liquid medium, a tiny electric dipole layer of the solvent attaches to its surface, which influences particle mobility^72^.

The synthesized CSNPs displayed a high zeta potential of 43 mV with minimal deviation (6.44 mV) at 25 °C and 1.14 mS/cm conductivity, indicating a very stable dispersion. This value is comparable to the 40 mV reported by Loutfy et al.^73^. As emphasized by Godoy et al.^74^, a zeta potential exceeding ± 20 mV denotes a highly charged surface, contributing to nanoparticle stability and minimizing aggregation.

FTIR analysis confirmed the presence of characteristic functional groups in chitosan-TPP nanoparticles, evidencing successful ionic gelation and interactions between chitosan and TPP, consistent with previous studies^75–78^. TEM imaging revealed predominantly spherical nanoparticles with smooth surfaces and a uniform size distribution averaging 34.95 ± 6.49 nm. The smaller size compared to that reported by Loutfy et al.^73^ (150 nm) likely reflects differences in CSNP synthesis protocols or raw material sources. Collectively, these physicochemical characterizations demonstrate the successful fabrication of stable, nanosized CSNPs with features conducive to enhanced antibacterial activity, including small particle size, high surface charge, and preserved functional groups, which likely underpin their superior efficacy against multidrug-resistant *C. freundii*.

### Antibacterial efficacy of CS and CSNPs against *C. freundii*

A high MAR index in our study highlights an urgent issue, particularly with zoonotic bacteria like *C. freundii* that exhibit resistance to multiple critically important antibiotics used in both animals and humans. Consequently, prudent antibiotic use and the establishment of robust surveillance networks are essential to mitigate the negative impact of antimicrobial misuse while ensuring the safety of animal-derived food. Given the increasing resistance issues in the veterinary sector, exploring naturally occurring antimicrobials as alternatives is a promising strategy to reduce bacterial resistance and maintain effective therapy in both livestock and humans. Our results demonstrate that CS and CSNPs, particularly when combined with CIP, exhibited enhanced antibacterial activity against *C. freundii* isolates from both broiler chickens and buffalo. The agar well diffusion assay revealed that the inhibition zones were larger for the CS-CIP and CSNP-CIP combinations compared to each agent alone, indicating enhancing interaction. In broiler chickens and buffalo, the combined antibacterial activity of CS 1%, CS 2%, and CSNPs with CIP was 1.83 and 1.66; 2.29 and 2.09; and 2.52 and 2.33 times greater than CIP alone. These findings are supported by previous studies, such as Ali et al.^79^, who demonstrated that CSNPs inhibited *C. freundii* at 400 µg/mL with a maximum inhibition zone of 26 mm. Our study extends this observation by showing that CSNPs, especially in combination with CIP, can achieve superior antibacterial effects against multidrug-resistant *C. freundii*. Unlike Ahmed et al.^80^, who reported no inhibitory effect of CS or CSNPs against *C. freundii*, our study overcame this limitation through strategic combination therapy. Additionally, while Ibrahim et al.^44^ showed efficacy of CIP-loaded CSNPs against *Escherichia coli* (*E. coli)*, our results demonstrate applicability against a clinically relevant pathogen, broadening the potential use in veterinary medicine.

Although these results provide strong evidence of enhanced antibacterial activity, caution is needed. Agar diffusion methods cannot conclusively establish true synergism; they suggest enhancing interactions at best. Therefore, future studies employing quantitative methods, such as checkerboard microdilution for FICI determination or time–kill assays, are necessary to confirm synergistic effects. Moreover, loading CIP into CSNPs may allow alternative administration routes, including oral, nasal, and ocular mucosa, pending safety evaluation in animal and human studies. Collectively, our findings highlight the novelty, strength, and practical significance of CSNP-based combination therapy. This approach represents a promising strategy to combat enteric bacterial infections, such as resistant *C. freundii*, in livestock and potentially in public health contexts, while reducing reliance on traditional antibiotics and mitigating the spread of resistance.

### Determination of MIC and MBC of CS 1%, CS 2%, and CSNPs against resistant *C. freundii*

The current study revealed that CSNPs achieved markedly lower MIC and MBC values against MDR *C. freundii* isolates from both broiler and buffalo samples compared with CS 1% and CS 2%, confirming their superior antibacterial efficacy. This enhanced activity can be attributed to the nanoscale size and increased surface area of CSNPs, which facilitate stronger interaction with bacterial cell membranes and more efficient disruption of cell integrity. This underscores the pivotal role of nanoparticle technology in overcoming bacterial resistance, where conventional agents alone have often proven insufficient. Although CS 2% showed slightly lower MIC and MBC values than CS 1%, the differences between the two concentrations were not statistically significant, indicating that increasing CS concentration alone does not lead to a substantial improvement in antibacterial activity. By contrast, CSNPs differed significantly from both CS 1% and CS 2% across all analyses (*p* < 0.001), underscoring the key role of nanoparticle formulation in enhancing antimicrobial performance. Importantly, the MBC/MIC index was ≤ 2 for all treatments in both broiler and buffalo isolates, demonstrating that CS 1%, CS 2%, and CSNPs all exerted bactericidal rather than bacteriostatic effects. The consistently lowest MIC and MBC values observed for CSNPs further reinforce their superior and stable bactericidal activity compared with conventional CS formulations. These findings collectively support the potential of CSNPs as a more potent antimicrobial alternative for controlling resistant *C. freundii* in both poultry and livestock production systems. These observations highlight the enhanced physicochemical and biological properties of nanoparticles, including increased surface area, improved bioavailability, and stronger interactions with bacterial cell membranes, likely contributing to their heightened efficacy. Comparison with previous studies emphasizes the novelty and robustness of our findings. Yassin et al.^43^ reported that chitosan-coated ciprofloxacin reduced the MIC against *Klebsiella pneumoniae* to 1.88 µg/mL, compared with 128 and 32 µg/mL for free ciprofloxacin. Our study extends this, showing that CSNPs alone—without antibiotic loading—can inhibit resistant *C. freundii* at even lower concentrations. Conversely, Ahmed et al.^80^ found no antibacterial effect of CS or CSNP suspensions against *C. freundii* even at 10 mg/mL, highlighting the influence of nanoparticle synthesis, characterization, and strain-specific resistance on outcomes. Similarly, Ali et al.^79^ reported no visible inhibition or only 80% growth reduction, whereas our results demonstrated consistent bactericidal activity at concentrations nearly two orders of magnitude lower. Overall, these findings confirm the concentration-dependent antibacterial activity of CS, the superior efficacy of the nanoparticle formulation, and the potential of CSNPs as a natural, cost-effective alternative to conventional antibiotics. This study establishes a new benchmark in antimicrobial nanotechnology, with significant implications for food safety, livestock health, and control of zoonotic pathogens, particularly MDR *C. freundii*. The demonstrated potency of CSNPs offers a promising strategy to reduce reliance on traditional antibiotics and address the global challenge of antimicrobial resistance in both veterinary and public health settings.

### Scanning electron microscopy of *C. freundii*

SEM analysis revealed the bactericidal effects of CS and CSNPs, showing distinct morphological alterations in treated cells compared with untreated controls. The findings of 0.156 mg/ml of CS on *C. freundii* show that the efficiency of CS is concentration dependent, especially when treating bacteria with a high prevalence of ESBL/ampC and other key antibacterial resistance genes. According to the findings, the degree of growth inhibition provided by CSNPs can successfully battle the development of pan-drug-like-resistant C. *freundii* as concentrations increase. Variations between this and previous studies may be attributable to differences in isolate numbers, nanoparticle preparation methods, laboratory conditions, or nanoparticle physicochemical properties^81^. These observations were consistent across all biological replicates and multiple examined fields of view, confirming the reproducibility of the morphological alterations. While the SEM findings are primarily qualitative, the frequency of deformed cells was notably higher in CSNP-treated populations compared with CS-treated ones. The current study demonstrated that CSNPs exhibited superior antibacterial activity compared with CS, likely due to their smaller size, larger surface area, and stronger affinity for bacterial cell membranes^82^. These observations are consistent with Chandrasekaran et al.^83^, who reported higher antibacterial activity of CSNPs compared with chitosan and chitin. Differences with other studies may stem from isolate numbers, nanoparticle synthesis methods, lab conditions, or nanoparticle properties.

**Table 9:** Analysis of MIC and MBC of CS and its nanoparticles against *C. freundii* isolates

Source	Agents (mg/ml)	Minimum	Q1	MIC50	Q3	MIC90	IQR	Maximum
MIC of buffalo	CS 1%	0.52	2.92	3.33	3.65	5.21	0.73	10
	CS 2%	0.63	2.08	2.92	3.65	4.58	1.57	5.83
	CSNPs	0.05	0.06	0.13	0.15	0.17	0.02	0.21
MIC of broiler chicken	CS 1%	0.63	2.5	4.17	6.67	8.33	4.17	8.33
	CS 2%	0.42	1.88	2.92	4.58	5.83	2.7	6.88
	CSNPs	0.02	0.06	0.08	0.11	0.14	0.05	0.17
		Minimum	Q1	MBC50	Q3	MBC90	IQR	Maximum
MBC of buffalo	CS 1%	2.5	3.333	4.167	5	5.667	1.667	6.667
	CS 2%	2.083	3.333	3.833	4.167	4.583	0.834	6.667
	CSNPs	0.052	0.073	0.146	0.167	0.208	0.094	0.250
MBC of broiler chicken	CS 1%	2.917	4.167	5.833	6.667	6.667	2.5	6.667
	CS 2%	1.667	3.333	5	6.667	6.667	3.334	6.667
	CSNPs	0.073	0.135	0.167	0.188	0.208	0.053	0.208

## Materials and methods

### Study design and sampling

Between August of 2023 and February of 2024, 540 samples were taken from diseased broiler chickens (aged 1–42 days) and Egyptian native buffaloes (aged 1.5–2.5 years) in different locations in the Northern Egyptian governorates of Kafr El-Sheikh and Dakahlia. To ensure human handling, diseased chicks were euthanized using cervical dislocation following ethical guidelines. Clinically diseased broiler chicken samples were collected from private veterinary clinics that supervise large-scale commercial farms, where birds were reared in high-production flocks, ensuring representative sampling from the commercial production system. The collected samples included 35 cloacal swabs and 280 internal organ samples from the liver, spleen, kidney, gall bladder, intestine, lung, meat, and gizzard (35 samples, each). Furthermore, 225 buffalo samples were collected from the liver, muscle, gall bladder, abomasum, omasum, reticulum, rumen, jejunum, and fecal matter (25 samples, each). All buffalo samples were collected freshly after slaughter at the official abattoirs. The animals were apparently healthy, and no clinical signs were noted before slaughter. Approximately 25 g of each tissue sample (intestine, liver, spleen, and kidney) were collected aseptically from each bird or buffalo. After being recognized, every sample was sent right away for bacteriological investigation in an icebox to the Laboratory of Bacteriology, Immunology, and Mycology, Faculty of Veterinary Medicine, Mansoura University.

### Isolation and identification procedures

Samples were first enriched in Nutrient broth (NB) (HiMedia, India) by inoculating approximately 1 g of tissue or 1 mL of liquid material into 9 mL of broth. A loopful of the incubated broth was then streaked onto MacConkey agar (HiMedia, India) for selective isolation. After 24 h of incubation, the positive findings appeared as pinkish colonies with a glossy surface (lactose fermenters), while pale colonies were incubated for another 24 h to determine late lactose fermenters. The cultures were subsequently sub-cultured onto Xylose Lysine Deoxycholate (XLD) agar (HiMedia, India) and incubated overnight at 37 °C, yielding positive results as yellow colonies. To distinguish *Citrobacter* spp. from *E. coli*, the lactose fermenter isolates were sub-cultured on EMB (HiMedia, India) for 24 h at 37 °C, with *Citrobacter*spp. appearing as dusty brown colonies, as stated by Bettelheim et al.^84^.. The bacterial colonies that exhibited *Citrobacter* spp. features were stained and verified as *Citrobacter* spp. utilizing an array of biochemical assays described by MacFaddin^85^. Biochemical characterization of the isolates was performed using a panel of standard tests, including hydrogen sulfide production, catalase, oxidase, motility, urease activity, triple sugar iron (TSI) reactions, IMVIC tests (Indole, Methyl Red, Voges–Proskauer, and Citrate utilization), and gas production. The temperature for all experiments was 36 ± 1 °C. The isolated *Citrobacter* species have been stored at −80 °C in nutrient broth that contains 30% sterile glycerol to facilitate further validation.

### Molecular detection of *C. freundii*

All verified *Citrobacter* spp. isolates were grown in Luria-Bertani broth medium (Merck, Germany). Following 18–20 h of incubation at 37 °C, genomic DNA was extracted using the boiling process, as stated before^86^. The amount and clarity of the extracted DNA were determined using a NanoDrop 1000 spectrophotometer (Thermo Scientific, USA) at 260 nm. All isolates were tested for *C. freundii* using standard PCR with a particular combination of primers for amplification of the 23 S rRNA gene forward: 5′-GAAGAATCCGGACAAACATC-3′ and reverse: 5′-CCAGGATAGGAAACATCCAG-3′ primer sequences, with an amplified band size of 189 bp^87^. The amplifying process took place using a thermal cycler (Master Cycler, Eppendorf, Hamburg, Germany). The PCR reaction was carried out in a 25 µL tube using 12.5 µL of 2X ABT Red Mix (Applied Biotechnology Co. Ltd., Egypt), 1 µL of each primer, and 5 µL of DNA template. The remaining volume was filled with sterile deionized water, which was thoroughly blended using a vortex. The appropriate PCR technique for *C. freundii* 23 S rRNA gene amplification was initial denaturation at 95 °C for 1 min, following 35 cycles of denaturation at 95 °C for 40 s, annealing at 58 °C for 1 min, and extension at 72 °C for 40 s, with a final extension at 72 °C for 5 min and then held at 4 °C. To ensure consistency, 100 bp DNA ladders (Applied Biotechnology Co. Ltd., Egypt) were used as molecular markers. As a negative control, test DNA was replaced with 5 µL of nuclease-free water. PCR-amplified products were electrophoresed on 1% (w/v) agarose gels and stained with ethidium bromide. The gel was finally observed and captured using a UV transilluminator.

### Antimicrobial susceptibility test

The phenotypic antimicrobial susceptibility of *C. freundii* isolates was tested using the Kirby-Bauer disc diffusion assay against 19 antimicrobial drugs (Oxoid, Basingstoke, Hampshire, England, UK) from thirteen antibiotic classes. The findings were classified as sensitive or resistant based on the diameters of the zone of inhibition provided by the CLSI standards and interpretation criteria^88^. Non-susceptible isolates were those that were intermediate or resistant to a specific antibiotic^89^. For antimicrobial susceptibility testing, 100 µL of the bacterial suspension, adjusted to 0.5 McFarland standard, was evenly spread onto Mueller–Hinton agar (MHA; HiMedia, India) plates using a sterile glass spreader. The inoculated plates were air-dried at room temperature for 10–15 min prior to aseptic placement of antimicrobial discs with sterile forceps, ensuring uniform absorption and reliable diffusion of the antibiotics. The plates were incubated at 37 °C for 24 h. Antimicrobial susceptibility testing was performed using a panel of agents selected according to the WHO^29^ classification of antimicrobials’ importance in human and veterinary medicine. The full list of antimicrobials, along with their groups, abbreviations, and disc contents, is summarized in Table 10. The reference strain *E. coli* ATCC 25,922 was employed as a quality control strain to ensure the accuracy and reliability of the antimicrobial susceptibility testing^90–92^. *C. freundii* isolates were classified into three groups depending on their phenotype—antimicrobial-resistant identities: MDR, which showed resistance to at least three or more antibiotic groups; XDR, which at first displayed resistance to all verified antibiotic classes except one or two; and PDR, which revealed resistance to all antibiotics across all antibiotic classes investigated^93^.

**Table 10:** Antimicrobial agents used for susceptibility testing according to WHO classification.

WHO classification	Antimicrobial group	Antimicrobial (full name)	Abbreviation	Disc content (µg)
**Critically important**	Penicillins	Amoxicillin	AMX	10
		Carbenicillin	CB	100
	Carbapenem	Meropenem	MEM	10
	Oxazolidinones	Linezolid	LNZ	30
	Macrolides	Clarithromycin	CL	15
		Azithromycin	AZM	15
	Aminoglycosides	Gentamicin	GEN	10
	Fluorquinolones	Levofloxacin	LEV	5
		Ciprofloxacin	CIP	5
	Quinolones	Nalidixic acid	NAL	30
	Cephalosporins 3rd generation	Cefpodoxime	CPD	10
		Cefixime	CFM	5
	Monobactam	Aztreonam	ATM	30
	Beta lactamase inhibitor	Ampicillin/sulbactam	SAM	10/10
		Piperacillin/tazobactam	TZP	100/10
**Highly important**	Tetracyclines	Minocycline	MI	30
	Lincosamides	Clindamycin	DA	2
	Folate pathway antagonists	Sulfamethoxazole/trimethoprim	SXT	23.75/1.25
	Cephalosporins, 1 st generation	Cefadroxil	CFR, CF	30

### Genotypic screening for antibiotic resistance genes (ARGs), ESBL-encoding genes, AmpC encoding gene, colistin resistance gene, and integron integrase gene

Using ten distinct classes, twenty ARGs were examined: aminoglycosides (*aad*A1), chloramphenicol (chloramphenicol acetyltransferase) (*cat*A1), plasmid-mediated quinolone resistance (PMQR) gene (*qnr*A), sulfonamide (*sul*2), tetracyclines (*tet*M), macrolide–lincosamide–streptogramin B methylase (*erm*B), trimethoprim (*dfr*A1), genes encoding β-lactamases, ESBL (*bla*_TEM_), (*bla*_SHV_), (*bla*_OXA−10_), (*bla*_CTX−M_), AmpC type (*bla*_CMY−2_), common carbapenemase determinants (*bla*_KPC,_ *bla*_NDM−1,_ *bla*_OXA−48,_ *bla*_IMP,_ *bla*_GES,_ and *bla*_VIM_). Colistin-resistant isolates were identified by the presence of *mcr-*1 and gene encoding class 1 integron integrase gene (*int*1). All strains were analyzed using multiplex PCR to identify the *dfr*A1, *qnr*A, and *sul*2 genes. A duplex PCR for the *bla*_TEM_ and *bla*_CTX−M_ genes; and standard uniplex PCR for the other genes were also conducted. Table 11 provides a summary of the primer sequences, target genes, amplicon size of the genes employed, annealing temperature, and pertinent references. PCR assays were performed in multiplex, duplex, and uniplex formats. Multiplex panels were first optimized for the simultaneous amplification of multiple resistance genes; duplex PCR was used for specific primer pairs that co-amplified reliably. Targets that failed to amplify consistently in multiplex/duplex assays were re-tested in uniplex PCR. For PCR assays, negative controls included a no-template control (NTC) and a reference susceptible strain (*E. coli* ATCC 25922) to ensure the absence of non-specific amplification. As positive controls, well-characterized internal isolates that had been previously confirmed to harbor the corresponding resistance genes were used, since no suitable ATCC reference strain was available for all targeted genes. This approach is consistent with standard practice when commercial or reference positive controls are not accessible. The use of an internal control in PCR is a standard approach to confirm amplification accuracy and detect possible reaction inhibition^94^. Of the 25 µL total reaction volume, 12.5 µL contained 2X ABT Red Mix, 5 µL of template DNA, 1 µL of each oligonucleotide primer, and 5.5 µL of nuclease-free water. The mixture was properly incorporated by vortexing. Initial denaturation for 5 min at 94 °C, 35 cycles of denaturation at 95 °C for 30 s, annealing temperatures listed in Table 5, and extension at 72 °C for 60 s were the conditions for all PCR amplification. The last extension was done for ten minutes at 72 °C. As previously mentioned, amplicons had been electrophoresed.

**Table 11:** Primers sequences, target genes, annealing temperature, and amplicon size of the used genes. multiplex, duplex, and uniplex PCR assays were applied depending on primer compatibility.

Antimicrobial classes	Target gene	Sequence (5′ to 3′)	Size (bp)	Annealing temperature	Reference
Beta-lactams	*bla*_CTX−M_ (ESBL)	F: ATGTGCAGYACCAGTAARGTKATGG R: TGGGTRAARTARGTSACCAGAAYCAGCGG	593	duplex PCR by 57 °C	^95^
	*bla*_TEM_	F: ATCAGCAATAAACCAGC R: CCCCGAAGAACGTTTTC	516		^96^
	*bla*_OXA−10_	F: TATCGCGTGTCTTTCGAGTA R: TTAGCCACCAATGATGCCC	760	57 °C	^97^
	*bla*_SHV_	F: AGGATTGACTGCCTTTTTG R: ATTTGCTGATTTCGCTCG	392	57 °C	^96^
ampC	*bla*_CMY−2_	F: AGCGATCCGGTCACGAAATA R: CCCGTTTTATGCACCCATGA	695	61 °C	^98^
Carbapenemase	*bla*_OXA−48_ (class D)	F: GCTTGACCCTCGATT R: GATTTGCTCCGTGGCCGAAA	281	60 °C	^99^
	*bla*_IMP_ (MBLs, class B)	F: TTGACACTCCATTTACDG R: GATYGAGAATTAAGCCACYCT	139	55 °C	
	*bla*_KPC_ (class A)	F: CATTCAAGGGCTTTCTTGCTGC R: ACGACGGCATAGTCATTTGC	538	55 °C	
	*bla*_GES_ (class A)	F: AGTCGGCTAGACCGGAAAG R: TTTGTCCGTGCTCAGGAT	399	57 °C	^100^
	*bla*_VIM_ (MBLs, class B)	F: GATGGTGTTTGGTCGCATA R: CGAATGCGCAGCACCAG	390	60 °C	^101^
	*bla*_NDM−1_ (MBLs, class B)	F: GGCGGAATGGCTCATCACGA R: CGCAACACAGCCTGACTTTC	287	55 °C	^102^
Streptomycin	*aad*A1	F: TATCCAGCTAAGCGCGAACT R: ATTTGCCGACTACCTTGGTC	447	55 °C	^103^
Phenicols	*cat*A1	F: AGTTGCTCAATGTACCTATAACC R: TTGTAATTCATTAAGCATTCTGCG	547	55 °C	^104^
Fluoroquinolones	*qnr*A	F: ATTTCTCACGCCAGGATTTG R: GATCGGCAAAGGTTAGGTCA	516	Multiplex PCR by 55 °C	^105^
Sulfonamides	*sul*2	F: CGGCATCGTCAACATAAACC R: GTGTGCGGATGAAGTCAG	722		^106^
Trimethoprim	*dfr*A1	F: GGAGTGCCAAAGGTGAACAGC R: GAGGCGAAGTCTTGGGTAAAAAC	367		^107^
Tetracyclines	*tet*(M)	F: GTGGACAAAGGTACAACGAG R: CGGTAAAGTTCGTCACACAC	406	55 °C	^108^
Erythromycin	*erm*B	F: GAAAAGGTACTCAACCAAATA R: AGTAACGGTACTTAAATTTGTTTTAC	636	57 °C	^109^
integron integrase gene class 1	*int*1	F: GCCTTGCTGTTCTTCTACGG R: GATGCCTGCTTGTTCTACGG	565	57 °C	^110^
Polymyxins (colistin)	*mcr-*1	F: CGGTCAGTCCGTTTGTTC R: CTTGGTCGGTCTGTAGGG	309	57 °C	^111^

### Chemicals used and synthesis of Chitosan and Chitosan nanoparticles solution

Sigma-Aldrich (St. Louis, MO, USA) provided low molecular weight (LMW) extra pure edible chitosan (CS) with at least 95% deacetylation (DA), acetic acid, sodium hydroxide (NaOH), and sodium triphosphate (TPP).

TPP was linked to CSNPs employing the ionotropic gelation technique, which is based on an electrostatic interaction between the positively charged chitosan and negatively charged TPP. A 1% w/v aqueous acetic acid solution, which is widely reported to act only as a solvent without exhibiting antibacterial activity at the concentrations used^112^, was prepared containing 0.25 mg/mL chitosan. The solution was magnetically agitated overnight at room temperature to obtain a clear solution, after which the pH was adjusted to 5 using 1 M NaOH. The resultant solution’s CS had been cross-linked with 1% TPP prior to passing via a 0.45 m syringe filter and sonicated at 1.5 kW for 30 min on an Ultrasonic Homogenizer HD 2070. The filtrate went through a centrifuge at 12,000 g for 10 min. Precipitate (CSNPs) was rinsed twice with distilled water before being centrifuged and freeze-dried. The freeze-dried CSNPs can be dissolved in water for characterization or employed immediately in other experiments. Chitosan concentrations at 1% (10 mg/ml) and 2% (20 mg/ml) were made by dissolving 1 g and 2 g of CS in 1% and 2% acetic acid solutions, respectively. The chitosan solution was produced freshly for every single day’s tests.

### Characterizations of CSNPs

#### Particle size analyzer and zeta potential

The particle size analyzer (PSA) and electrophoretic mobility (Zeta potential) (ZP) of freshly synthesized CSNP nanoparticles were investigated using dynamic light scattering (DLS) by Zetasizer-ZS Ver. 7.01 (Malvern Instruments Limited, UK).

#### UV-visible spectroscopy

The synthesis of nanoparticles was verified by observing how they were formed via UV-visible (UV-Vis) spectroscopy to detect their absorption band. A spectrophotometer (Edinburgh Instruments Ltd. DS5 Dual Beam UV–Vis spectrophotometer) with a resolution of 1 nm was applied to scan the UV–Vis absorption spectra of the synthesized CSNPs in a range of 190–800 nm.

#### Fourier-transform infrared spectroscopy

CSNPs’ Fourier-transform infrared (FTIR) spectra were captured using an FTIR spectrophotometer (Bruker-Tensor 27, Bremen, Germany) to identify certain chemical groups in the examined materials. The 4000–400 cm⁻¹ infrared range was explored.

#### Transmission electron microscopic observation of CSNPs

Using a transmission electron microscope (TEM) (JEOL-JEM-1200, JEOL Ltd., Tokyo, Japan), the diameters and morphology of CSNPs were examined. Following a quick sonication in ethanol to separate the gathered dried CSNPs, 200 µl of CSNPs were put onto a 400-mesh carbon-coated copper grid that had been wrapped with nitrocellulose film. The CSNPs were thereafter immediately studied using a TEM.

#### Evaluation of antibacterial assay of CSNPS, CS 1%, and CS 2%

The CSNPs, CS 1%, and CS 2% were tested for antibacterial activity against resistant *C. freundii* using an agar well diffusion test, according to protocol per Mohamed^113^. Overall, a sterilized L-shaped rod was used to swab 100 µL of *C. freundii* suspension with 10^8^ CFUs/mL (0.5 McFarland standard) properly across the MHA surface with the goal to achieve even bacterial growth. After punching a hole in the agar with a sterilized cork borer of a 6 mm diameter, a drop of molten MHA was poured into the wells for sealing the bottoms. After that, 100 µL of CS 1% (10 mg/ml), CS 2% (20 mg/ml), and CSNPs (0.25 mg/ml) were added to different wells. Additionally, ciprofloxacin (CIP, 5 µg/mL) was injected separately with 100 µL of CS 1%, 100 µL of CS 2%, and 100 µL of CSNPs in other wells. The negative control was DMSO (gel solvent), while the positive control was 100 µL of ciprofloxacin (5 µg/mL). After 30 min of refrigeration to ensure each of the compounds under study and the control material was sufficiently dispersed, the plates were placed in an incubator set at 37 °C for 24 h. The diameter of inhibition zone, measured in millimeters (mm), was used for assessing the growth-inhibitory effect of antimicrobial drugs after the incubation time was over. The bactericidal activity of commercial antibiotic CIP alone was compared to CIP plus CS (1%), CS (2%), and CSNPs by measuring fold area growth. The Inhibitory effect of CIP in combination with CS was expressed as the relative increase in the area of inhibition, calculated using the formula: area (mm²) = π × (zone diameter/2) ². The fold increase was obtained by dividing the inhibition area of the combination disc by that of CIP alone. For each group of isolates (broiler chickens and buffalo), the mean values of the inhibition areas were calculated from all samples within the group, and the final fold increase was expressed as the group average. All experiments were performed in triplicate.

#### Determination of minimum inhibitory concentration and minimum bactericidal concentration evaluation

The MIC and MBC of CS 1%, CS 2%, and CSNPs were determined against resistant *C. freundii* using the technique specified in the CLSI guidelines^88^ and established protocols^114^. Standard broth microdilution techniques were used to assess the minimum inhibitory concentration (MIC) of antibacterial drugs in a 96-well round-bottom microtiter plate (Lab Systems, Helsinki, Finland). For this test, 100 µL of Muller Hinton broth (MHB) was poured into each of the wells 1 through 12. Next, 100 µL of antibacterial agents (CS 1%, CS 2%, and CSNPs) was added to the first well. Two-fold serial dilutions were then carried out from wells 1 to 10, with concentrations ranging from 10 to 0.020 mg/mL, 20 to 0.039 mg/mL, and 0.25 to 0.00049 mg/mL, respectively. The bacterial suspension of *C. freundii* was prepared from fresh overnight cultures grown on NA plates. Colonies were picked and suspended in sterile saline to achieve an initial turbidity equivalent to 0.5 McFarland standard, then further diluted in MHB to reach the final inoculum concentrations required 10^6^ CFU/mL for the broth microdilution MIC/MBC assay. The turbidity of the bacterial suspension was confirmed visually and by using a spectrophotometer at 600 nm to ensure reproducibility and standardization across all experiments. Subsequently, 100 µL of bacterial inoculums were introduced to all wells excluding the negative control well. Well 1 of the microtiter plates exhibited the highest concentration of antibacterial compounds, whereas well 10 had the lowest concentration. Well 11 served as a positive control (medium and bacterial inoculum), whereas well 12 served as a negative control (just medium). After 24 h of incubation at 37 °C, 30 µL of resazurin solution (0.015% w/v) was added to each well of the 96-well plates as a viability indicator. The plates were then incubated for an additional 1–4 h to allow color development. Resazurin was conducted at 0.015% concentration by dissolving 0.015 gm of resazurin, then vortexing and filtering through (0.22 μm filter paper), and kept at (4 °C) for a maximum of two weeks following production^115^. Because of the reductase enzyme activity, blue resazurin is reduced to pink resorufin, indicating the viability of microbial cells. After four hours of incubation, the well that remained blue or purple (without changing color) showed the lowest concentration of antibacterial drugs that prevent microbial growth by suppressing apparent growth, and this well was assigned a MIC value^116^. Next, the capacity of CS 1%, CS 2%, and CSNPs 0.25 mg/mL to produce bactericidal activity was assessed by calculating MBC values against growing *C. freundii*. For MBC determination, 50 µL aliquots were carefully taken from wells exhibiting complete growth inhibition, as assessed by turbidity and visual inspection, prior to the addition of resazurin. MIC endpoints were initially confirmed using these physical observations. Resazurin was subsequently added to the remaining wells solely to validate bacterial viability. The collected aliquots were then subcultured onto fresh MHA plates, which did not contain resazurin, and incubated at 37 °C for 24 h to determine the MBC by confirming the complete absence of colony formation. Three copies of every test were conducted. MBC, which is supplementary to MIC, is the lowest dose of antibacterial drugs that totally kills 99.9% of bacterial inoculum and leaves any observable growths on the MHA plate^117^. Bacterial growth would indicate their presence in the original well and the bacteriostatic effect at that concentration. On the other hand, if absolutely no development was detected in the original well, the antibacterial drug might be considered bactericidal at that dose^82^. The bactericidal or bacteriostatic impact of the investigated substances was determined by calculating the MBC/MIC ratio. According to French^118^, if the MBC/MIC ratio is less than four times, the studied substances become bactericidal.

### Scanning electron microscopic observation of the antibacterial efficacy of CS and CSNPs on resistant *C. freundii*

The antibacterial activity of CS and CSNPS against *C. freundii* was separately assessed using a JEOL JSM-IT100 scanning electron microscope (SEM) (JEOL Ltd., Tokyo, Japan). *C. freundii* was grown in MHB with sub-MIC concentrations of CS and CSNPS, incubated for 18 h at 37 °C, and then lightly centrifuged (5000 r/5 minutes). Following that, the resulting pellets were washed three times with 0.1 M phosphate buffer saline (PBS) (pH 7.4) before being kept in 2.5% glutaraldehyde overnight at 4 °C. Then, the pellets were rinsed three times for 20 min each with 0.1 M PBS (pH 7.4) before being dehydrated using an ethanol gradient (30, 50, 70, 80, 95, 100, 100%) for 15 min at every step. Every specimen was dehydrated employing the critical-point drying process, sputter-coated with gold, and examined under SEM. For each treatment condition, multiple random fields of view were systematically examined. The micrograph presented for each group was selected as the most representative image of the predominant and consistent morphological alterations observed.

### Statistical analysis

All statistical analyses were performed using jamovi software (version 2.7.6; The jamovi Project, Sydney, Australia). The distribution of continuous variables was assessed with the Shapiro–Wilk test. Parametric tests were applied when the assumption of normality was satisfied, while non-parametric alternatives were used if any group deviated from normality. Descriptive statistics are presented as mean ± standard deviation (SD) for normally distributed variables, and as median with interquartile range (IQR) for non-normally distributed variables. Categorical data are expressed as frequencies and percentages. Within-species comparisons were conducted using a one-way repeated measures ANOVA to examine the effect of different antibiotic treatments on inhibition zones for broiler and buffalo isolates separately. This approach accounts for the non-independence of repeated measurements from the same isolate. When the assumption of sphericity was violated, the Greenhouse–Geisser correction was applied. Post-hoc pairwise comparisons were adjusted using the Bonferroni method. For MIC and MBC data that were not normally distributed, the Friedman test was used as a non-parametric alternative, followed by Durbin–Conover post-hoc tests for multiple comparisons. Between-species differences (broiler vs. buffalo) for each antibiotic, across inhibition zones, MIC, and MBC values, were evaluated using the Mann–Whitney U test. Associations between categorical variables were analyzed using the Chi-square test, or Fisher’s exact test when appropriate. A p value < 0.05 was considered statistically significant. The antimicrobial resistance profile, resistance type, and resistance genes were shown in a binary heatmap with annotations using Python version 3.10 and Google Colaboratory, with libraries such as Seaborn, Pandas, NumPy, and Matplotlib. Furthermore, the co-occurrence of antimicrobial resistance genes was analyzed and visualized using a correlation heatmap with hierarchical clustering, implemented in Python version 3.10 via Google Colaboratory. The analysis leveraged multiple libraries, including Seaborn, Pandas, NumPy, Matplotlib, linkage, dendrogram, fcluster, and squareform.

### Study limitations and future directions

This study, while providing significant insights into the resistance profiles of *C. freundii*, is subject to several limitations that should be considered when interpreting the findings. The primary constraints include the lack of phenotypic confirmation for colistin susceptibility and the use of a preliminary disk diffusion assay for synergy testing, which restricts definitive conclusions on resistance and drug interactions. Furthermore, the relatively small sample size and the targeted nature of the genotypic analysis limit the generalizability and comprehensiveness of the results.

A critical finding was the observed discordance between phenotypic resistance and genotypic profiles. Notably, the high prevalence of carbapenem and fluoroquinolone resistance could not be explained by the screened resistance determinants, as all isolates tested negative for major carbapenemase genes (*bla*_IMP,_ *bla*_VIM,_ *bla*_OXA−48,_ *bla*_NDM−1,_ *bla*_KPC_). This underscores the likely contribution of alternative mechanisms, such as chromosomal mutations, efflux pump overexpression, or other uncharacterized genetic factors.

To address these limitations, future research should integrate phenotypic colistin testing and employ quantitative methods such as the checkerboard assay and time-kill kinetics to accurately characterize drug interactions. Most importantly, the adoption of whole-genome sequencing (WGS) will be essential to comprehensively elucidate the genetic basis of resistance, identify novel mechanisms, and distinguish between clonal spread and horizontal gene transfer. Additionally, cytotoxicity assessments and in vivo studies are recommended to evaluate the therapeutic potential and safety of novel antibacterial agents investigated in this study.

## Conclusions

This study revealed an emergent prevalence of multidrug-, extensively drug-, and pan drug-resistant *C. freundii* in some broiler chickens and buffalo farms in Egypt, posing a severe danger to food safety, the veterinary sector, animals, humans, and the healthcare community. The finding of critical resistance genes—ESBL, ampC, PMQR, *mcr-*1, and *int*1 directs concern about their potential spread across *Citrobacter* species, other *Enterobacteriaceae* members, and into the human food chain, lowering the efficacy of critical antibiotics used to treat animal and human infections. These findings underscore the rigorous efforts to monitor this disease, particularly to prevent its spread by early detection, diligent antibiotic stewardship, and adequate infection control methods. Importantly, chitosan (CS) and its nanoparticle form (CSNPs) demonstrated high in vitro antibacterial efficacy against resistant *C. freundii*, with CSNPs outperforming, particularly when coupled with CIP. The CSNPs-CIP combination considerably increased antibacterial activity, implying better drug delivery and decreased resistance at lower doses. Consequently, CS and CSNPs represent promising adjuvant therapies to standard antibiotics for treating resistant *C. freundii* infections, and their potential should be validated through in vivo studies and field trials to assess efficacy, safety, and practical application in veterinary settings. Their incorporation into treatment procedures has the potential to improve therapeutic results while slowing the spread of resistance.

## Supplementary Information

Below is the link to the electronic supplementary material.

Supplementary Material 1

Supplementary Material 2

Supplementary Material 3

Supplementary Material 4

Supplementary Material 5

Supplementary Material 6

## Data Availability

All of the data collected for this paper is included in the present study. Please note that data requests related to this study can be directed to Amal Awad (e.mail: amalabdo@mans.edu.eg) andIbtisam Faeq Hasona (e.mail: [ebtisam.vet_0445@vet.kfs.edu.eg](mailto: ebtisam.vet_0445@vet.kfs.edu.eg))
