# Supplementary figures and images for: Chitosan mitigates pan drug resistance in citrobacter freundii exhibiting AmpC and ESBL from Egyptian livestock
Source: Sci Rep. 2025 Dec 5;15:43285. doi: 10.1038/s41598-025-28607-0 (PMC12686506; doi:10.1038/s41598-025-28607-0)

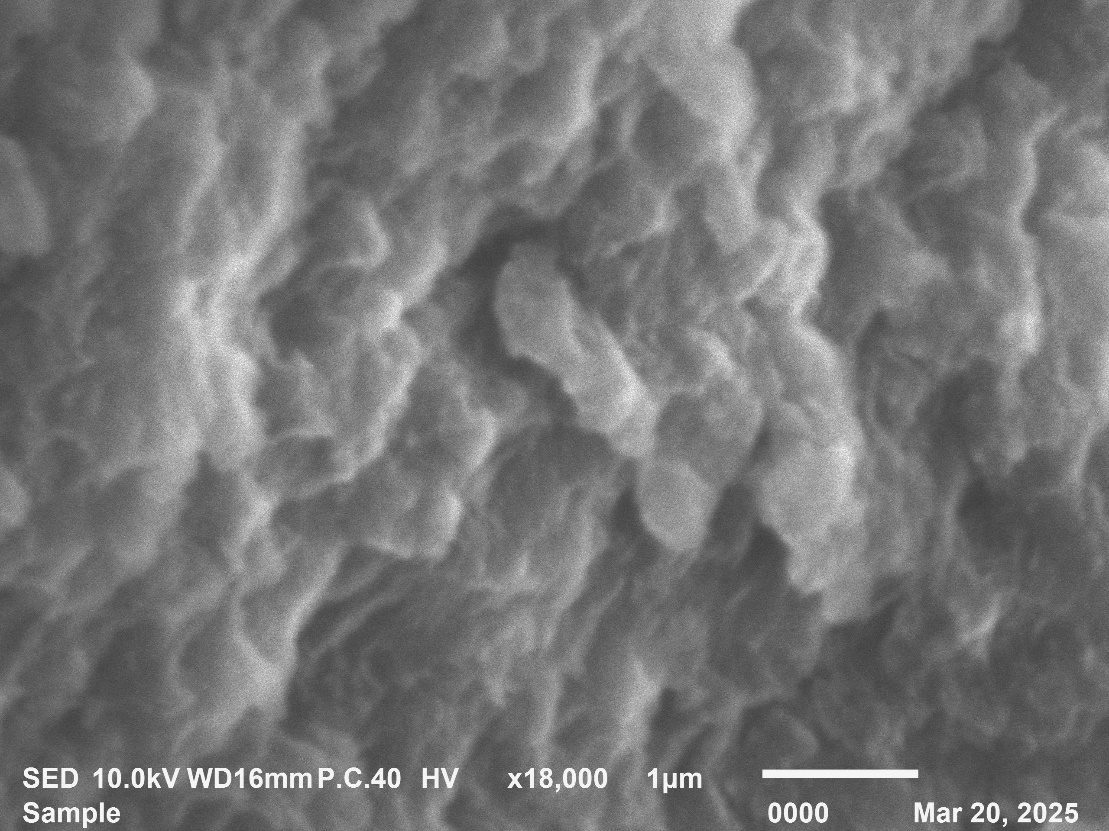

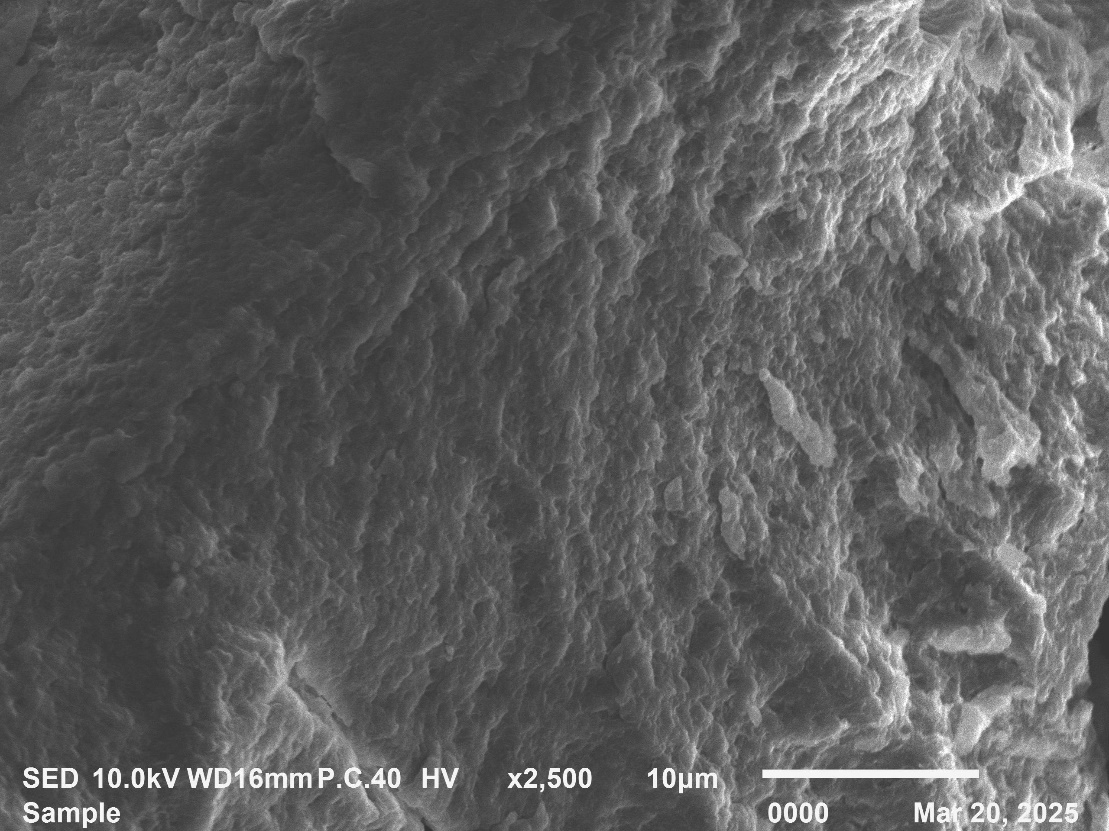

Supplement: Supplementary file 3 — Supplementary Material 3 [file 41598_2025_28607_MOESM3_ESM.docx]

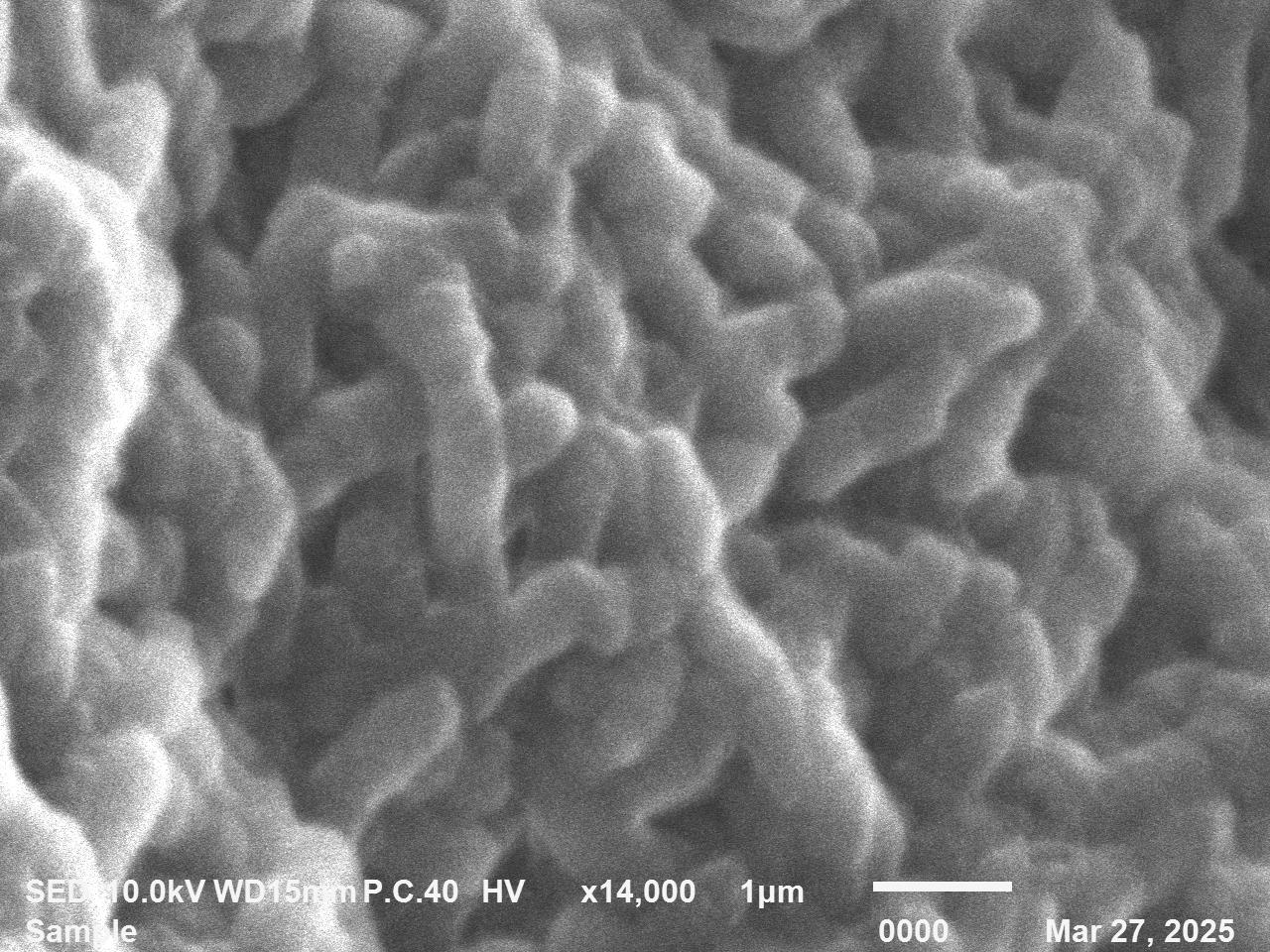

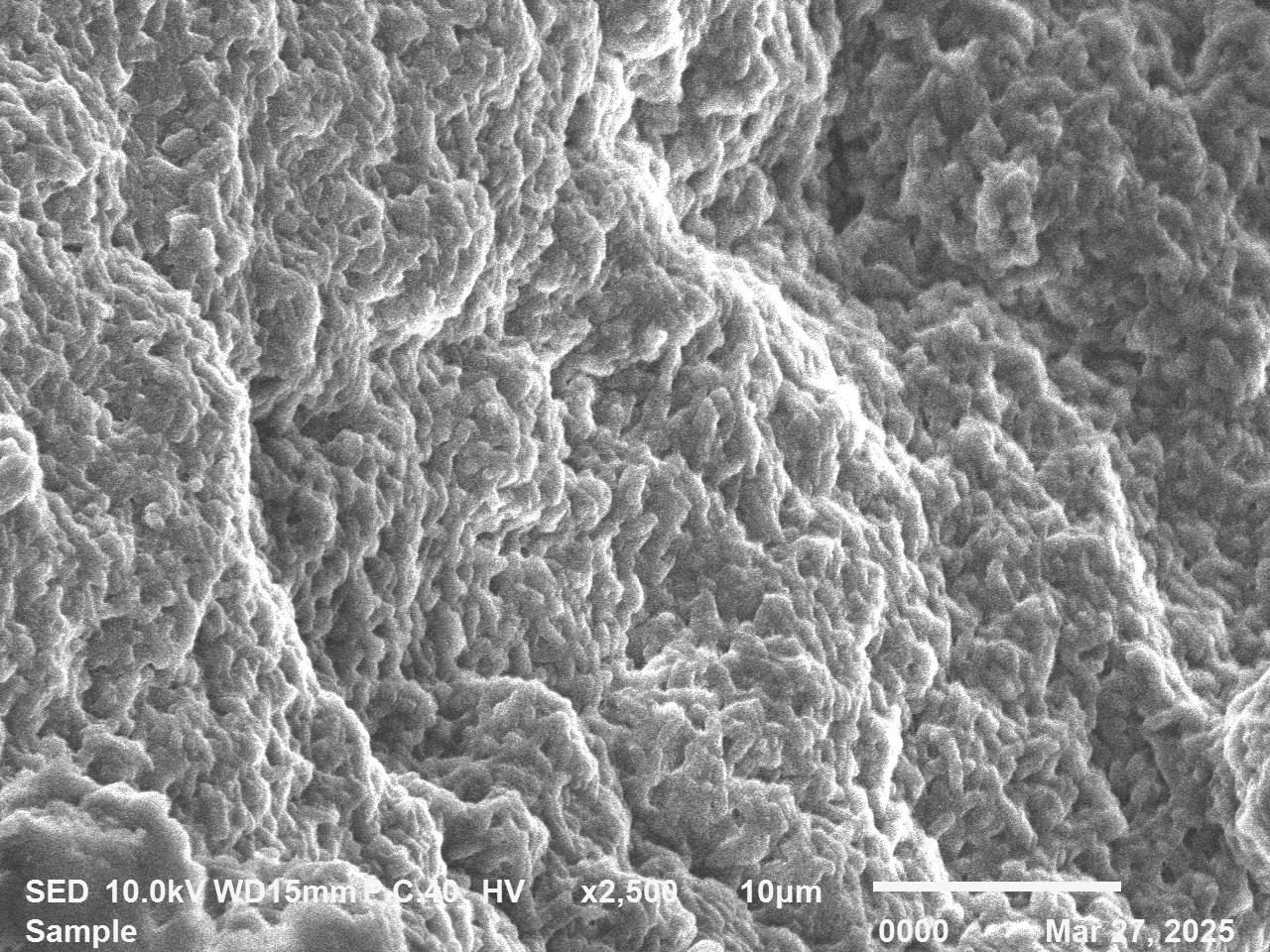

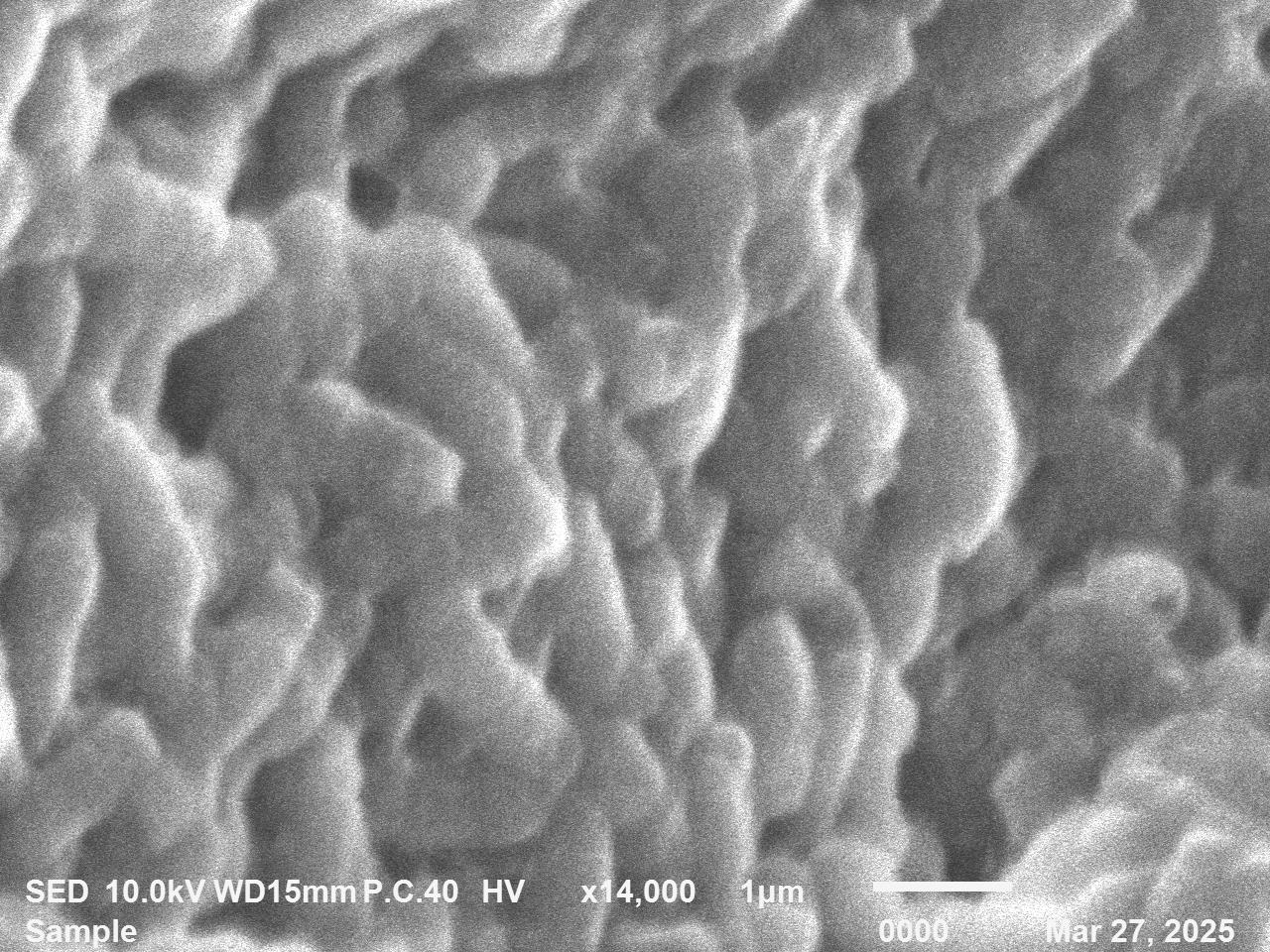

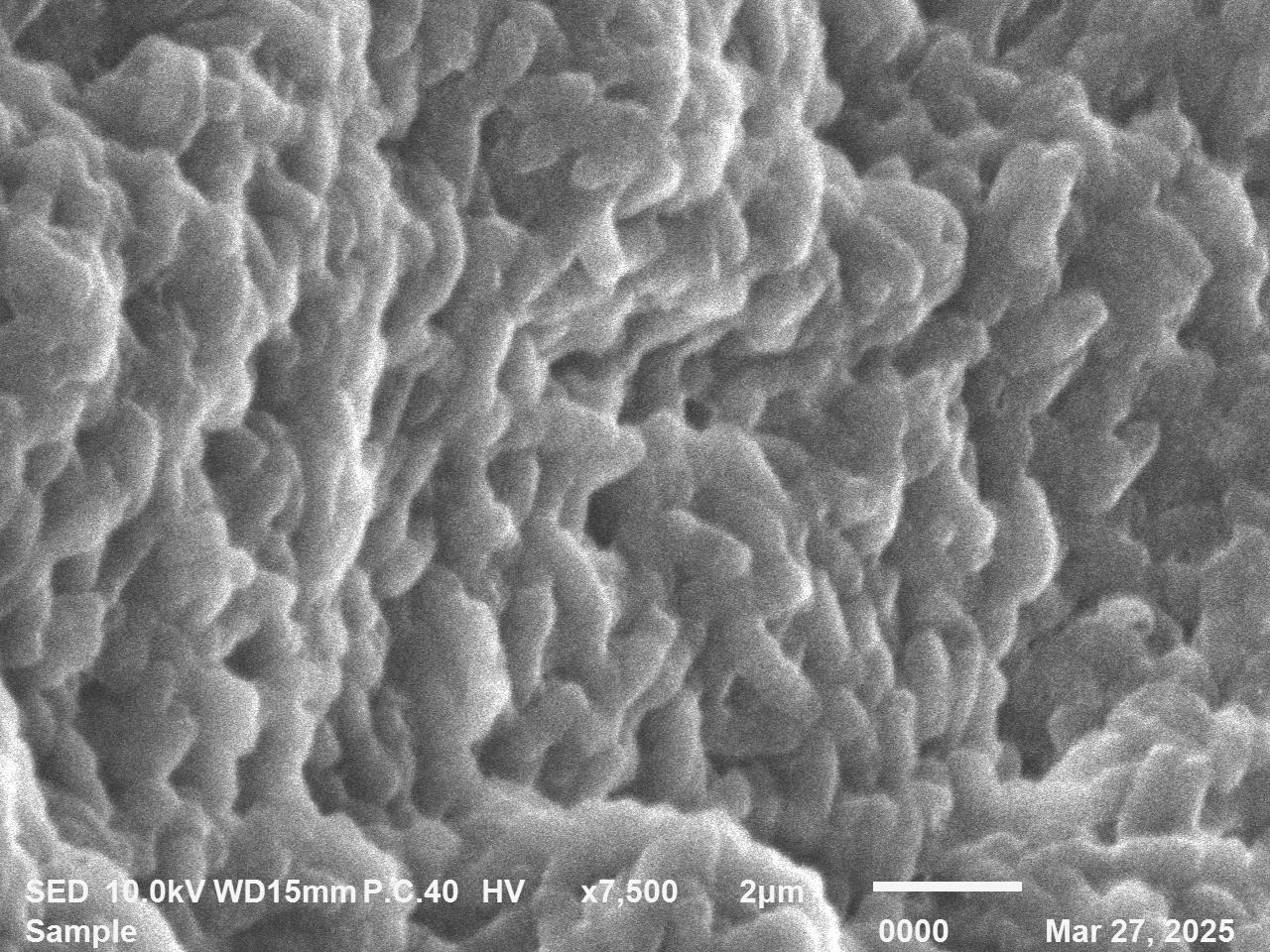

Supplement: Supplementary file 4 — Supplementary Material 4 [file 41598_2025_28607_MOESM4_ESM.docx]

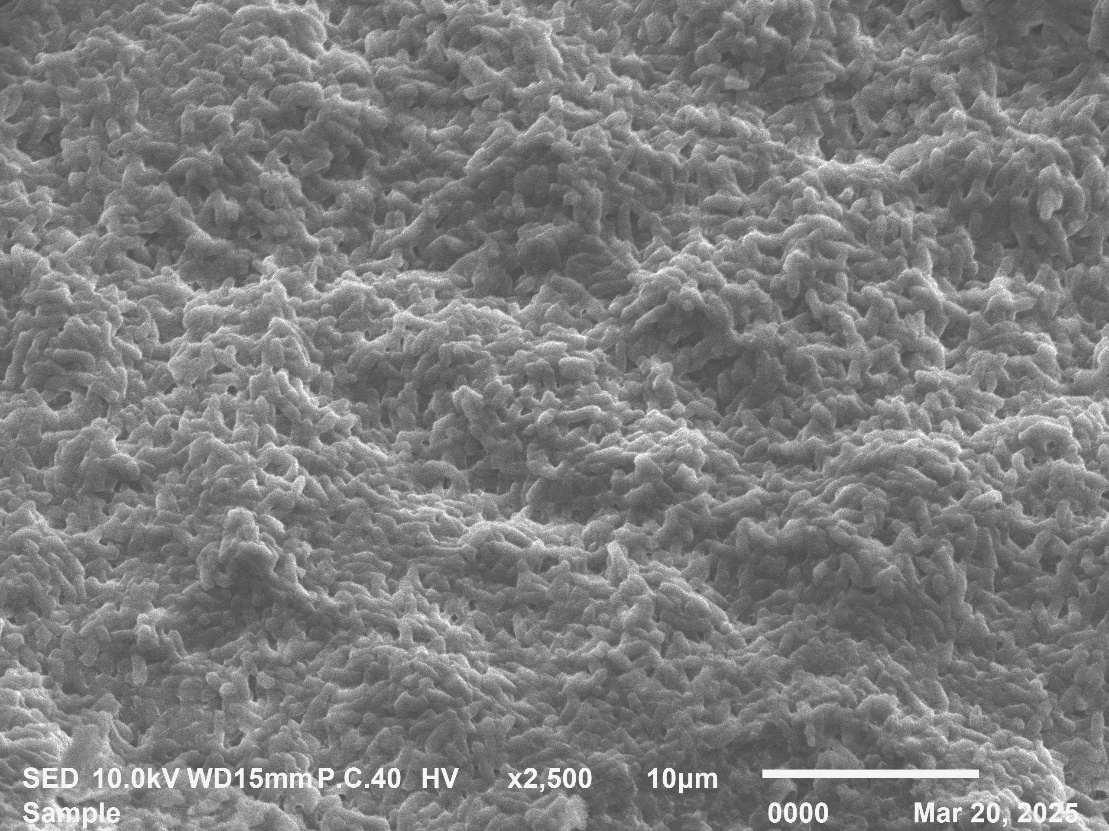

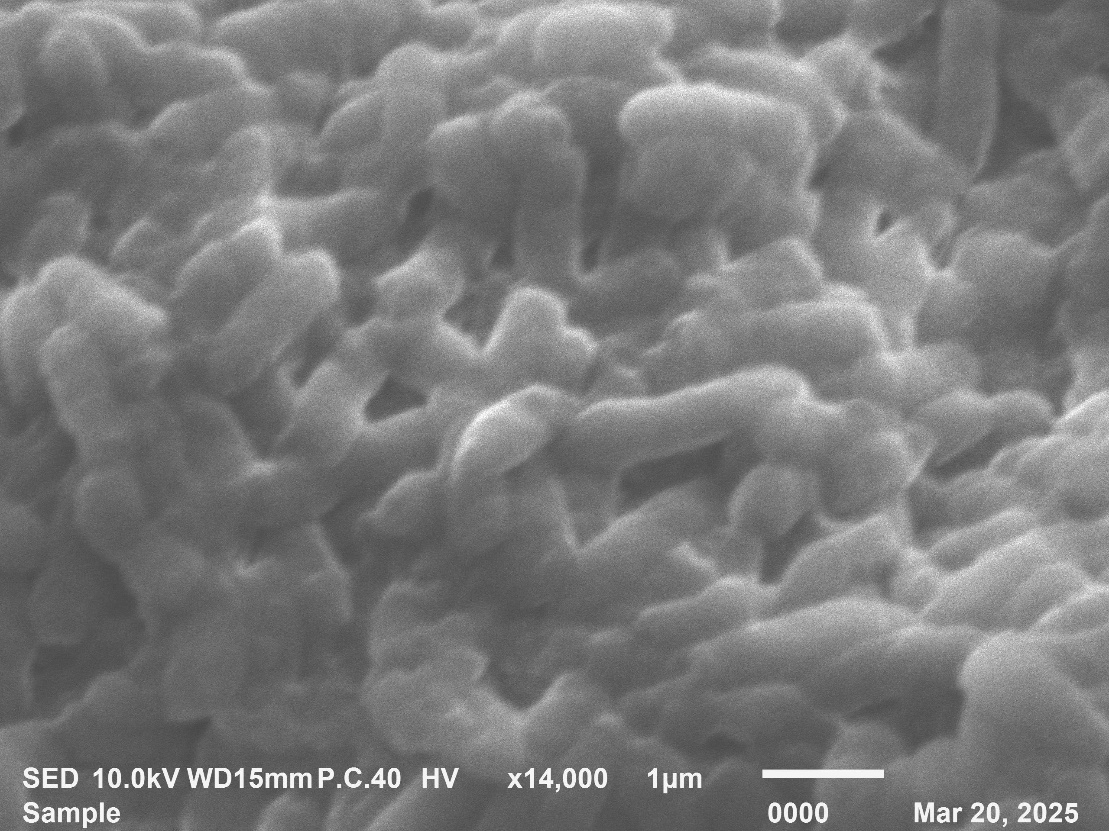

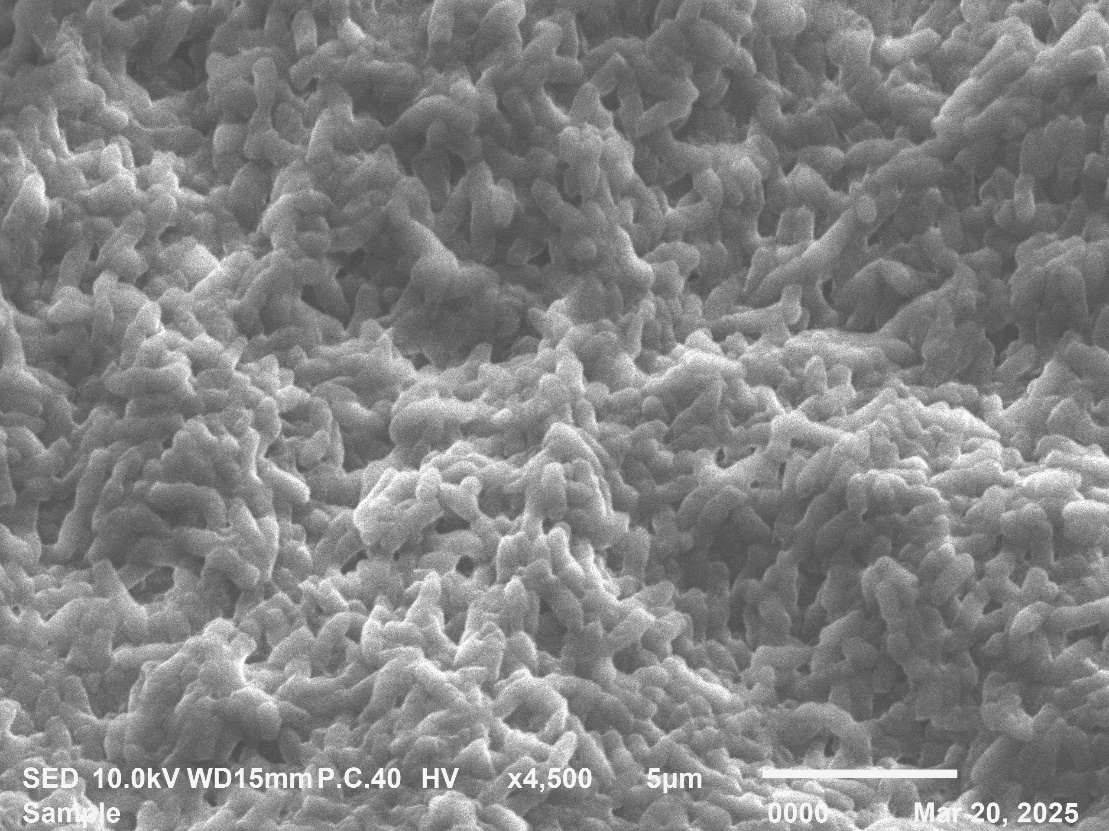

Supplement: Supplementary file 5 — Supplementary Material 5 [file 41598_2025_28607_MOESM5_ESM.docx]
