# Supplementary material for: Chitosan mitigates pan drug resistance in citrobacter freundii exhibiting AmpC and ESBL from Egyptian livestock
Source: Sci Rep. 2025 Dec 5;15:43285. doi: 10.1038/s41598-025-28607-0 (PMC12686506; doi:10.1038/s41598-025-28607-0)
Supplement: Supplementary file 6 — Supplementary Material 6 [file 41598_2025_28607_MOESM6_ESM.docx]

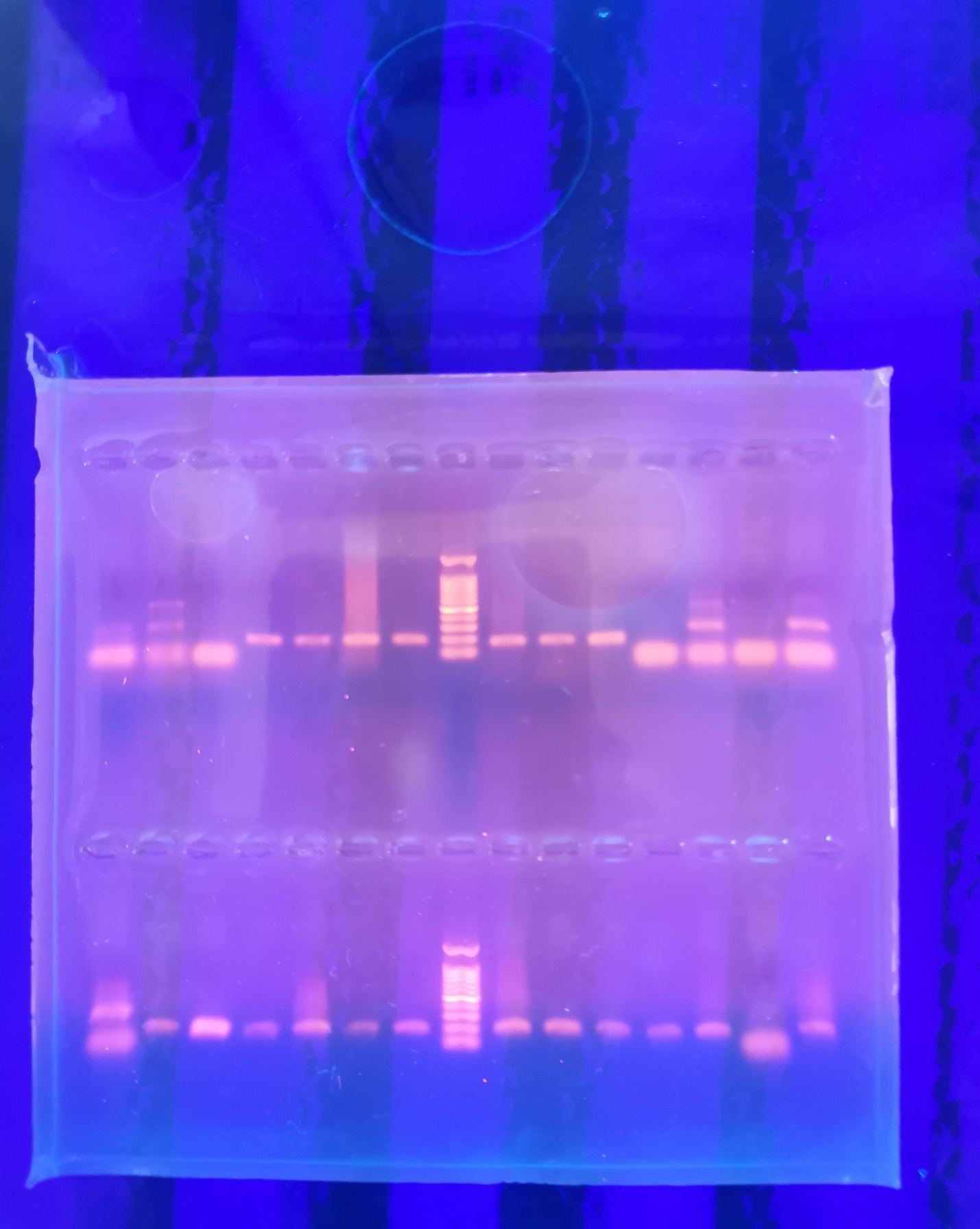


Figure 1. Agarose profile for the detection of *C. freundii* specific 23S rRNA is shown at 189 bp


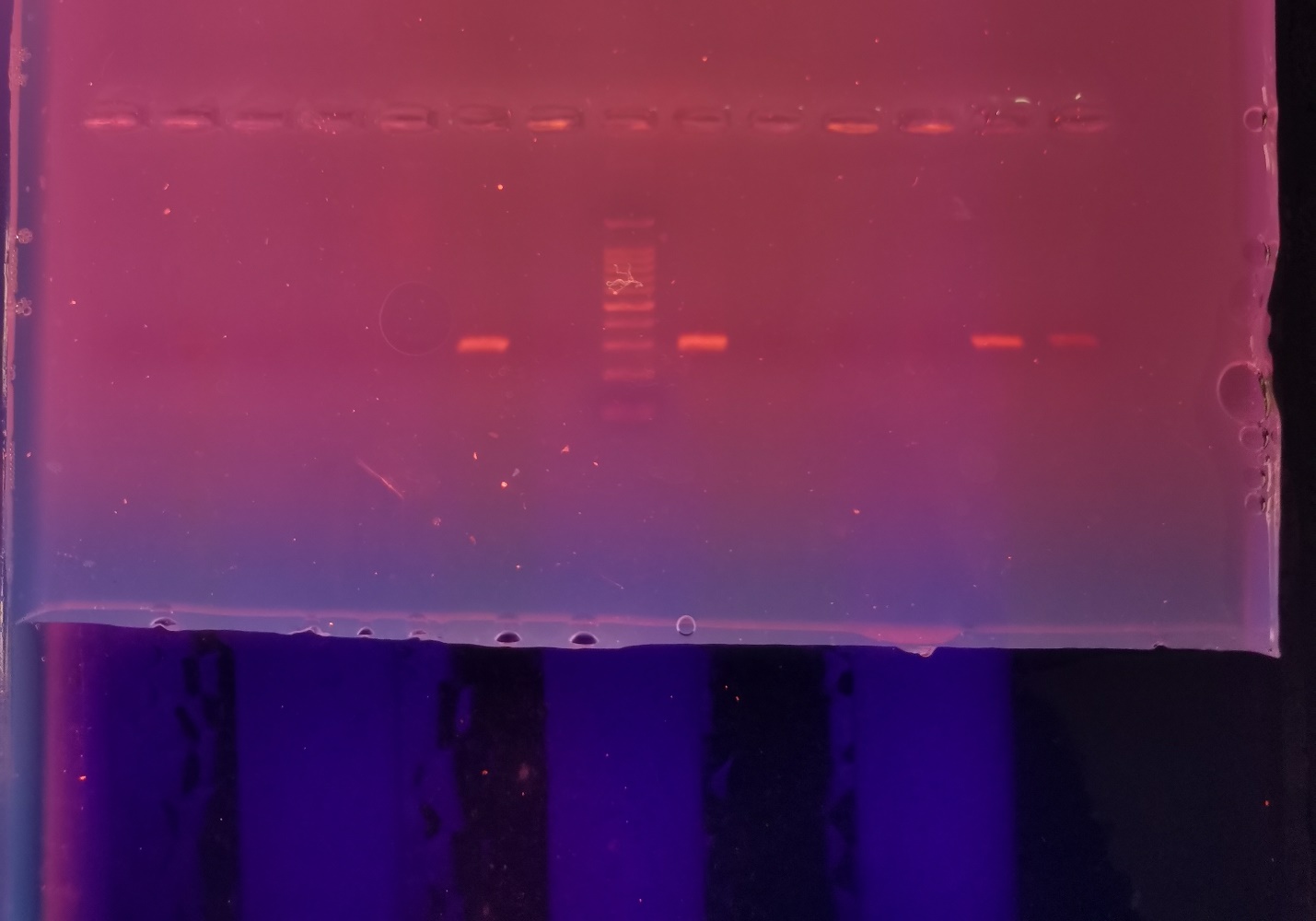


Figure 6. Agarose profile for the detection of resistance gene *mcr-*1 (309 bp)


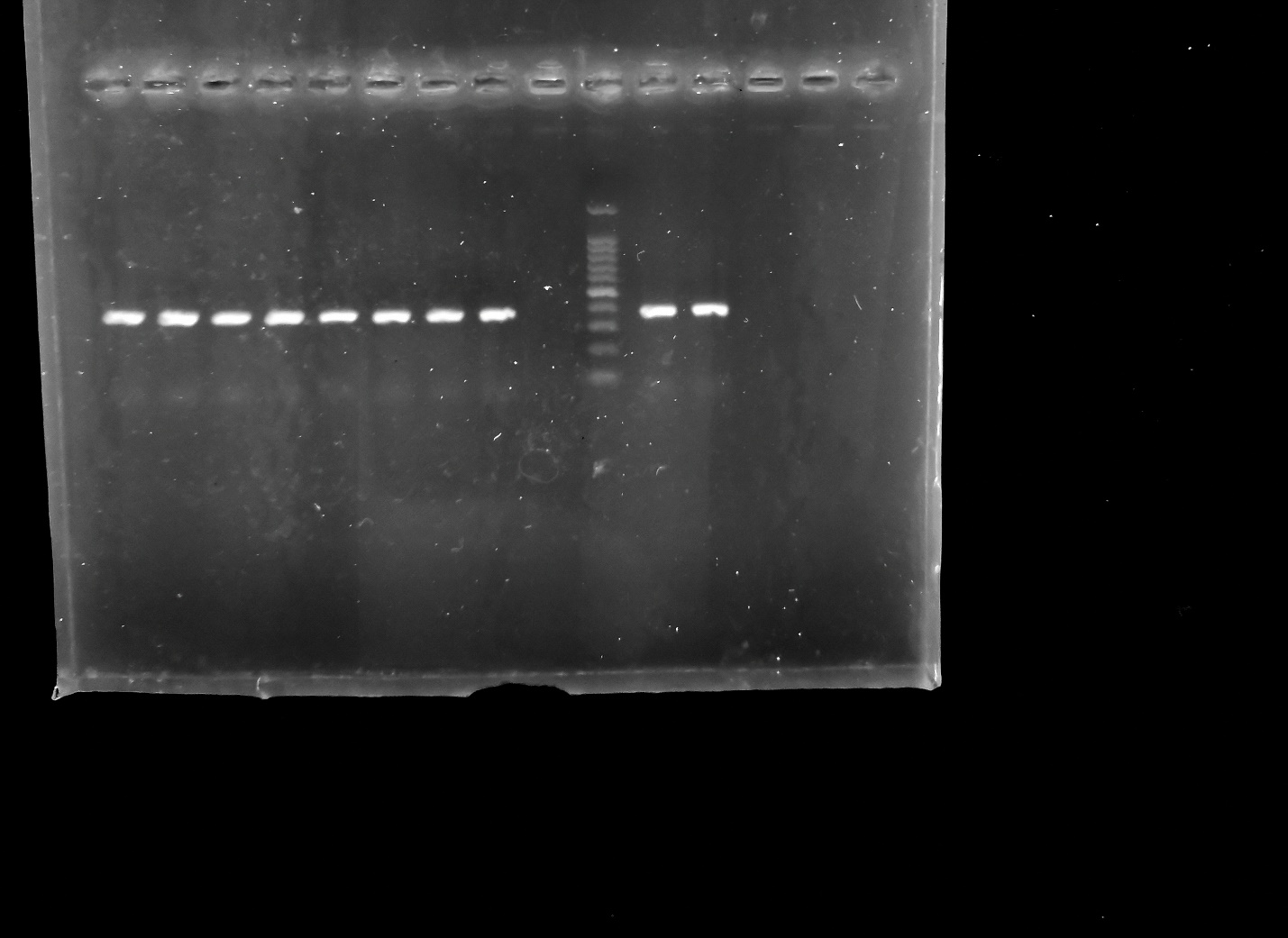


Agarose profile for the detection of resistance gene *tet(*M) (406 bp).


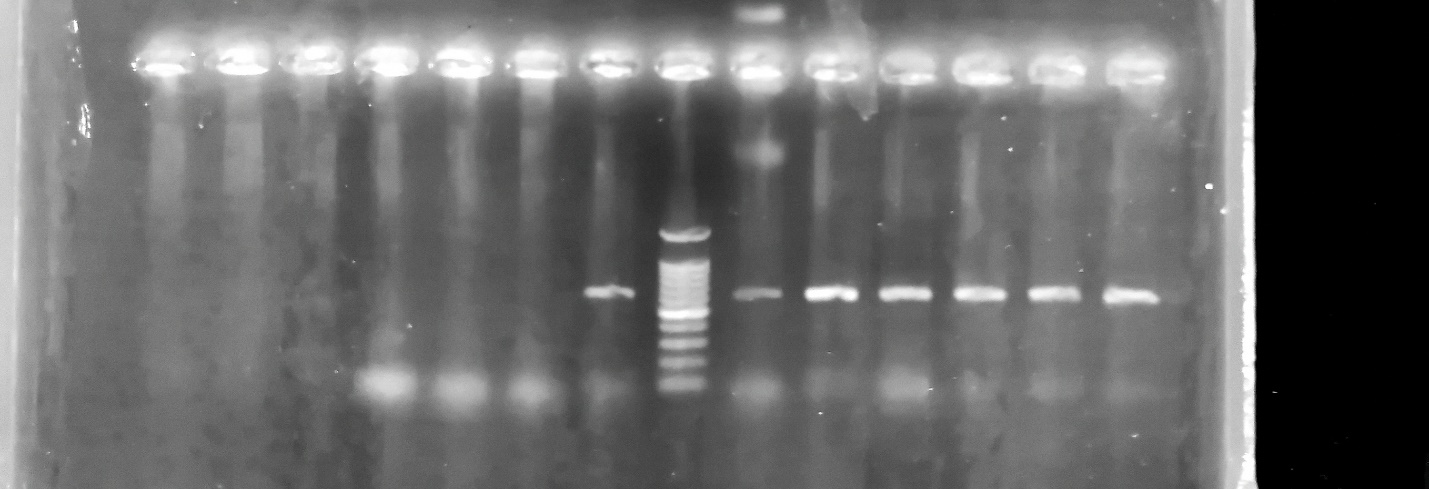


Agarose profile for the detection of resistance gene *erm*B (636 bp).


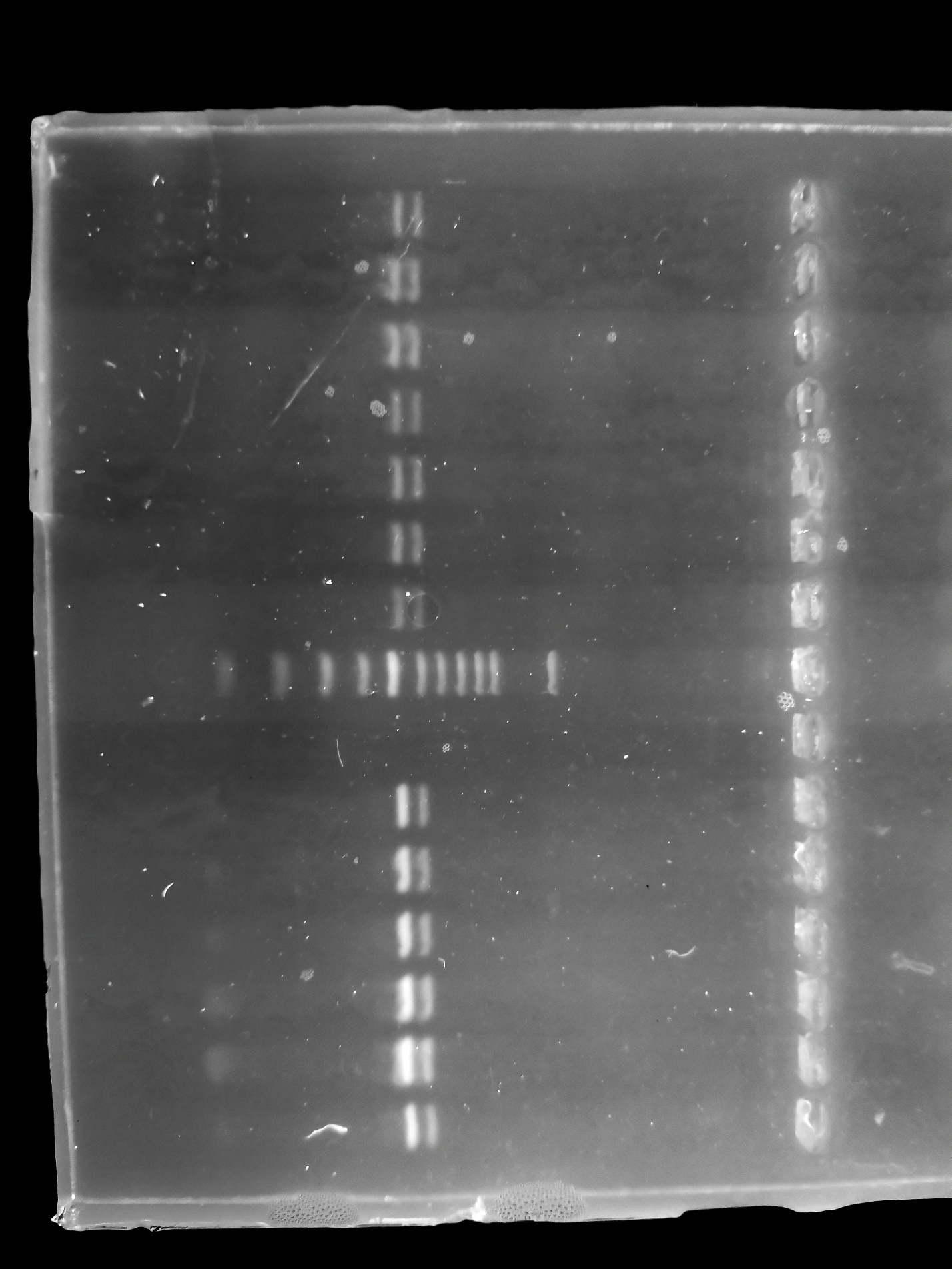


Agarose profile for the detection of resistance genes; *bla*_CTX-M_ (593 bp) and *bla*_TEM_ (516 bp) by duplex PCR


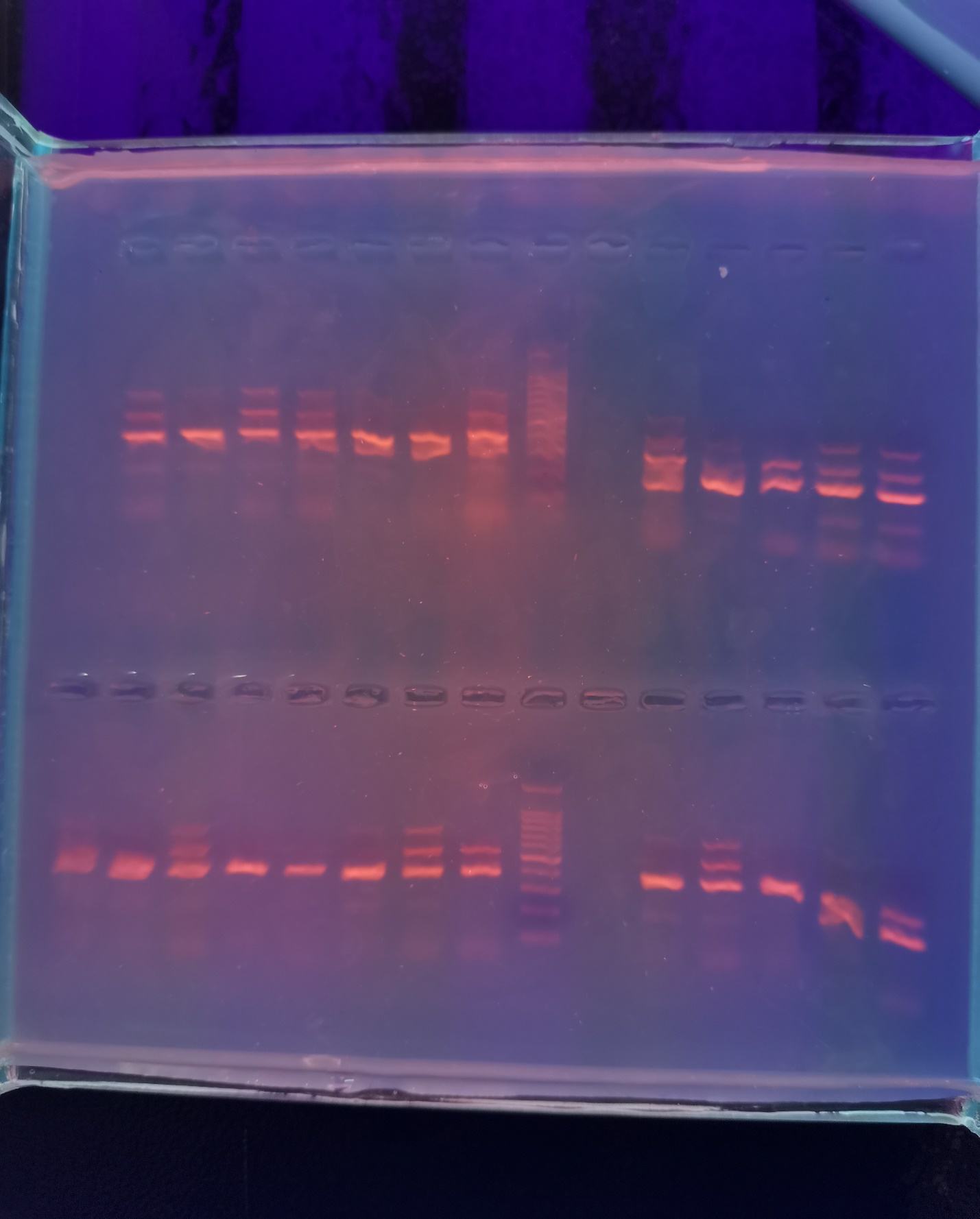


Agarose profile for the detection of resistance genes *dfr*A1 (367 bp), *qnr*A (516 bp), and *sul*2 (722 bp) by multiplex PCR.


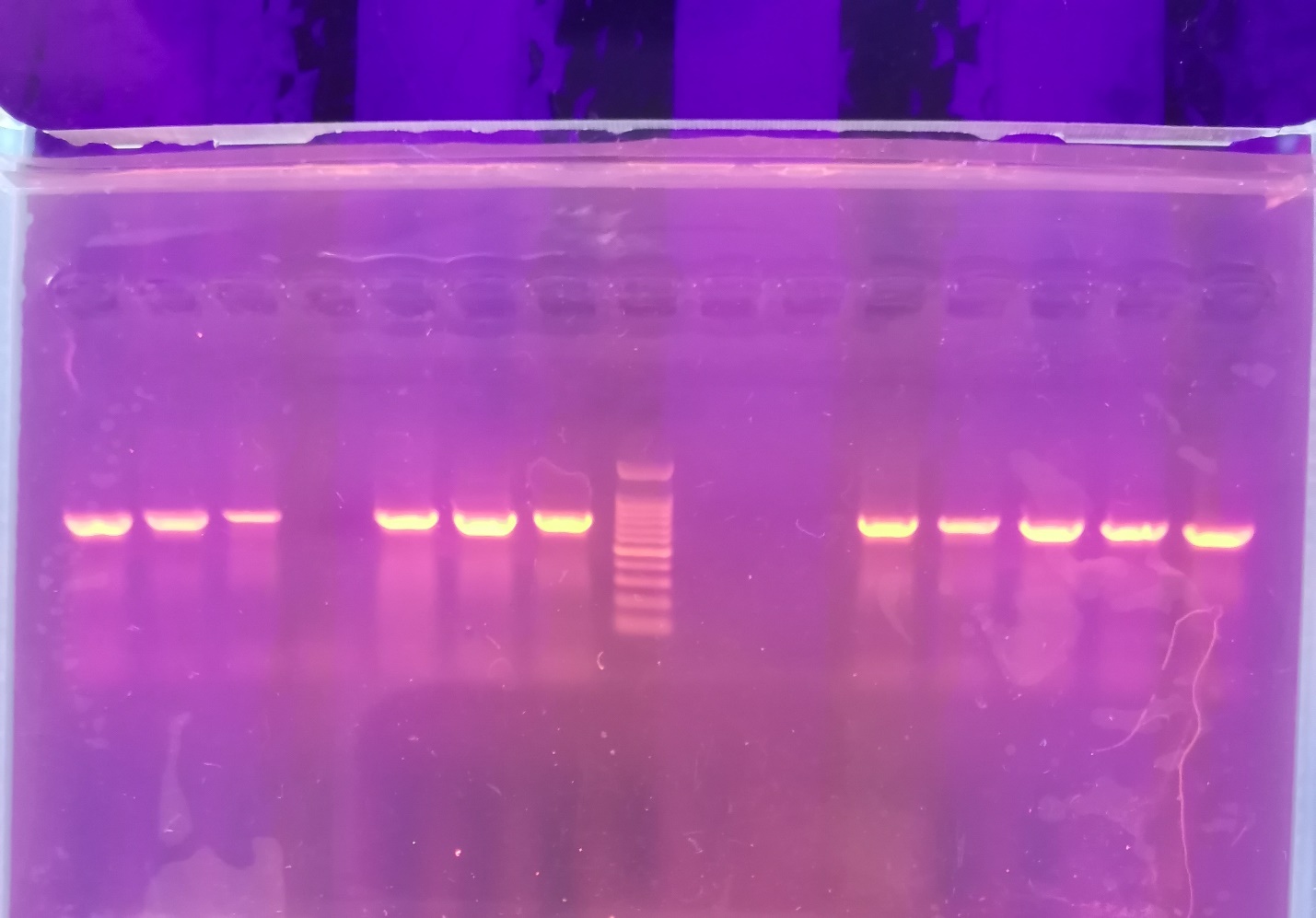


Agarose profile for the detection of *bla*_OXA-10_ (760 bp)


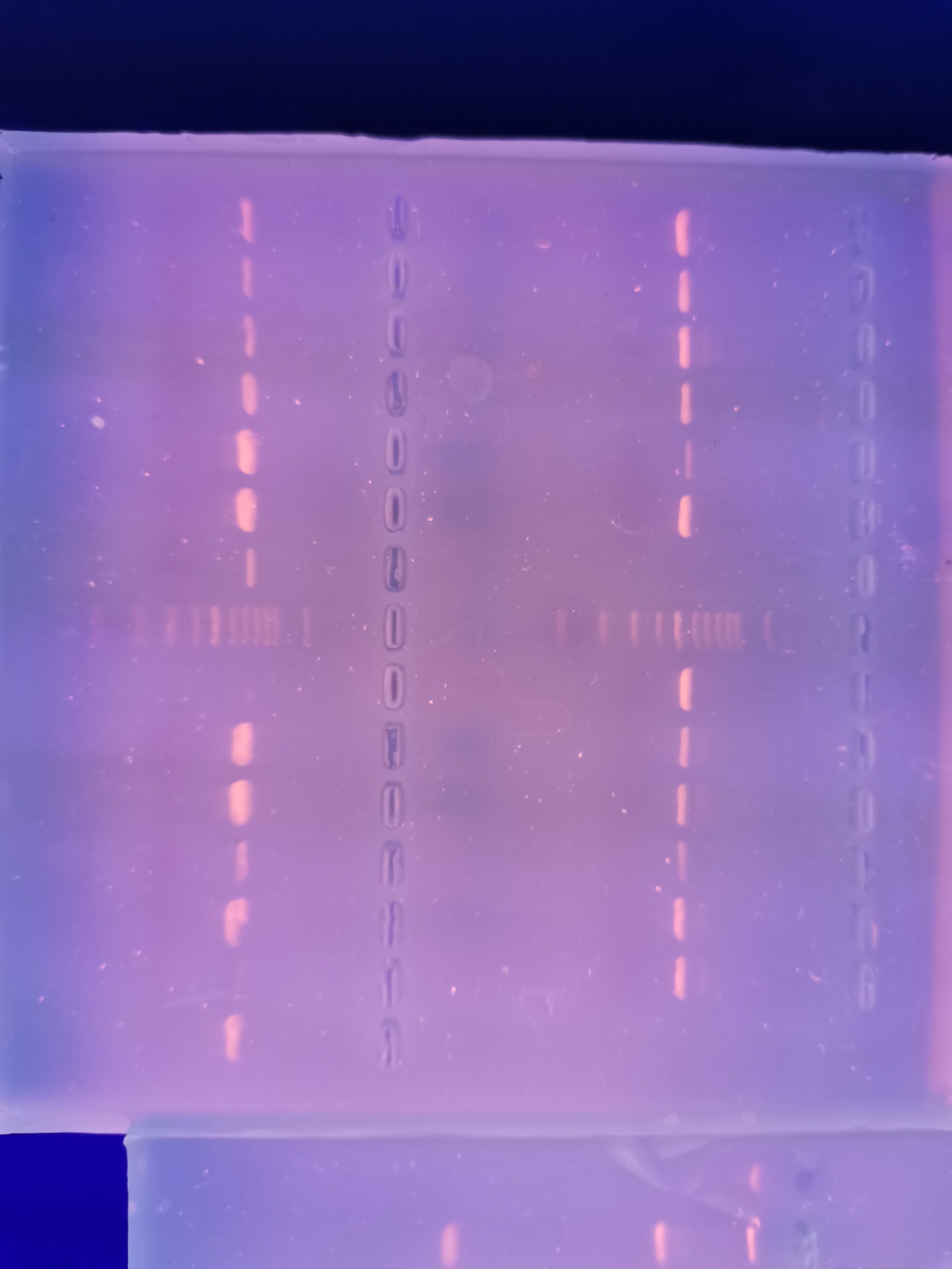


Up: Agarose profile for the detection of resistance gene *cat*A1 (547 bp).

Down: Agarose profile for the detection of resistance gene *bla*_CMY-2_ (695 bp).


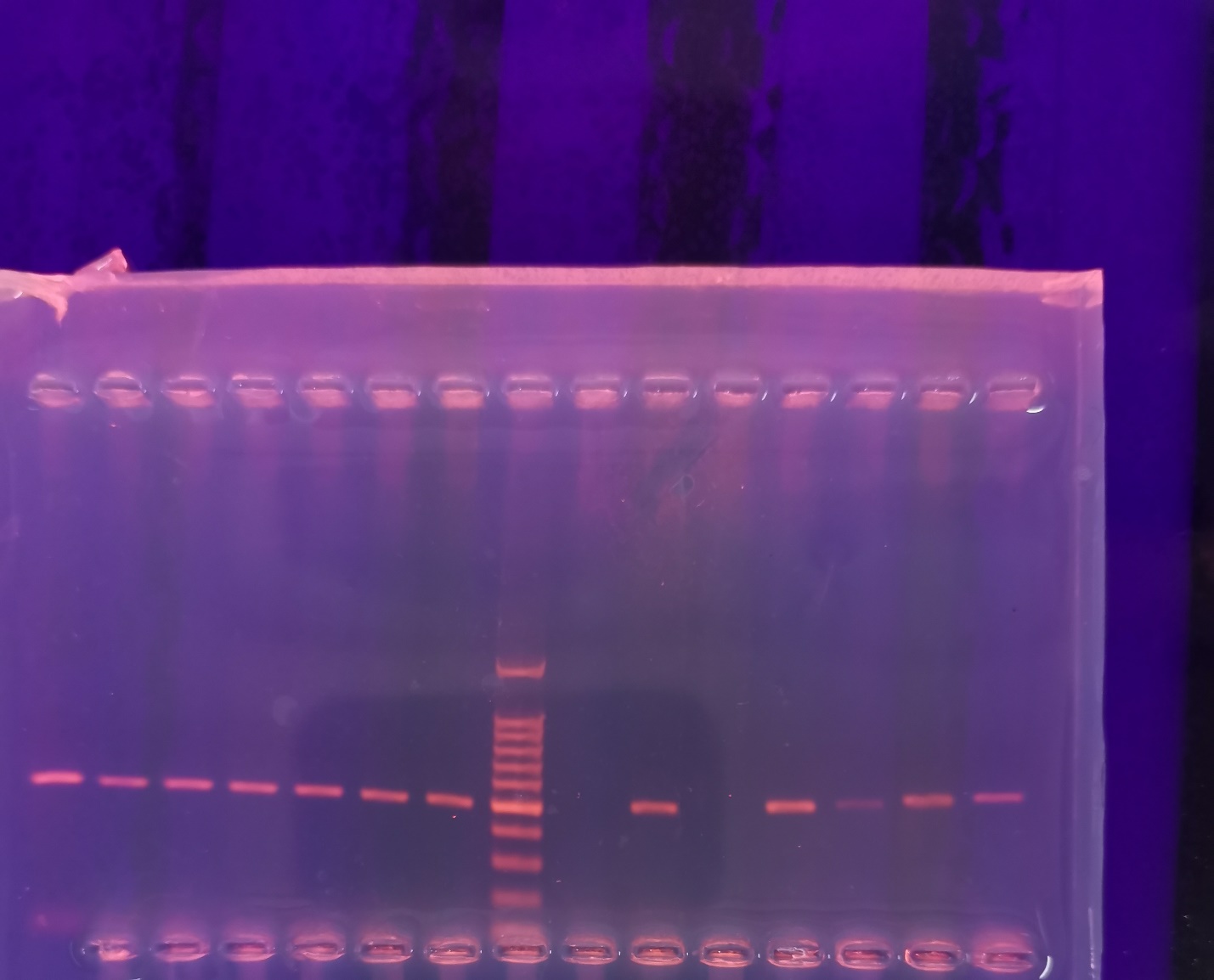


Agarose profile for the detection of resistance gene *aad*A1 (447 bp).
